# Varying molecular interactions explain aspects of crowder-dependent enzyme function of a viral protease

**DOI:** 10.1371/journal.pcbi.1011054

**Published:** 2023-04-25

**Authors:** Natalia Ostrowska, Michael Feig, Joanna Trylska

**Affiliations:** 1 Centre of New Technologies, University of Warsaw, Warsaw, Poland; 2 Department of Biochemistry and Molecular Biology, Michigan State University, East Lansing, Michigan, United States of America; Korea Institute for Advanced Study, REPUBLIC OF KOREA

## Abstract

Biochemical processes in cells, including enzyme-catalyzed reactions, occur in crowded conditions with various background macromolecules occupying up to 40% of cytoplasm’s volume. Viral enzymes in the host cell also encounter such crowded conditions as they often function at the endoplasmic reticulum membranes. We focus on an enzyme encoded by the hepatitis C virus, the NS3/4A protease, which is crucial for viral replication. We have previously found experimentally that synthetic crowders, polyethylene glycol (PEG) and branched polysucrose (Ficoll), differently affect the kinetic parameters of peptide hydrolysis catalyzed by NS3/4A. To gain understanding of the reasons for such behavior, we perform atomistic molecular dynamics simulations of NS3/4A in the presence of either PEG or Ficoll crowders and with and without the peptide substrates. We find that both crowder types make nanosecond long contacts with the protease and slow down its diffusion. However, they also affect the enzyme structural dynamics; crowders induce functionally relevant helical structures in the disordered parts of the protease cofactor, NS4A, with the PEG effect being more pronounced. Overall, PEG interactions with NS3/4A are slightly stronger but Ficoll forms more hydrogen bonds with NS3. The crowders also interact with substrates; we find that the substrate diffusion is reduced much more in the presence of PEG than Ficoll. However, contrary to NS3, the substrate interacts more strongly with Ficoll than with PEG crowders, with the substrate diffusion being similar to crowder diffusion. Importantly, crowders also affect the substrate-enzyme interactions. We observe that both PEG and Ficoll enhance the presence of substrates near the active site, especially near catalytic H57 but Ficoll crowders increase substrate binding more than PEG molecules.

## Author summary

Enzyme-catalyzed reactions in reality occur in the crowded environment of the cell. Therefore, viruses entering the host cells also encounter a crowded surrounding in which the viral enzymes are replicated. One such enzyme is the NS3/4A protease encoded by the hepatitis C virus. This enzyme is crucial for viral replication and is used as the therapeutic target for clinically approved drugs. To gain understanding of this enzyme function and explain our previous experiments on its *in vitro* activity, we performed atomistic molecular dynamics simulations in the presence of synthetic crowders (polyethylene glycol and polysucrose) mimicking the cellular crowd. Based on these simulations we describe in detail how and why these crowders affect the diffusion and structural dynamics of this enzyme and enzyme-substrate interactions. In fact, crowders enhance substrate binding, which may have vast consequences for its function in the host cell as well as drug-design.

## Introduction

The environment of cells is both crowded and confined by various macromolecules that are physically occupying up to 40% of the cytoplasmic volume [1,2]. The concentrations of the molecules found in cells, like proteins, lipids, nucleic acids, cofactors, and metabolites, reach up to 400 g/L [3]. This means that all biochemical processes in cells, including enzyme-catalyzed reactions, occur under highly crowded conditions in the presence of various background macromolecules. Importantly, these heterogenous crowders not only reduce the available space, but they also interact with each other. These nonspecific interactions can be attractive or repulsive and may exert either stabilizing or destabilizing effects on the molecules that directly participate in a particular process [4]. Therefore, the crowded surrounding impacts many properties of solutes including diffusion, bimolecular association, folding, flexibility, stability, and ligand-receptor binding [1,5]. All these may in effect change the catalytic activity of enzymes when compared to their activity in dilute solutions [6]. Typically, enzymes associate with and bind their substrates, and, after the catalysis, the reaction products dissociate. The entire process of diffusion, binding, and product release, which includes conformational changes of the reaction components, is subject to interactions with other macromolecules present in the cell.

For ease of interpreting the results, most laboratory experiments characterizing enzymatic reactions have been performed in dilute buffer solutions. However, to reflect *in vivo* conditions, more and more studies investigate the kinetics of enzymatic catalysis in crowded environments. Concentrated solutions of synthetic polymers are often used as crowding agents [6,7], to facilitate such experiments and to allow generalizations beyond effects that may be otherwise particular to a specific protein crowder. Common artificial crowders are PEG (polyethylene glycol), Ficoll (highly-branched polysucrose), and dextran (a linear flexible polyglucose). All three types of crowders are available with different molecular weights and polymer lengths to vary crowder sizes. Ficoll, formed by the copolymerization of sucrose and epichlorohydrin, is especially attractive as it is highly soluble, and has a mostly spherical shape. In contrast to protein crowders, artificial crowders are generally considered to be non-interacting with proteins. As a result, such crowders are assumed to focus on the volume-exclusion effect of crowding. However, it is impossible to have a completely inert polymer, and the focus of this work is to better understand how these model crowders may specifically interact with proteins and how such interactions may potentially impact the interpretation of experiments characterizing enzyme function under crowded conditions.

Many experimental assays have shown that kinetic rates estimated in dilute solutions can differ by orders of magnitude from the in-cell values [6,8]. For example, the PEG crowders were shown to suppress the activity of horseradish peroxidase (ten-fold increase in K_M_) but the change depended on the substrate type [9]. The ATPase hydrolytic activity of the eIF4A translation initiation factor was shown to enhance six-fold in the presence of PEG crowders [10]. PEG, dextran, and Ficoll crowders also enhanced the enzymatic activity of adenylate kinase (AK3L1) [11]. Dextran was shown to induce the activity of the ribonuclease T1 most probably because the crowder promoted the folding of this protein [12]. We have also found that the kinetic parameters of peptide hydrolysis catalyzed by trypsin [13] and HIV-1 protease [14] were affected by PEG and BSA crowders. Our experiments on another protease, NS3/4A, encoded by the hepatitis C virus (HCV), showed different effects of Ficoll and PEG, as well as BSA on the proteolytic reaction [15]. These observed differences are the main motivation for the work presented here. Since enzymes are important drug targets, it becomes crucial to understand how and why the reaction environment modulates the enzymatic rates and equilibria. In general, different factors contribute to the observed changes in crowding-induced catalytic activity such as increased concentration of the substrate due to decreased space, induced folding of the enzyme to its functional form, induced oligomerization of the enzyme to an inactive form, decreased diffusion of the reactive molecules, occupation of the substrate binding or active site by crowders, and the change of the conformation or flexibility of the binding or dimerization site.

Many of the above effects cannot be elucidated using experimental methods alone. Therefore, the impact of crowding on different solute properties and stages of enzymatic reactions has also been investigated using computational approaches [16–19]. However, apart from a few atomistic models [4,20], most simulations applied simplified crowder models that primarily mimicked only the effect of volume exclusion due to the impenetrability of molecules, e.g., [21–23]. One reason for using such simplified crowder models is computational efficiency, but the choice of such crowders also reflects the assumption that the artificial crowders mainly introduce the volume exclusion effect, and a more detailed representation of molecular-level interactions with crowders may not be needed. However, there are now many studies that describe crowder-type dependent effects on protein structure [24–27], interaction with substrates [28,29], and enzymatic function [15,30,31].

**Fig 1:**
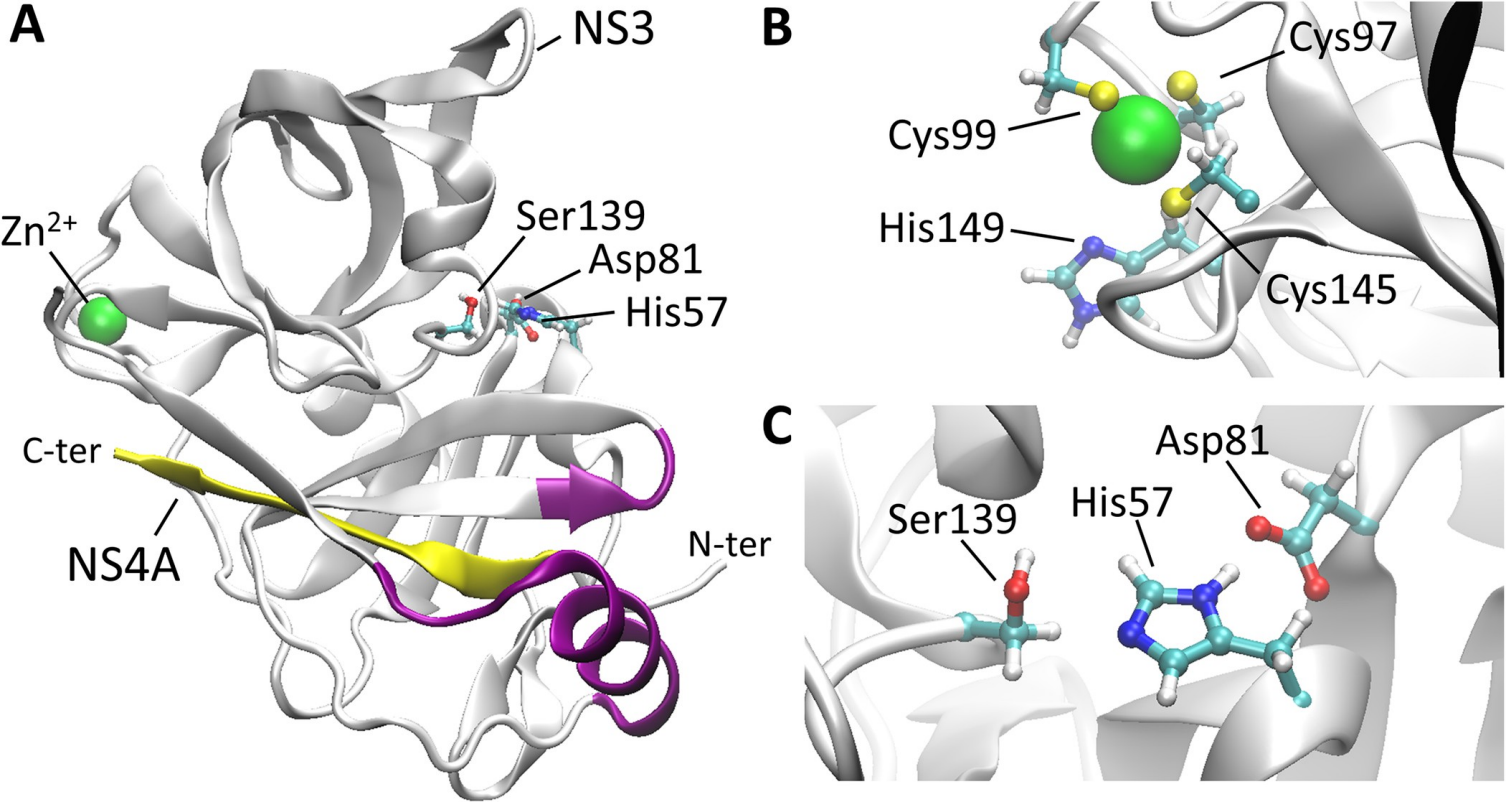
Ribbon model of the NS3/4A protease domain based on the crystal structure with PDB ID: 4JMY [36] (A). The β-sheet of the NS4A cofactor is in yellow and NS3 membrane contact sites are in magenta. The zinc coordinating residues (B) and the catalytic triad (C) are also shown.

We focus here on the NS3/4A protein complex which is a bifunctional heterodimeric enzyme composed of the serine protease and RNA helicase domains that can function independently [32]. The NS3/4A activity to process the HCV polyprotein is necessary for viral replication. HCV is responsible for a chronic illness affecting nearly 200 million people and this enzyme is the main target in HCV therapy. However, the emerging drug-resistant genotypes and subtypes of this virus necessitate the design of new inhibitors. The NS3 protease domain is a 180-residue protein, with the chymotrypsin-like fold dominated by β-sheets (**Fig 1A**). The active site contains a catalytic Ser139-His57-Asp81 triad (with the residues referred to as S139, H57, and D81 in the remainder of the manuscript). The structure is stabilized by a Zinc ion, which is at the opposite side of the active site. Zinc is coordinated by three cysteines (C97, C99 and C145) and one histidine (H149 (**Fig 1B**) that are conserved in the HCV strains [32] and mutations of any of the cysteines into alanines significantly decreased protease activity [33]. For efficient catalysis, the NS3 protease domain requires a 54 amino acid long peptide co-factor, called NS4A, but only its central part, embedded in the NS3 core, is sufficient for the *in vitro* activity of the protease. The N- and C-terminal parts of NS4A, which are not bound with NS3, are believed to be intrinsically disordered in solution. In the host cell, the NS3/4A complex associates with the endoplasmic reticulum (ER) membrane where the HCV RNA is replicated. The disordered N-terminal tail of the NS4A cofactor can be induced to form a helix that inserts into the ER membrane to anchor the NS3/4A complex [34,35].

Using fluorescence spectroscopy, we have found that PEG 6000 and Ficoll 400 differently affect (in a concentration-dependent manner) the initial and maximum velocities as well as the turnover number for the peptide hydrolysis catalyzed by the NS3/4A protease [15]. The kinetic parameters decreased in the presence of PEG and increased with Ficoll as compared to dilute buffer solutions. Ficoll also increased by 40% the inhibition constant K_i_ of telaprevir, the clinically-approved drug targeting the NS3/4A enzyme [15]. We have previously explained how PEG crowders may promote folding of the NS3/4A unstructured N-terminal fragment possibly enabling membrane anchoring [35]. However, it remains unclear how the two different crowders may impact enzyme function in an opposite manner. We hypothesize that there are specific interactions between the crowders, NS3/4A, and the substrate that differ between PEG and Ficoll, and the goal of this work is to not just explain the experimental data for NS3/4A but gain a broader understanding of how PEG and Ficoll may interact with proteins at a molecular level.

To probe these questions, we use extensive atomistic molecular dynamics simulations of NS3/4A with and without peptide substrates in the absence or presence of PEG and Ficoll-like crowders. We analyze how the crowders impact NS3/4A structure and dynamics, diffusion of the enzyme and the substrate, and how the crowders may specifically impact the substrate binding near the active site. Based on this analysis we explain how PEG and Ficoll may differently impact the kinetic parameters of the NS3/4A-catalyzed reaction and provide more general insights into how PEG and Ficoll crowders interact with peptides and proteins.

## Methods

### Preparation of solute structures

As in our previous studies [15,35], the NS3/4A crystal structure (PDB ID: 4JMY [36]) was used as the starting point for the MD simulations. This structure contains the coordinates of the full protease domain of NS3, residues 21–32 of the NS4A cofactor, the zinc ion critical for maintaining the active structure of the protease [37] and a fragment of the peptide substrate. To match the 1b genotype of HCV, the D30E, L36V, G66A, A87K, M94L, S147F, V150A, I170V, A181S, and S182P mutations were introduced using Chimera [38] with the Dunbrack rotamer library [39]. The missing N- and C-terminal residues of the NS4A cofactor were added with MODELLER [40], ver. 9.19., using a template-free modeling method. Five models of complete NS4A were generated, and, based on the Dope [41] scoring function, the lowest-scoring conformation was selected. Residues were assigned standard protonation states at pH 7, with histidine neutral and protonated at Nδ1 atom. Zinc-coordinating cysteines were modified with a specific cysteine-zinc patch to reflect deprotonation and polarized charges with coordinating cysteines modeled with a -1e charge and the Zn ion with a +2e charge [42,43]. The goal of these parameters was simply to maintain Zn coordination, whereas charge transfer and quantum corrections would need to be considered to achieve a more realistic description of zinc binding equilibria [44,45]. The net charge of the entire NS3/4A-zinc complex was +2e.

The peptide substrates with the sequence Ac-DEDEEAASK-NH_2_, corresponding to the sequence of the peptide used in experimental assays [15], were built with CHIMERA [38]. The substrate models were subjected to 2,000 steps of minimization and 20 copies of 200 ns MD simulations in NAMD [46]. Next, the substrate trajectories were clustered with ProDy [47] and the five most probable conformations were used as starting structures surrounding NS3/4A. The NS3/4A protease and substrates were parameterized using the CHARMM36m force field [48] which is suitable for simulating proteins with unstructured fragments.

### Preparation of crowder models

**Fig 2:**
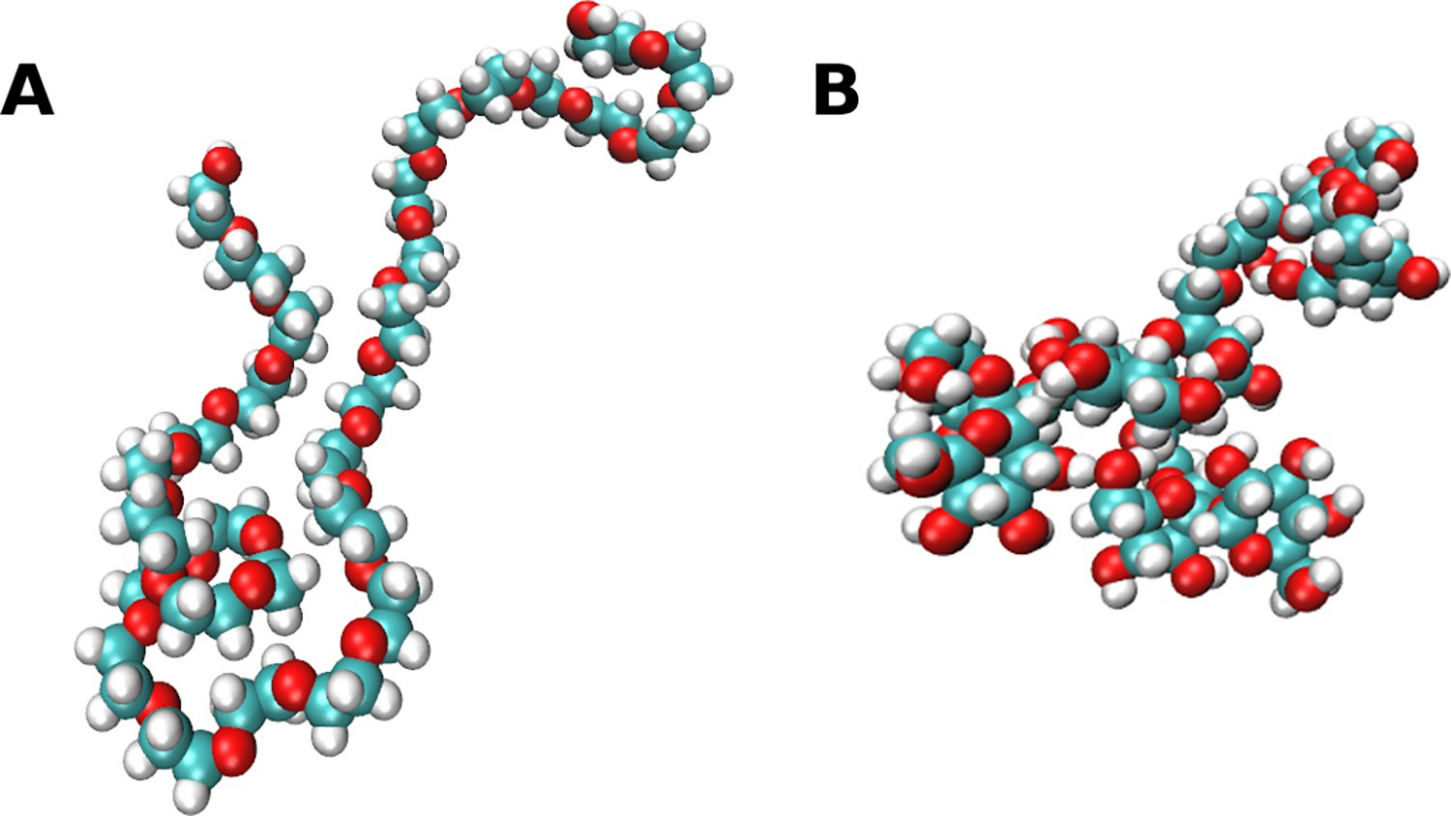
The all-atom model of PEG (A) and Ficoll (B) crowders used in MD simulations.

All-atom 28-mer PEG crowder models (**Fig 2**) were prepared as described elsewhere [15,35]. Starting conformations were taken from MD simulations of a single PEG polymer in explicit water performed by Lee et al. [49]. The CHARMM36 CgenFF force field was used with a modification of the torsion angles based on the work of Leonard *et al*. [50]. Simulations of PEG using these parameters has been found to result in good agreement with experimental observables in another study [51].

All-atom Ficoll models were built to match the mass of the PEG crowders (*cf*. **S1 Text, S1 and S2 Figs** for additional details) so that crowders with similar sizes can be compared. Our Ficoll-like molecule (referred subsequently as simply ’Ficoll’ here) consists of four sucroses and three glycerol linkers, forming the smallest possible branched Ficoll-like polysucrose (**Fig 2**). Sucrose and glycerol parameters were assigned based on the CHARMM36 force field parameters for carbohydrates [52,53]. Additional patches were constructed for sucrose-glycerol connections, with parameters derived by analogy to already parameterized polysaccharides in the CHARMM force field, e.g., using parameters for isomaltulose and melezitose (*cf*. **S1 Text, S3 Fig**). To obtain the Ficoll starting conformation for the simulations of the crowded systems, one tetra-sucrose was energy-minimized and an MD simulation in explicit TIP3P water was carried out over 200 ns. The starting Ficoll crowder conformations were randomly chosen from the 20% of the MD snapshots with the lowest R_g_ values.

### Simulation systems

**Fig 3:**
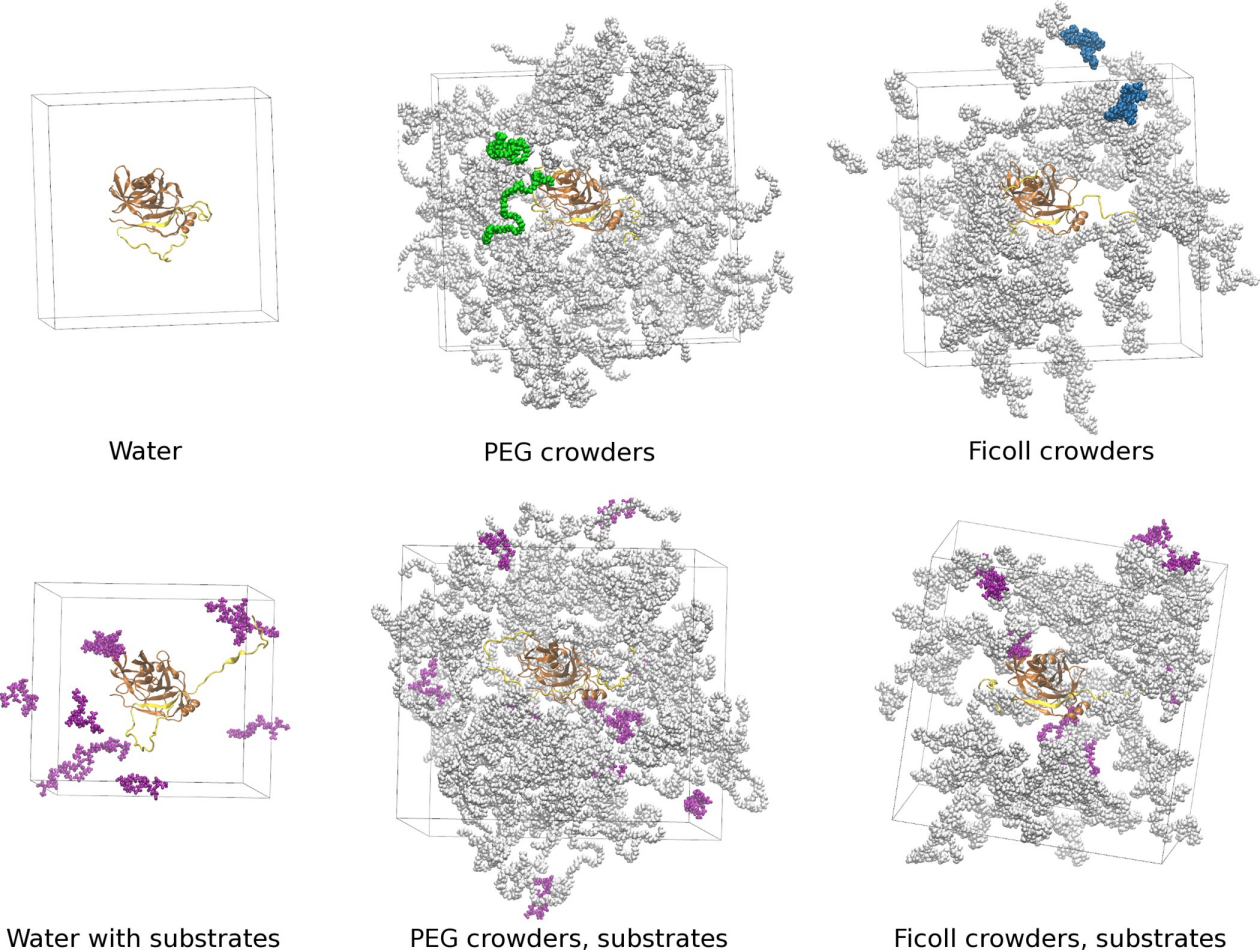
Snapshots of the systems simulated in this study. The NS3/4A enzyme is shown in brown. Substrates are shown in purple. Crowder molecules are shown in grey, but selected molecules are colored to indicate their size (PEG: green, Ficoll: blue). For clarity of the figure, water molecules and ions are not shown.

Six types of systems were simulated: NS3/4A protease with and without 10 peptide substrates, NS3/4A surrounded by 130 PEG polymers, with and without the substrates, NS3/4A surrounded by 110 Ficoll polymers, with and without the substrates (see **S1 Table**). All simulations were performed with explicit TIP3P water molecules and 2 Cl^-^ ions to neutralize the Zn^2+^ ion coordinated by NS3. Na^+^ ions were added to neutralize the simulations with 10 substrates (each substrate has a net charge of -4e). Additional Na^+^ and Cl^-^ ions were added to achieve an ionic strength of ~20 mM. The MMTSB Toolset [54] was used to solvate the systems and add ions. Initial snapshots of the simulation systems are shown in **Fig 3**.

Before solvating the systems, 130 copies of PEG or 110 copies of Ficoll molecules were added in random locations around NS3/4A. Three different sets of crowder positions were generated. The numbers of crowder molecules were chosen to match the mass concentration of crowders; the resulting volume fractions of crowders varied between systems, ranging from 11 to 15%. To avoid clashes, an additional step was carried out for the protease-PEG systems. They were minimized in vacuum before adding water molecules. Considering the high degree of crowding, substrates were added manually using the VMD [55] tools for the systems comprised of both crowders and substrates.

### Molecular dynamics simulations

The simulations were performed for the systems listed in **S1 Table**. Each system was first energy-minimized over 3,000 steps using the steepest descent algorithm. Second, water molecules and ions were thermalized by increasing the temperature from 10 to 310 K in steps of 10 K. Each step was simulated for 25 ps, with 10 kcal/mol/Å^2^ harmonic constraints on the positions of the solute atoms. Third, the restraints on the solute were decreased in six 25-ps steps with harmonic force constants gradually decreasing from 10 to 5, 2, 1, 0.1, and 0 kcal/mol/Å^2^. These thermalization and equilibration steps were performed in the NVT ensemble, with a time step of 1 fs. Finally, an additional equilibration step of 25 ps was performed in the NPT ensemble with a time step of 2 fs using the SHAKE algorithm [56] to constrain bonds made by hydrogen atoms. For each system, a 500 ns production simulation was performed either using NAMD 2.11 [46] or OpenMM [57]. A temperature of 310 K and a pressure of 1 atm were maintained with the Langevin thermostat and Langevin piston methods [58]. Two friction coefficients in the Langevin thermostat were used: 1 ps^-1^, the value used in our previous simulations [15,35], and 0.01 ps^-1^. The lower value gives more accurate estimates of kinetic properties such as diffusion [59,60]. All trajectories were used to evaluate thermodynamic properties but only the trajectories obtained with a value of 0.01 ps^-1^ were used to analyze diffusive properties and contact life-times. To calculate the electrostatic interactions, the Ewald summation [61] method was used, with a cut-off of 12 Å. To avoid aggregation of the crowders, interactions between the solute and explicit water molecules were increased by a factor of 1.09 because it was found that this modification ensures realistic diffusion of the crowders [35,60].

Multiple replicates of each system were simulated over at least 500 ns. The NS3/4A enzyme was stable in all simulations as indicated by Cα root-mean square deviation (RMSD) values generally between 1 and 2 Å throughout the trajectories (**S4 Fig**). Replicate-averaged RMSD values increased significantly during the first tens of nanoseconds, but they changed slowly afterwards (**S5 Fig**). Therefore, we decided to discard the first 50 ns as equilibration from all analyses. To compare the same simulation intervals, all trajectories were analyzed over 50–500 ns, even though some simulations were somewhat longer (**S1 Table**).

### Trajectory analysis

RMSD and RMSF values were calculated for the Cα atoms, after NS3/4A superposition onto the reference crystal structure as a starting point.

Translational diffusion was calculated from the mean-square displacements (*MSD*) of the centers of mass of the molecules. Diffusion coefficients, D_0_, were calculated from the slopes of linear fits to *MSD*(τ) = ⟨(**r**(t+ τ) − **r**(t))^2^⟩ according to the Einstein equation:

$$D_{0}=\frac{MSD(\tau )}{6\tau }$$ (1)

where **r**(t) and **r**(t+τ) are the vector positions of the center of mass of a molecule in time t and t+τ (τ is the time lag). D_0_ values were corrected for the artifacts resulting from using the periodic boundary conditions (PBC) [62] and reduced viscosity of the TIP3P water model (as the ratio of the shear viscosity of TIP3P equal to 0.308 cP and bulk water—0.89 cP) giving:

$$D_{corrected}=\frac{3.08}{8.9}(D_{0}+D_{PBC})$$ (2)

The correction *D*_*PBC*_ was calculated as:

$$D_{PBC}=\frac{{Tk}_{B}}{6L\pi \eta }\left(\zeta -\frac{4{\pi R}_{h}^{2}}{3L^{2}}\right)$$ (3)

where T is the temperature set to 310 K, *k*_*B*_ is the Boltzmann constant, ς is equal to 2.837, *R*_*h*_ is the hydrodynamic radius of a molecule (calculated with HYDROPRO [63]), and *L* is the length of the simulation box. *η* = *η*_*w*_(1+2.5*φ*) describes the shear viscosity of the solvent as the viscosity of the TIP3P water scaled with a factor depending on φ, i.e., the volume fraction of crowders.

Rotational diffusion of the NS3/4A protease was determined using the method introduced by Case *et al*. [64]. First, a trajectory of a 1,000 unity vectors pointing in random directions was merged with a trajectory of the centered protease. Second, the vectors were rotated along with the protease rotations by fitting the protease in each frame to a reference structure. To calculate the rotational correlation times, the average correlation function for the vectors was then fitted to a double- exponential function:

$$f=a\;exp\left(\frac{-x}{\tau_{1}}\right)+(1-a)\;\exp\;\left(\frac{-x}{\tau_{2}}\right)$$ (4)

Obtaining the τ_1_ and τ_2_ times describing rotations in longer and shorter time-scale, with *a* being the weight corresponding to the shorter rotational correlation time. Rotational diffusion coefficients were related to calculated correlation times as:

$$D_{r}=\frac{3.08}{8.9}\frac{1}{6\tau }$$ (5)

where the 3.08/8.9 is the scaling factor included to account for the reduced viscosity of the TIP3P water model (see above) and the overall t was calculated according to:

$$\tau ={\left(\frac{a}{\tau_{1}}+\frac{\left(1-a\right)}{\tau_{2}}\right)}^{-1}$$ (6)

Correlation analysis was used to estimate the time scales of contacts between the molecules and of the dynamics of active site residues. For contact analysis, a contact function was used to describe whether a contact between the molecules present at time t_0_ is also present at time t_0_+Δt:

$$P(\Delta t)=\frac{1}{N-k}\frac{1}{N_{p}}\sum_{j=1}^{N-k}\sum_{i=1}^{N_{p}}\delta_{i}(t_{j})\delta_{i}(t_{j}+\Delta t)$$ (7)

where *N* is the total number of trajectory frames, Δt is the *k*-th time interval and *N*_*p*_ is the number of molecule pairs, and the summation runs over *j* trajectory frames and *i* pairs of molecules. The function *δ*_*i*_*(t)* takes the value of 1 when the distance between the *i*-th pair of molecules at time *t* is smaller than 5 Å, and 0 when the molecules are further away from each other. To obtain estimated times of contact survivals, the contact functions were fitted to double exponential functions according to Eq 4, but including an additional constant to allow for contacts persisting beyond the length of the simulations.

The above analyses, along with radii of gyration and radial distribution functions, were conducted using the MMTSB Toolset [54], CHARMM version 46b2 [65], and in-house scripts, in particular to automate efficient data processing. The MMTSB Toolset was also used to cluster substrate binding poses. VMD was used to prepare the structural figures and analyze the hydrogen bonds, with the maximum donor-acceptor distance set to 3.5 Å and the donor-hydrogen-acceptor angle criteria set to 120 degrees. Secondary structures were analyzed using the Stride algorithm [66]. Plots were generated using scripted Gnuplot, version 5.2 [67].

## Results and discussion

The analysis of the simulations is organized by first describing the structural stability, conformational dynamics, and diffusive properties of NS3/4A as a function of crowders. We then describe how different crowders interact with NS3/4A and finally how crowders affect the substrates and substrate-enzyme interactions. Finally, we analyze the interaction of water molecules with NS3/4A and the crowders. All of the results are then interpreted in the context of the experimentally observed functional differences of the enzyme in the presence of different crowders.

### Structural stability of NS3/4A in the presence of crowders

The structure of the NS3/4A enzyme remained stable throughout the simulations with average Cα RMSD values of about 1.6 Å for NS3 and the 13 residues of NS4A resolved in the crystal structure (**S4 Fig** and **S2 Table**). RMSD values appeared to be slightly larger in the presence of Ficoll or PEG crowders compared to water; in the presence of substrates, RMSD values were smaller with Ficoll and larger with PEG. However, in all cases, the differences in RMSD values were on the order of the standard errors and therefore not considered significant.

Average radius of gyration values *R*_*g*_ of 16.2 Å for NS3 (**S3 Table**) were slightly larger than the value of 15.8 Å calculated from the experimental coordinates based on 4JMY. Again, there were only slight differences in the absence or presence of crowders, but it appears that the distribution of *R*_*g*_ values is shifted to slightly larger values in the presence of Ficoll (**S6A Fig**) compared to dilute solvent or PEG crowders. However, in the presence of substrates, NS3 is slightly more compact with both PEG and Ficoll crowders (**S6B Fig**).

NS4A has flexible N- and C-termini which result in larger *R*_*g*_ values and larger fluctuations (**S6C Fig**). Average R_g_ values of NS4A appear to be smaller with Ficoll and larger with PEG compared to the system without crowders (**S3 Table**); with substrates the opposite trend is found, i.e., NS4A is less compact in the presence of Ficoll and more compact with PEG (**S6D Fig**). However, all of the differences are well within the standard errors and therefore not considered significant.

The presence of crowders is generally believed to favor more compact conformations, especially for more dynamic structural elements. However, for the system studied here, the effect of the crowders on the NS3/4A structure is very modest if any, including for the extended NS4A termini.

### Conformational dynamics of NS3/4A in the presence of crowders

Since NS3 remained overall stable, no major conformational changes were observed. The remaining conformational dynamics characterized via root-mean square fluctuations (RMSF) around the trajectory-averaged structures (**S7 Fig**) shows fluctuations for most residues below 1.5 Å, with increased fluctuations of up to about 3 Å for more dynamic regions on the surface of NS3 (**S8 Fig**) corresponding to loops and solvent-exposed secondary structure elements. The overall pattern of fluctuations is similar with and without crowders, but the simulation results suggest some differences (**S7 Fig**).

Without substrates, Ficoll crowders appear to induce greater conformational dynamics compared to dilute solvent at the beginning of the N-terminal helix, residues 12–15 (average RMSF of 2.10 +/- 0.26 Å^2^ vs. 1.35 +/- 0.11 Å^2^, P = 0.04 for two-tailed hypothesis from Welch’s t-test), at the loop at residues 89–90 (average RMSF of 1.32 +/- 0.21 Å^2^ vs. 1.15 +/- 0.07 Å^2^, P = 0.45), and at the β-turn involving residues 119–123 (average RMSF of 1.69 +/- 0.22 Å^2^ vs. 1.35 +/- 0.25 Å^2^, P = 0.35), while reducing conformational dynamics in the β-strand linker at residues 74–75 (average RMSF of 0.73 +/- 0.05 Å^2^ vs. 0.88 +/- 0.03 Å^2^, P = 0.05), in residues 95–100 (average RMSF of 0.82 +/- 0.04 Å^2^ vs. 0.98 +/- 0.05 Å^2^, P = 0.05), and the β-turn at residues 146–149 (average RMSF of 0.63 +/- 0.02 Å^2^ vs. 0.76 +/- 0.04 Å^2^, P = 0.03). The regions 97–99 and 145–149 include the residues that coordinate the zinc ion (C97, C99, C145, and H149, **Fig 1B**). PEG crowders affect conformational dynamics less, they also increase fluctuations in the loop at residues 89–90 (average RMSF of 1.59 +/- 0.25 Å^2^ vs. 1.15 +/- 0.07 Å^2^, P = 0.14), but different from Ficoll, they may decrease fluctuations in the β-turn around at residues 119–123 (average RMSF of 1.17 +/- 0.23 Å^2^ vs. 1.35 +/- 0.25 Å^2^, P = 0.63).

This suggests that both crowders perturb the conformational sampling of NS3, Ficoll more than PEG, but with the detailed effects of the two types of crowders being qualitatively different. Previous work has suggested that altered conformational fluctuations in the presence of crowders may correspond to different overall stabilities of the folded state in the presence of the crowders [68]. Some of these effects are retained when substrates are introduced into the systems (**S7B Fig**), but together with substrates, PEG has a greater tendency to lead to increased fluctuations, for example in the entire N-terminal helix (residues 12–23), and near residue 89, whereas Ficoll with substrates results in more similar fluctuations than without Ficoll. The NS3 N-terminal helix (**Fig 1A**, magenta) is essential for membrane association of the NS3/4A complex [34] and both PEG and Ficoll seem to increase its conformational dynamics.

The cofactor NS4A has flexible N- and C-termini giving rise to greater RMSF values (**S9 Fig**). Crowders somewhat reduce the fluctuations, but without substrates the effect is only statistically significant for the N-terminus. When substrates are present, the fluctuations in the NS4A termini are reduced more in the presence of crowders, with no clear differences between PEG and Ficoll (**S9B Fig**).

We previously described the ability of PEG crowders to induce helical structures in the N-terminus of PEG [35]. Such helix formation prepares for membrane anchoring of NS3 and thus we have previously concluded that cellular crowding may assist with this process [35]. Based on the simulations presented here, we again find a greater tendency towards helix formation in the N-terminus of NS4A in the presence of PEG crowders (**S10 Fig**, **S4 Table**). Without substrates, Ficoll does not appear to induce helical conformations to the same extent. However, when substrates are present, both PEG and Ficoll induce significantly more helical structures in NS4A compared to dilute solutions, and PEG induces more helical structures than Ficoll (**S11 Fig**, **S4 Table**). Similar conclusions are also found when analyzing the φ/ψ peptide backbone torsion angle distributions in the N- and C-termini of NS4A (**S12** and **S13 Figs**). This confirms an overall role of crowding in promoting functionally relevant secondary structure formation in NS4A, but also indicates that this observation may depend on the choice of the crowder molecule used to mimic cellular environments.

### Self-diffusion of NS3/4A in the presence of crowders

Translational and rotational diffusion was analyzed from the trajectories with the reduced friction coefficients as described in the Methods section. Translational diffusion coefficients were obtained from linear fits to the mean-square displacement curves shown in **S14 Fig** followed by corrections for periodic boundary artifacts and reduced viscosity of the TIP3P water model. The results are given in **Table 1**. As expected, the presence of crowders significantly reduces the diffusion rates of NS3/4A by about one third from diffusion in dilute aqueous solvent. Interestingly, diffusion is reduced more in the presence of PEG than in the presence of Ficoll. When substrate molecules are present, diffusion is reduced further, both without and with crowders and, again, diffusion is slower with PEG crowders than with Ficoll crowders when substrate is present. The substrate molecules therefore act themselves as additional crowders with substrate and crowders apparently affecting translational diffusion of NS3/4A in an independent, additive manner.

Rotational diffusion coefficients were obtained from double-exponential fits to rotational correlation functions shown in **S15 Fig**. The resulting rotational time scales are given in **S5 Table**. Overall rotational diffusion rates, corrected for the faster viscosity of TIP3P water, are reported in **Table 2**. Different from translational diffusion, the presence of crowders alone appears to affect rotational diffusion rates of NS3/4A less. Diffusion actually appears to be slightly higher with PEG, although this result is based on only two simulations (**S1 Table**). Moreover, this is a result of slightly faster rotation on fast time scales (3.4 ns with PEG vs. 3.7 ns with water) whereas rotation on slower time scales is retarded with PEG (28.2 ns with PEG vs. 22.7 ns with water) according to the double-exponential fits (**S5 Table**). Double-exponential fits resulted in significantly better fits to the data than single-exponential fits as indicated by lower χ^2^ values (**S5 Table**). This suggests that rotational diffusion involves multiple time scales, likely because of conformational changes of the NS3/4A complexes as NS4A undergoes significant fluctuations and due to transient enzyme-crowder interactions.

With Ficoll crowders, rotational diffusion of NS3/4A is slowed down more. The presence of substrates reduces rotational diffusion slightly without crowders, but there is a more significant reduction when either PEG or Ficoll crowders are present along with substrates.

The suggestion that translational and rotational diffusion of NS3/4A may be affected differently in the presence of crowders, led us to further look for evidence of anomalous diffusion, *i*.*e*., a variation of diffusion rates over different time scales. This could result, for example from a cage effect where crowders do not hinder short-term motion and rotational motions in place but present molecular obstacles when diffusing over longer distances. Based on plots of log(MSD/t) vs. time (**S16 Fig**) we found that the presence of the crowders does indeed give rise to anomalous diffusion since log(MSD/t) decreases significantly from a peak at around 50 ps towards longer time scales. This effect appears to be stronger with PEG than with Ficoll, both without and with substrate (**S16 Fig**).

Diffusion of the crowders themselves is also significantly different. Translational diffusion of PEG is significantly slower than that of Ficoll (**Table 1**). This is easily explained by the larger average size of PEG (**S3 Table**) compared to Ficoll, but PEG again appears to rotate faster than Ficoll (**Table 2**), potentially indicating differences in crowder-crowder interactions. Finally, substrate diffusion is reduced upon crowding and follows crowder diffusion rates, both for translational and rotational diffusion. For example, substrate diffusion is reduced significantly more in the presence of PEG than in the presence of Ficoll. This suggests that substrates may spend a significant time near crowders and effectively move together. This will be discussed in more detail below.

**Table 1:** Translational diffusion of NS3/4A, crowders, and substrates.

	Translational diffusion [Å^2^/ns]		
	NS3/4A	Crowder	Substrate
**Water**	9.40 *(0*.*02)*		
**PEG**	6.02 *(0*.*13)*	14.83 *(0*.*09)*	
**Ficoll**	6.75 *(0*.*22)*	20.30 *(0*.*10)*	
**Substrate**	8.02 *(0*.*26)*		20.54 *(0*.*39)*
**PEG/Substrate**	5.29 *(0*.*39)*	13.92 *(0*.*08)*	13.73 *(0*.*45)*
**Ficoll/Substrate**	5.96 *(0*.*02)*	18.69 *(0*.*10)*	16.25 *(0*.*51)*

Averages based on linear fits to MSD curves in **S14 Fig** from 1 to 10 ns over replicate trajectories with a reduced friction coefficient (see **S1 Table**) after corrections for periodic boundaries and TIP3P water model. Standard errors of the mean are given in parentheses.

**Table 2:** Rotational diffusion of NS3/4A, crowders, and substrates.

	Rotational diffusion [1/ns]		
	NS3/4A	Crowder	Substrate
**Water**	0.0170 *(0*.*003)*		
**PEG**	0.0179 *(0*.*003)*	0.281 *(0*.*002)*	
**Ficoll**	0.0137 *(0*.*001)*	0.226 *(0*.*001)*	
**Substrate**	0.0164 *(0*.*007)*		0.288 *(0*.*011)*
**PEG/Substrate**	0.0091 *(0*.*002)*	0.279 *(0*.*002)*	0.256 *(0*.*018)*
**Ficoll/Substrate**	0.0112 *(0*.*005)*	0.219 *(0*.*001)*	0.245 *(0*.*086)*

Results from double-exponential fits to combined correlation functions from replicate trajectories with reduced friction coefficient (see **S1 Table**), corrected for the TIP3P water model. Standard errors of the mean given in parentheses were estimated from variations between fits to individual correlation functions.

### Interaction of NS3 with crowders

**Fig 4:**
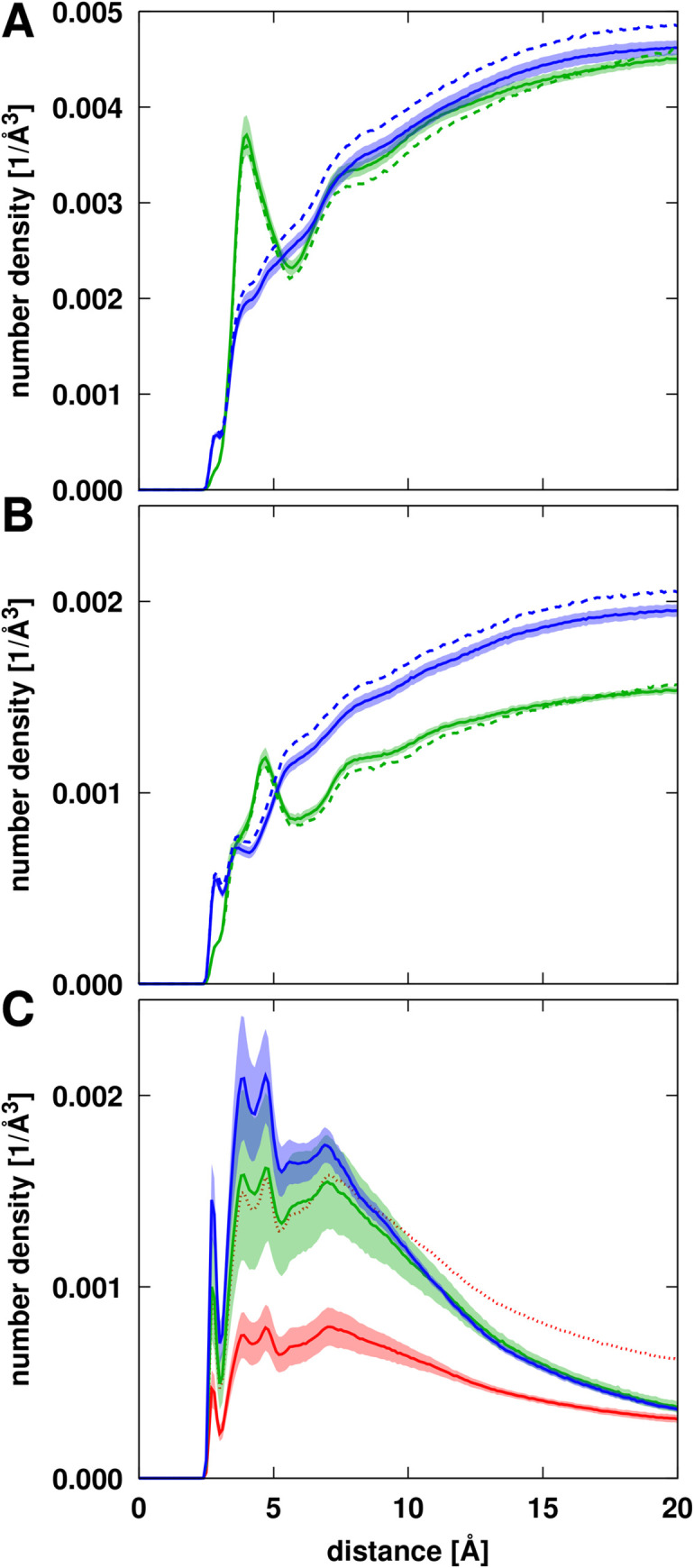
Radial distributions of crowder and substrate atoms around NS3 from simulations with PEG (green) or Ficoll (blue) crowders or in the absence of crowders (red). Distributions are shown for crowder heavy atoms (A), crowder oxygen atoms (B), and for substrate heavy atoms (C). Dashed lines in A and B indicate crowder distributions in the presence of substrates. Distances from the crowder or substrate atoms to the closest NS3 atom were counted and normalized by the total available volume at a given distance from the NS3 surface. For the simulations with only substrates (C), the distribution functions were scaled by a factor of 2 to account for the difference in substrate molarity between the systems without and with crowders (see **S1 Table**). The dotted line shows the results obtained without scaling. Distribution functions were averaged over all trajectories for the systems with the crowders but without substrates. The shaded areas indicate standard errors.

The PEG and Ficoll crowders are present at high concentrations. Based on radial distribution functions (**Fig 4**), the crowders overall do not have a strong preference for interactions with NS3 since crowder atom densities are higher away from the surface of NS3 than near it. As described previously [35], a peak at 4 Å for NS3-PEG interactions indicates specific interactions when PEG is near NS3. This is largely due to interactions involving PEG’s oxygen atoms (**Fig 4B**). Ficoll mostly lacks distinct features in the RDF, although the number of oxygen atoms (in hydroxyls) that could be involved in interactions with the protein is larger in Ficoll than in PEG (**Fig 4B**).

To quantify NS3-crowder interactions, we calculated crowder contacts per amino acid residue as well as the average number of crowders in contact with NS3 (**Table 3**). Perhaps surprisingly, there were more than five PEGs and more than three Ficoll molecules in contact with NS3 at any given time. However, this is a consequence of the dense molecular environment with more than 100 crowder molecules (**S1 Table**). Interestingly, the number of PEGs in contact slightly decreases and the number of Ficolls in contact increases when the substrate is added. Crowder contacts per residue, about 0.2–0.3, tell a similar story (**Table 3**). In addition to simply counting crowder contacts, we also specifically analyzed hydrogen bonding interactions as those are likely to be stronger and potentially longer-lasting contacts. The number of NS3-crowder hydrogen bonds are a small fraction of the total number of contacts, but interestingly, Ficoll formed significantly more hydrogen bonds with NS3 than PEG, even though PEG overall interacted more strongly with NS3 based on the radial distribution functions and total number of contacts. This may be explained by the fact that PEG only has ether oxygens acting as hydrogen bond acceptors, whereas Ficoll has more oxygens and its hydroxyl groups can act both as hydrogen bond acceptors and donors.

**Fig 5:**
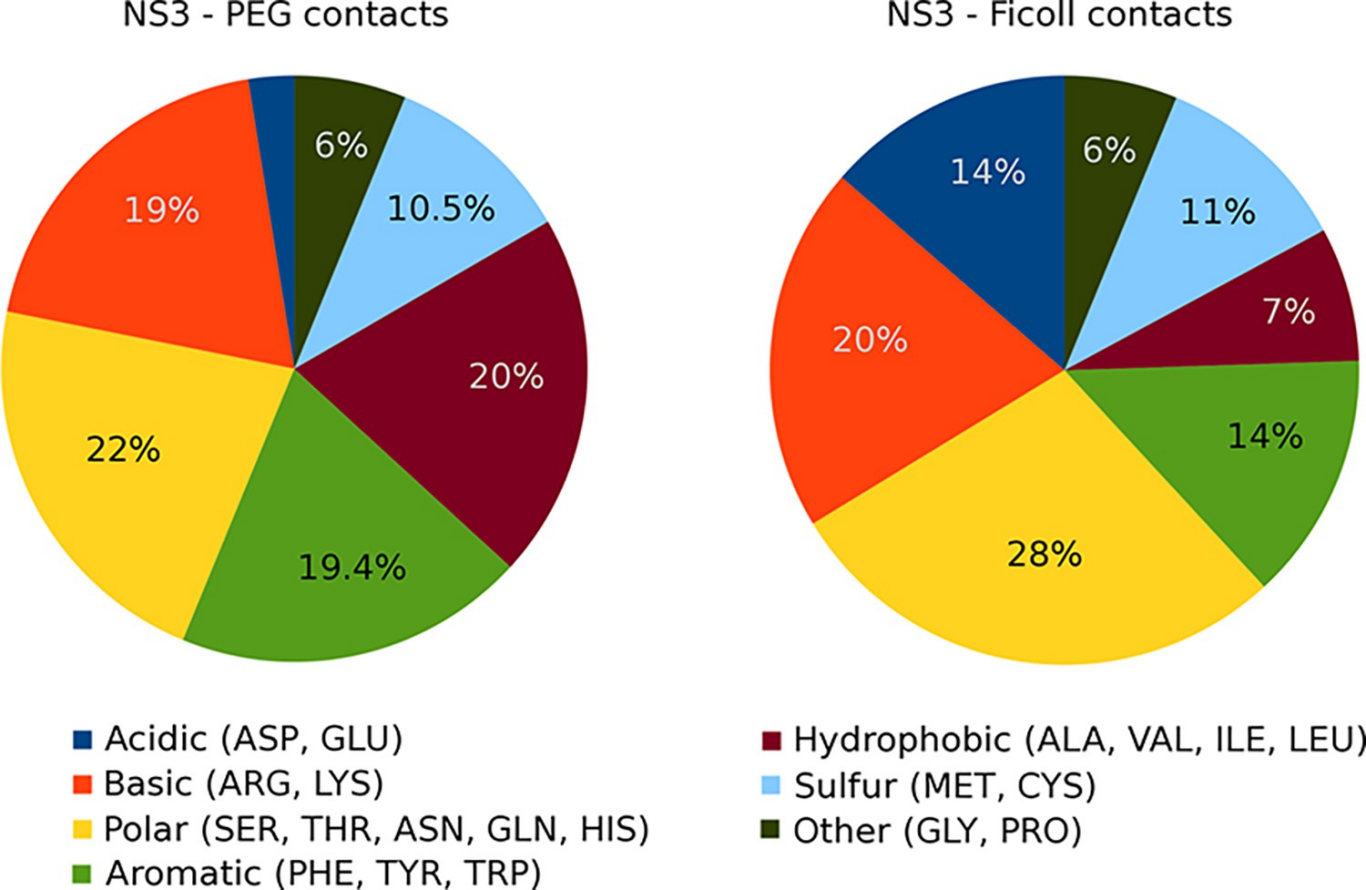
Relative distribution of contacts between PEG and Ficoll crowders and NS3 according to the NS3 amino acids that crowders are in contact with. A contact is defined based on a minimum distance between a crowder oxygen and the closest NS3 heavy atom of less than 5 Å.

**Fig 6:**
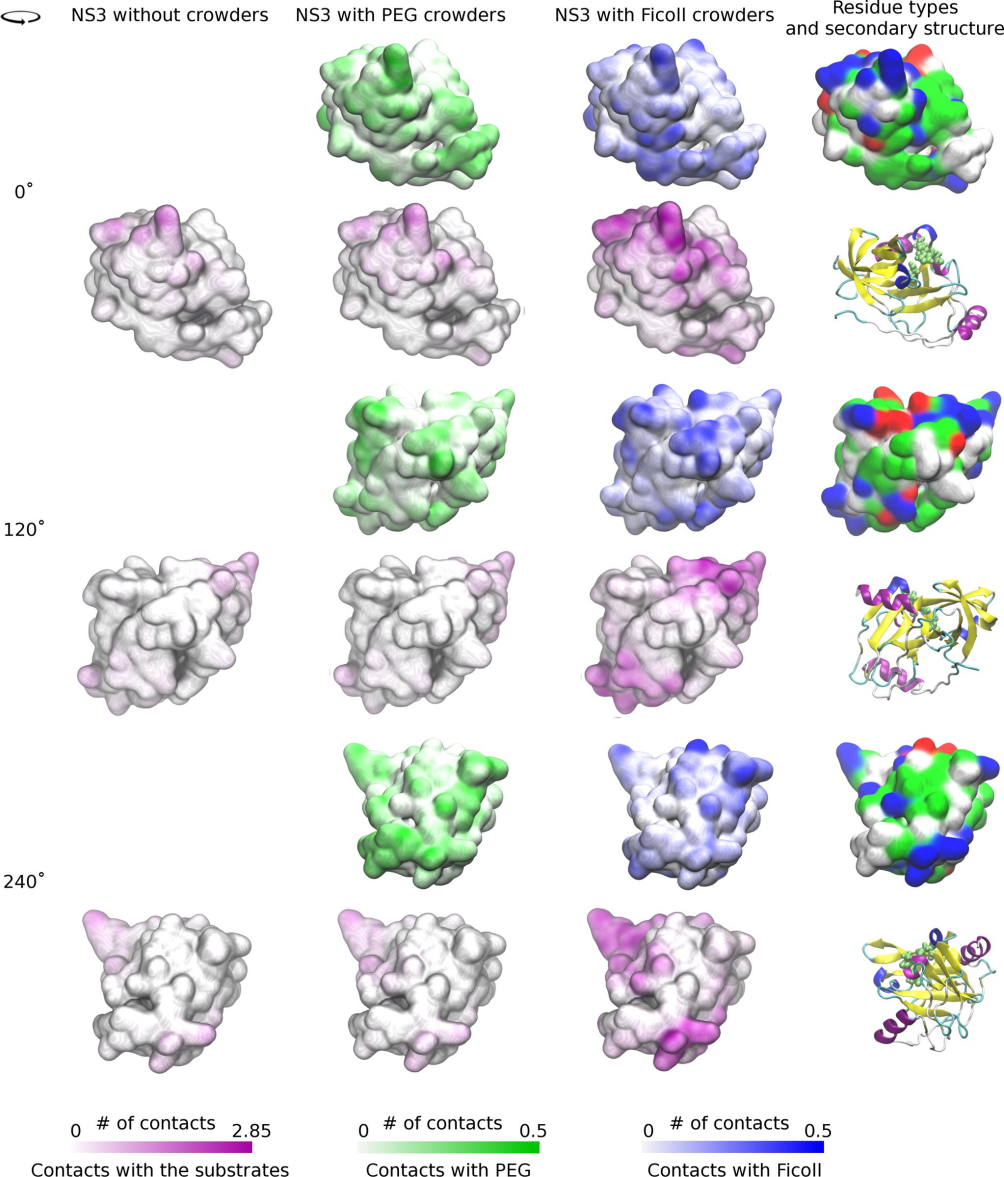
Surface representation of NS3 colored by frequency of crowder and substrate contacts per residue. Different surface colors indicate interactions with PEG (green), Ficoll (blue), and substrates (purple). Different views were generated by rotating the NS3 structure as indicated in the figure. A cartoon representation of NS3 in the same orientations as the surfaces, colored according to secondary structure elements and with van der Waals sphere representation of the catalytic residues, is shown for reference. For clarity, higher number of contacts up to a maximum value of 0.5 contacts per residue are shown at the fully saturated color levels for PEG and Ficoll contacts.

To further compare NS3-crowder interactions between PEG and Ficoll, we broke down interactions according to NS3 amino acid types (**Fig 5**). Both crowders interacted with all types of amino acids, with about half of the contacts with charged or polar residues. However, there are significant differences between PEG and Ficoll with respect to interactions with acidic residues (Ficoll interacts more strongly) and the classic hydrophobic residues (PEG interacts more strongly). When projecting crowder interactions onto the NS3 surface (**Fig 6**), we find broad coverage across most of the surface, but with preferences for certain parts of the structure. As may be expected, both crowders interacted more with the parts of the NS3 structure that are protruding most from the overall global shape, such as some of the solvent-exposed β-turns. In more detail, there are differences between the parts of NS3 in contact with PEG or Ficoll, presumably reflecting the differences in amino acid preferences. Many of the regions in frequent contact with the crowders correspond to the parts of the NS3 structure, where the presence of the crowders alters conformational fluctuations. This leads to the conclusion that crowder contacts directly modulate NS3 conformational dynamics where frequent contacts are formed.

Finally, NS3-crowder contact life times were analyzed based on double-exponential fits to contact survival correlation functions (see Methods). Again, double-exponentials fit the data significantly better than single-exponential fits (**S6 Table**). We find that contacts persisted on two life times with roughly equal weight (**S6 Table**). Short-lived contacts lasted for about 0.2–0.3 ns, with no statistically significant differences between PEG and Ficoll. Longer contacts lasted for about 6.5 ns with PEG and, significantly shorter, for 4.5 ns, with Ficoll. Contacts on different time scales likely reflect different types of contacts with different kinetic barriers to dissociation once contacts are formed. The observation that NS3 forms frequent contacts with multiple crowders and lasting on time scales relevant for diffusion corresponds to the overall slow-down in diffusion of NS3 in the presence of the crowders described above.

The overall picture from the analysis so far is that crowders do not favor interactions with the enzyme, but interactions lasting as long as several nanoseconds occur nevertheless simply due to the highly concentrated environment. Several crowders are in contact with NS3 at any given time and PEG interactions are slightly stronger, with more specific interactions and longer contact life times compared to Ficoll. However, Ficoll forms more hydrogen bonding interactions with NS3 than PEG and has a stronger preference for acidic residues than PEG. The different amino acid preferences lead to different surface interaction patterns explaining different effects of PEG and Ficoll on the internal dynamics of NS3. Further consequences on substrate binding and enzyme function will be discussed in the following.

**Table 3:** Average number of contacts and hydrogen bonds between NS3, substrates, and crowders.

	Contacts / NS3 residue	H-bonds / NS3 residue	Interacting Molecules/ NS3	Contacts / substrate residue	Interacting Molecules/ Substrate
**PEG**	0.287 *(0*.*015)*	0.021 *(0*.*03)*	5.37 *(0*.*22)*		
**PEG w/substrate**	0.279 *(0*.*025)*	0.020 *(0*.*004)*	5.10 *(0*.*33)*	0.298 *(0*.*135)*	0.92 *(0*.*04)*
**Ficoll**	0.201 *(0*.*010)*	0.032 *(0*.*005)*	3.49 *(0*.*09)*		
**Ficoll w/substrate**	0.213 *(0*.*009)*	0.048 *(0*.*018)*	3.73 *(0*.*06)*	0.949 *(0*.*015)*	1.00 *(0*.*01)*
**Substrate**	0.158 *(0*.*026)*	0.046 *(0*.*022)*	2.22 *(0*.*27)*		0.13 *(0*.*03)*
**Substrate w/PEG**	0.167 *(0*.*045)*	0.050 *(0*.*025)*	2.16 *(0*.*35)*		0.10 *(0*.*03)*
**Substrate w/Ficoll**	0.215 *(0*.*027)*	0.071 *(0*.*021)*	2.43 *(0*.*14)*		0.09 *(0*.*01)*

Contacts are defined by a minimum distance of less than 5 Å. Standard errors of the mean from variations between replicate trajectories are given in parentheses.

### Interaction of substrates with NS3 and crowders

Peptide substrates were present in part of the simulations, without and with crowders. This allows us to analyze substrate dynamics and NS3-substrate interactions in the absence and presence of crowders as well as substrate-crowder interactions. In contrast to the crowders, the substrate peptides interact preferentially with NS3/4A (**Fig 4C**). We note that the concentration of substrates in terms of molarity is about two-fold higher in the control simulations without crowders than in the simulations with crowders (**S1 Table**) in order to achieve a comparable number of substrates interacting with NS3/4A with and without crowders. To compare relative preferences of NS3-substrate interactions relative to bulk solvent with and without crowders, the distribution function without crowders was therefore scaled by a factor of two in **Fig 4C**. We find that the presence of crowders increases substrate interactions with NS3/4A and between crowders; Ficoll crowders increase substrate binding more than PEG crowders. On average, there are about two substrates bound to NS3 at any given time (**Table 3**) and substrate contacts per residue are almost the same as crowder contacts per residue. Substrate interactions projected onto the surface of NS3 (**Fig 6**) show strong preferences for certain parts of NS3, mostly near the active site. The substrate is highly acidic and substrate interactions appear to be guided by overall electrostatic attraction to surface patches of NS3/4A with a high concentration of basic amino acids (**Fig 6**).

The substrates themselves sample mostly extended conformations with small percentages of helical structure (**S4 Table**). Crowders do not significantly change the secondary structure sampling of the substrates (**S4 Table** and **S17 Fig**). However, in the presence of the crowders, the substrates appear to be slightly more compact as average radii of gyration are reduced by 1–2% (**S3 Table**).

**Fig 7:**
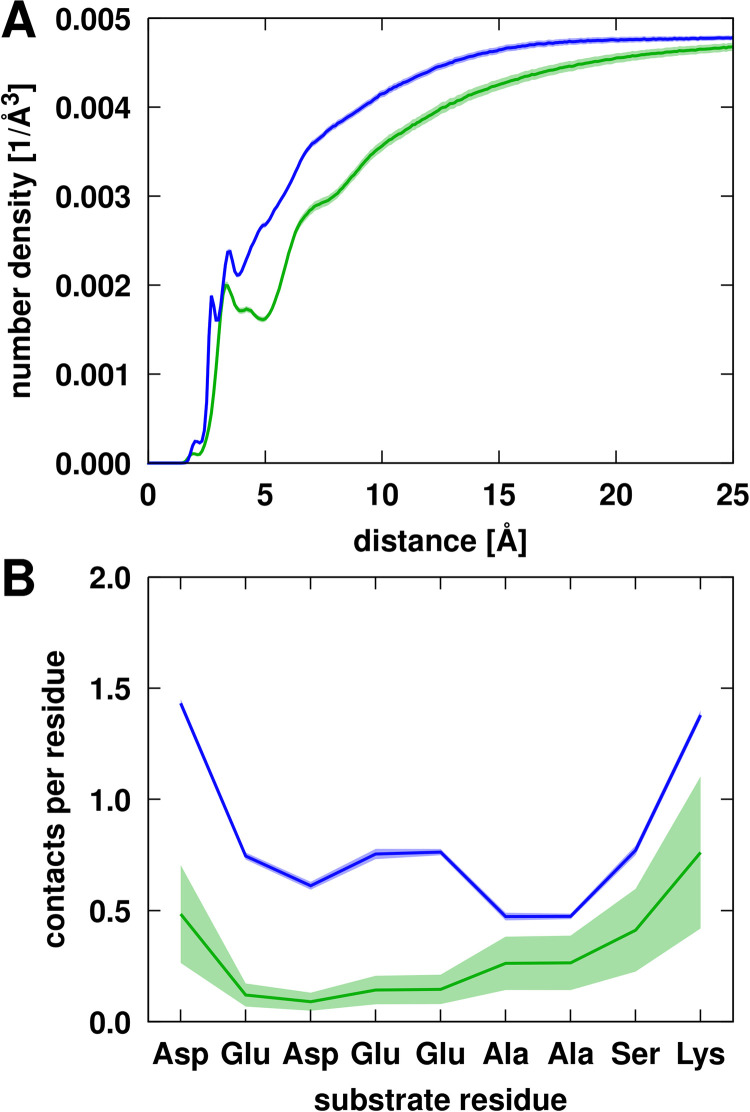
(A) Radial distribution of PEG (green) or Ficoll (blue) crowder heavy atoms around substrates. Distances from the crowder atoms to the closest substrate atom were counted and normalized by the total available volume at a given distance from the NS3 surface. (B) Contacts with PEG (green) or Ficoll (blue) per substrate residue along the substrate sequence. Trajectory averages are shown as solid lines with the shaded areas indicating standard errors.

Substrate contacts with crowders are characterized in **Fig 7**. As for NS3, the peptide substrates do not interact preferentially with the crowders. In contrast to NS3, the substrate interacts more strongly with Ficoll than with PEG. This is a result of Ficoll preferring interactions with acidic amino acids (**Fig 7B**) relative to PEG. As a result, there is, on average, almost 1 contact with Ficoll per substrate residue, but only 0.3 contacts per residue with PEG (**Table 3**). Nevertheless, the number of crowder molecules in contact anywhere with a given substrate is similar, around one crowder per substrate on average (**Table 3**). Substrate-crowder life times are somewhat shorter than NS3-crowder life times, with short contact times of 0.1 ns and long contact times of around 1.5 ns (**S7 Table**). This suggests a picture where substrates mostly travel attached to a crowder. Substrates detach from crowders and exchange interactions frequently, but the contacts still remain long enough for substrate diffusion to be dominated by crowder diffusion as discussed above.

### Crowding effects on NS3 active site

**Fig 8:**
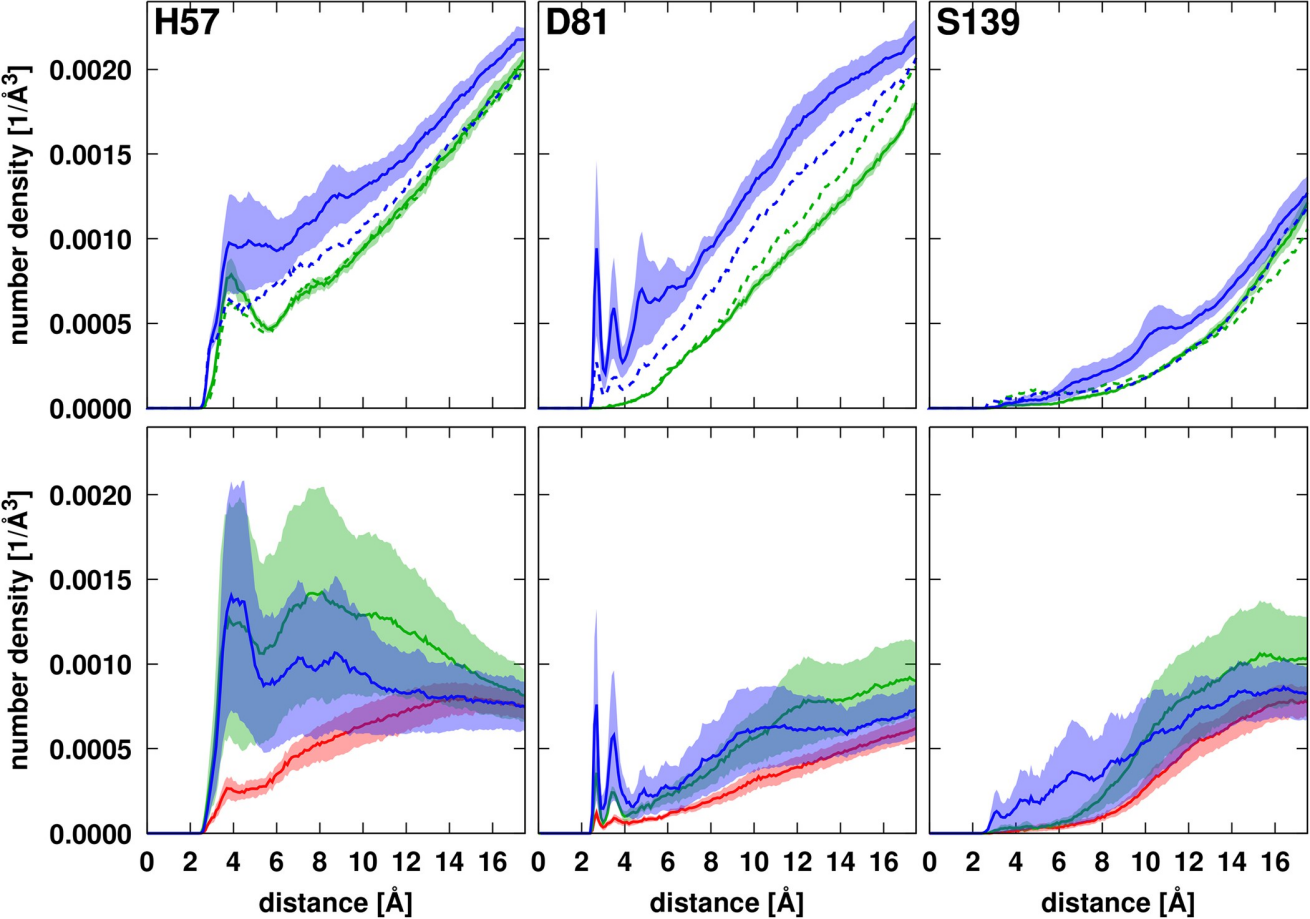
Radial distribution of PEG (green) or Ficoll (blue) crowder heavy atoms (top row) or substrate heavy atoms (bottom row) in simulations with PEG (green), Ficoll (blue) or without crowders (red) around NS3 active site residues. Distances from the crowder atoms to the closest substrate atom were counted and normalized by the total available volume at a given distance from the given NS3 residues. The distribution function of substrates in the simulations without crowders are not scaled; note that substrates are present at twice the overall concentration in those systems. Dashed lines in the top row panels indicate crowder distributions in simulations with substrate. Trajectory averages are shown as lines with the shaded areas indicating standard errors.

We now focus on the specific effects near the NS3 active site with the ultimate goal of explaining the experimental data that showed enhancement of NS3/4A activity in the presence of Ficoll crowders but reduced activity with PEG crowders [15]. According to radial distribution functions, the crowders themselves do not interact strongly with the active site residues, although Ficoll interacts more than PEG (**Fig 8**). At 10 Å distance, crowder atom densities range from less than 0.0005 Å^-3^ near S139 to 0.0012 Å^-3^ near H57 which is much less than the average crowder density of 0.0035 Å^-3^ with respect to any residue of NS3 (**Fig 4**). This may be expected since the active site residues lie at the bottom of a surface pocket (**Fig 1A–1C**) where non-specific binding with the crowders is less likely.

Substrate molecules are expected to bind near the active site for cleavage, but, without crowders, substrate atom densities near the active site are similar to crowder atom densities (**Fig 8**). However, both crowders significantly enhance the presence of substrates near the active site, especially near H57. At the same time, Ficoll crowder densities near the active site are reduced when substrate is present.

The geometry of the active site residues is important for enzyme function. D81 is expected to coordinate with H57, and H57 would attach the hydroxyl of S139 in the presence of substrate in the first step of the peptide cleavage reaction [69]. Therefore, we analyzed whether the presence of a crowder and/or a substrate at close distance to the active site would affect the conformational sampling and interactions between the active site residues.

**Fig 9:**
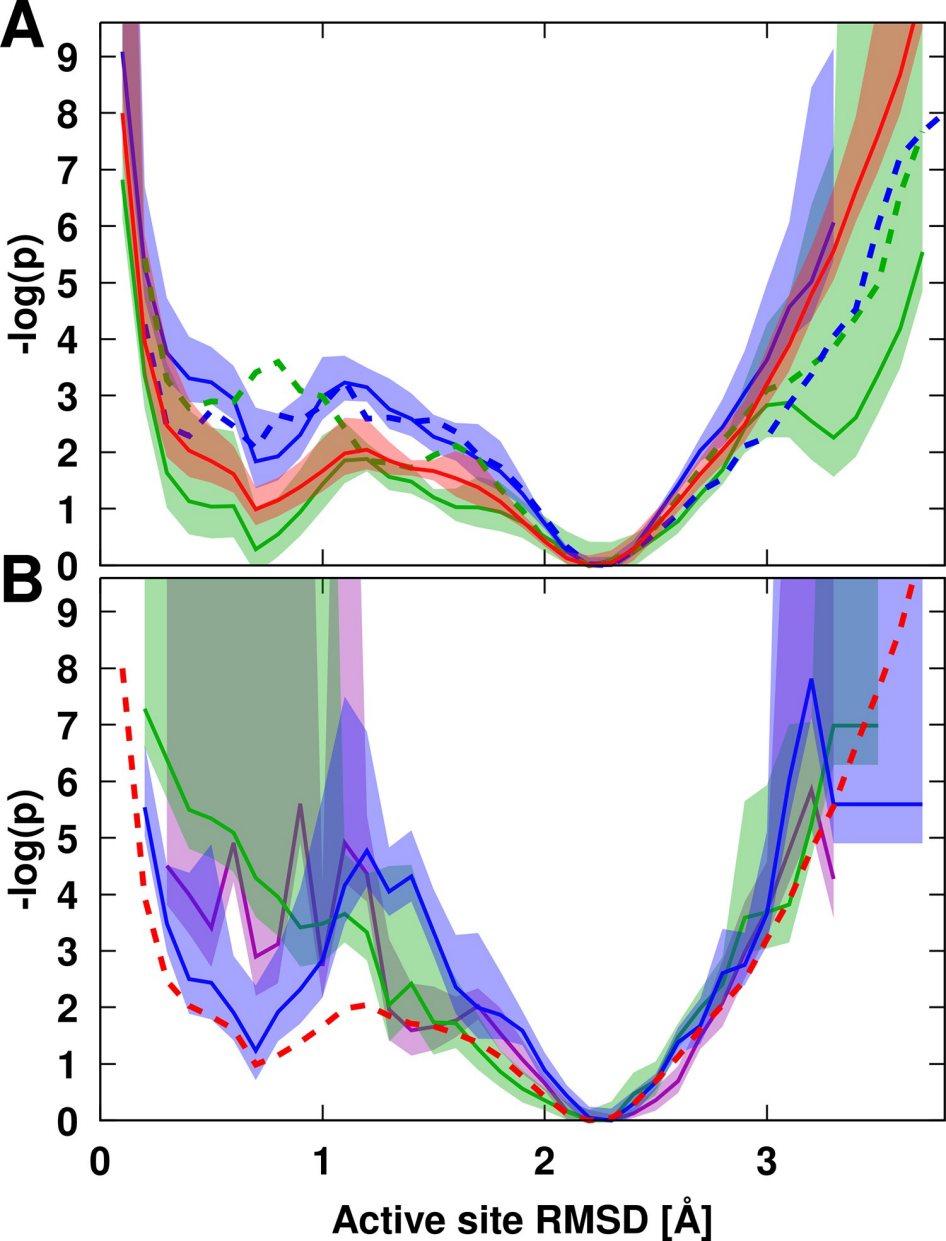
Relative probabilities of sampling different values of root mean-square deviations (RMSD) of heavy atoms of active site residues (57, 81, 139). RMSD values were calculated with respect to experimental reference structure (PDB ID: 4JMY) after optimal superposition of the respective residues. Relative probabilities were shifted so that the most likely distance corresponds to a log(p) value of 0. Results are shown for simulations in water (red), with only substrates (purple), and in the presence of PEG (green) or Ficoll (blue) crowders. The top panel (A) compares distributions in water with distributions when crowders are in contact (<5 Å). Solid lines show results without substrate, dashed lines show results when substrates are present along with the crowders. In the bottom panel (B), distributions are shown for cases where the substrate is in contact (<3 Å). For comparison, the dashed line shows the distribution in water. Trajectory averages are shown as lines with the shaded areas indicating standard errors.

We used RMSD of the active site residues (57, 81, 139) to compare the overall geometry of the active site relative to the crystal structure during the simulations. Probabilities of finding different RMSD values are shown in **Fig 9**. As the structure of the enzyme fluctuates, there is a distribution of conformations ranging from structures very similar to the crystal structure (<1 Å) that are likely reflecting a conformation close to the catalytically competent state to structures that deviate more significantly (up to about 3 Å). The most likely structures seen in the simulations deviate by about 2.2 Å. When crowders are present, close contacts of PEG increase the sampling of crystal-like structures, whereas close Ficoll contacts actually make the sampling of such conformations less likely (**Fig 9A**). When substrates are present as well, neither crowder enhances the sampling of crystal-like conformations when coming in contact with the active site (**Fig 9A**). The functionally more relevant scenario involves cases where substrate molecules approach the active site. Without crowders, crystal-like structures are not sampled as frequently as in pure water when substrate molecules come close to the active site (**Fig 9B**). The additional presence of PEG does not change that. However, the addition of Ficoll restores the more frequent sampling of crystal-like active site conformations when substrate approaches the active site seen in pure water (**Fig 9B**). This suggests that PEG and Ficoll crowders act differently in promoting catalytically relevant active site geometries along with substrate binding.

To analyze the effect of crowders and substrate on the NS3 active site in more detail, we calculated the distribution of side chain χ_1_ torsion angles and inter-residue distances between active site residues. χ_1_ torsion angles as a function of the closest crowder or substrate distance from the active site are shown in **S18–S20 Figs**. In addition, we compare the distribution of χ_1_ angles between dilute solvent without crowders or substrates with the distributions when a PEG, Ficoll, or substrate molecule is within 5 Å of any of the active site residues (**S21 Fig**). The sampling of side chain orientations is broad, covering different rotameric states and not just the conformation found in the crystal structure (PDB ID: 4JMY) (**S21 Fig**). This may be expected since the residues are solvent-exposed and the crystallographic conformation may be further constrained by the presence of a tightly bound inhibitory peptide. When crowders interact without substrates, the side chain orientations appear to change somewhat, for example the rotameric state with χ_1_ = 60° for H57 seen also in the crystal structure became less populated when Ficoll crowders interacted but more populated when PEG crowders were close. However, most of these effects disappear when substrates are present along with the crowders (**S21 Fig**). On the other hand, close substrate contacts themselves also appear to modulate the torsional sampling, especially when a substrate is in very close direct contact (<3 Å). More specifically, χ_1_ = 60° for H57 and χ_1_ = -150° for D81 become preferred states similar to the crystal structures when substrate is in very close contact (<3Å) in the presence of Ficoll (**S20 Fig**, lower panels). For S139, close substrate contacts promote non-crystal values of χ_1_ = -150° more in the presence of Ficoll than in the presence of PEG, although the crystal-like configuration with χ_1_ = -60° remains the preferred state with both crowders (**S20** and **S21 Figs**).

We further analyzed side chain center distances between active site residues. As the side chain orientations fluctuated, side chain distances also displayed significant dynamics between close contacts of 4 Å to larger distances of 11 Å with broad sampling around preferred distances depending on residue pair (**S22–S24 Figs**). The results are summarized for close crowder contacts without substrates in **S25 Fig**. We find that with PEG crowders near the active site, close distances between all active site residues were most likely 4.5 Å for H57-D81, 5 Å for H57-S139, and 8 Å for D81-S139 (**S25 Fig**). In contrast, Ficoll near the active site led to the opposite effect of active site residues moving further away from each other as the most likely distances increased to 8 Å for H57-D81, 7.5 Å for H57-S139, and 9.5 Å for D81-S139 (**S25 Fig**). Without crowders the distance distributions were found to be intermediate between the distributions obtained with PEG and Ficoll (**S25 Fig**). However, as for the torsion angle sample, these effects mostly disappeared when substrates were present along with the crowders since distance distributions with or without crowders became largely indistinguishable when considering the statistical uncertainties (**S25 Fig**). Close substrate contacts by themselves were also correlated with shorter distances becoming more probable when PEG was present (**S25 Fig**). With Ficoll, the same effect was observed, but required very close substrate distances to the active site (<3 Å, **S24 Fig**). These findings suggest that crowder-induced close substrate interactions may facilitate an induced-fit mechanism of substrate binding in the active site, especially with Ficoll crowders.

We proceeded to look at two key atomistic distances, H57:Nδ-H … Oδ1/2-D81 and H57-Nε … HOγ-S139, in more detail (**S26–S28 Figs**). Again, there are significant fluctuations of these distances ranging from hydrogen-bond distances of 2 Å to values beyond 10 Å as a reflection of significant dynamics (**S26–S28 Figs**). These distances need to be close for the cleavage reaction to proceed. Comparing distance distributions again for the case where crowders or substrates are in close contact with the active site (**S29 Fig**), we find similar conclusions as for the side chain distances, namely PEG by itself promotes shorter distances whereas Ficoll by itself increases these catalytically important distances. Substrate contacts by themselves without crowders do not strongly promote closer distances since hydrogen-bond distances of less than <3Å are observed much less frequently than longer distances (**S29 Fig**, bottom panels). However, close H57-Nε … HOγ-S139 hydrogen bond distances, necessary to start the cleavage reaction, become relatively more favorable than intermediate distances of 4–6 Å when Ficoll crowders are present (**S28 and S29 Figs**, right lower panel). In fact, with PEG crowders and substrate, sampling of close distances (<2.5 Å) of this hydrogen bond almost vanishes (**S28 Fig**), whereas Ficoll enhances this interaction when a substrate is close to the active site.

To complement the conformational sampling analysis of active site geometries, we also characterized side chain fluctuations from orientational correlation function decays (**S30 Fig**). Double-exponential fits to the correlation functions (**S8 Table**) reveal fast motions on time scales of around 1 ns and slow motions on time scales between about 50 and 500 ns. The slower time scales presumably correspond to transitions between different rotamer states. We find that the presence of Ficoll crowders appears to accelerate the slow motions over dilute solvent dynamics by 20–50% when substrates are not present (**S8 Table**), whereas PEG crowders may have slowed down those motions for H57 (by 10%) and S139 (by 11%) while increasing the slow motion for D81 (by 40%) (**S8 Table**). However, these variations may lie within the uncertainties of our analysis given the estimated errors of the correlation functions (**S30 Fig**). The presence of substrates by themselves, accelerated the side chain motions significantly, about twofold (**S8 Table**). When both crowders and substrates are present, faster motions were retained for H57, but mostly slowed down again for D81 and S139 with varying effects depending on the crowder type and residue (**S8 Table**) that may be difficult to distinguish with certainty given our data. This analysis may suggest that the crowders can influence the dynamics of functionally relevant residues to different degrees with possible consequences for enzyme catalysis. However, much longer simulations are likely needed to come to firmer conclusions.

### Crowding effects on substrate binding modes

**Fig 10:**
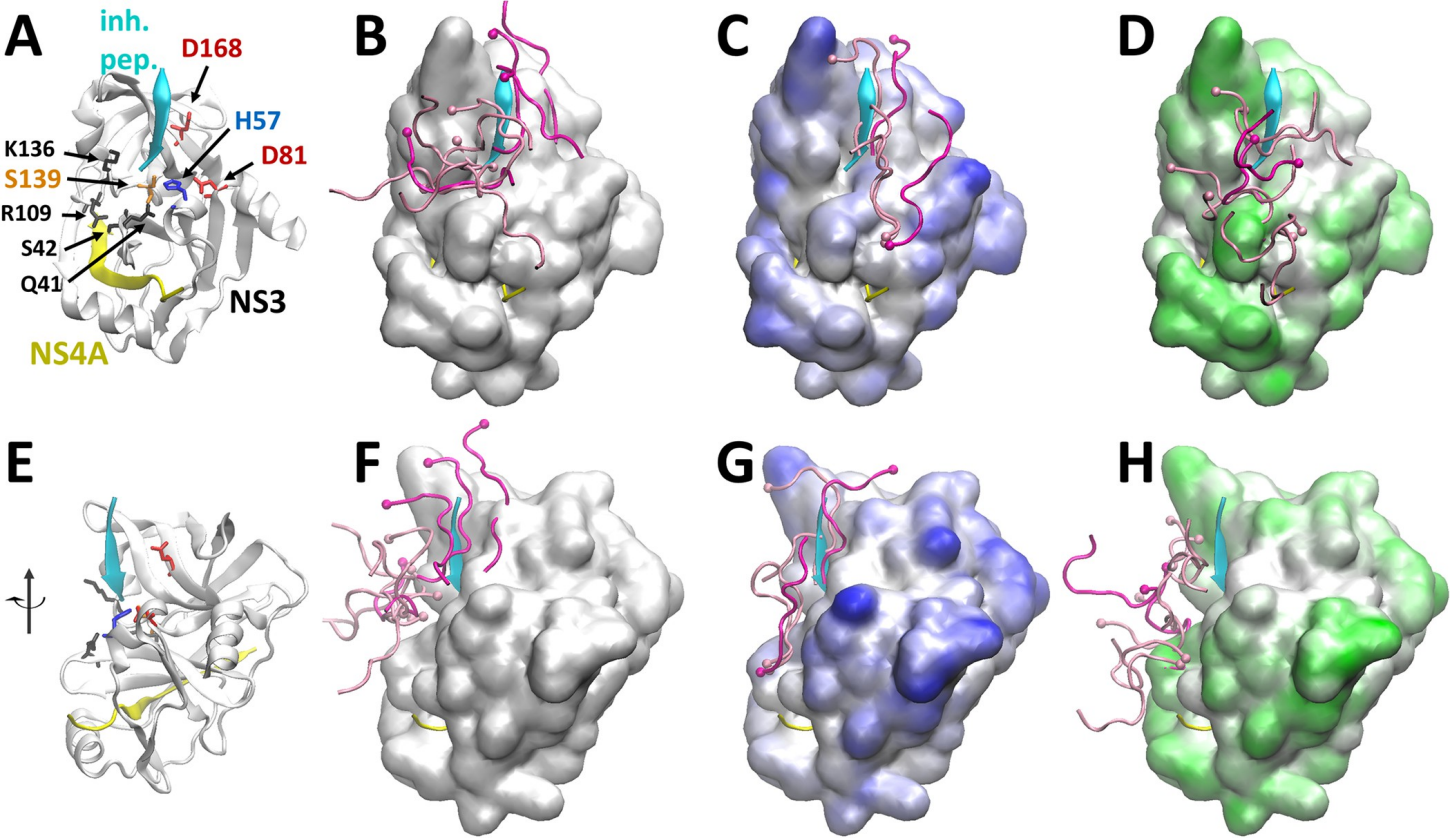
A cartoon representation of NS3/4A is shown for reference with labels identifying key residues in the active site and those involved in substrate binding (A). Substrate binding near NS3 active site from simulations is indicated without crowders (B), with Ficoll crowders (C) and with PEG crowders (D). Substrate conformations shown are representative structures closest to cluster centers for the largest clusters covering the top 25% (dark magenta) and the next 25% (light magenta) of all substrate conformations extracted for a given system. Surface representations of NS3 are colored based on crowder contact frequencies, with Ficoll (C, blue) and with PEG (D, green). The bottom row of figures (E-H) shows the representations in A-D after rotation to the left by about 45°.

Finally, we looked at the binding modes of substrates near the active site. Experimental structures of NS3/4A provide partial evidence of where the substrate is expected to bind NS3, mainly based on the binding mode of inhibitory peptides [70,71]. According to these studies, the substrate binds in a mostly extended conformation with the N-terminal part of the substrate interacting with D168 of NS3 and pointing towards the NS3/4A active site (comprised of H57, D81, and S139). We analyzed the preferred binding modes from the simulations based on clustering analysis of substrates found near the active site (**Fig 10** and **S9–S10 Tables).** Substrates interacted in a variety of ways. Only some conformations were close to the exact conformation of the inhibitory peptide bound in the crystal structure (PDB ID: 4JMY). Without crowders, we find that bound substrates were generally located in the surface depressions above and to the left of the active site in **Fig 10B**, interacting with key residues known from previous studies to be involved in substrate binding (such as Asp168 at the N-terminal side of the substrate and Gln41, Ser42, Arg109, and Lys136 for the C-terminal part of the substrate near the cleavage site [71,72]). However, some of the bound conformations appeared to be relatively far from the surface (**Fig 10F**). In the presence of Ficoll crowders, substrate binding appeared to become more focused towards the inhibitory peptide binding site in the crystal structure, and mostly involved linearly extended substrates across the active site (**Fig 10C–10G**). In contrast, the presence of PEG appeared to have an opposite effect of more diffuse binding of the substrate pointing in different directions but without clear examples of the substrate following the binding mode along the cleft above the active site suggested by the inhibitory peptide bound in the crystal structure. Additional quantitative analysis of individual clusters focused on end-to-end distances of substrates to measure their compactness, alignment of substrates with respect to the substrate fragment in the 4JMY structure to assess how similar the substrate orientation was to the presumed binding configuration, and the question to what degree different substrates contributed to a given cluster and how long substrates remained in contact near the active site (**S9–S11 Tables**). Accordingly, we found that substrates interacting near the active site are mostly compact in the presence of PEG crowders (**S10**) whereas substrates are significantly mostly extended in the most populated clusters in the presence of Ficoll crowders (**S11 Table**). At the same time, substrates in the most populated clusters in the presence of PEG are oriented mostly orthogonal to the substrate fragment present in 4JMY, as indicated by near-zero cosθ values (**S10 Table**). In contrast, substrates in the presence of Ficoll were highly aligned with the substrate fragment (**S11 Table**), although in some cases the direction of the peptide was opposite as indicated by negative cosθ values. Many of the most populated clusters involve predominantly a single substrate remaining in contact near the active site for longer times (up to 10 ns). However, different clusters capture different substrate interactions with similar features suggesting exchange of substrates and that the findings are not dominated by a single substrate remaining bound near the active site for a very long time.

Along with the differences in substrate binding modes, **Fig 10** also shows the preferences of crowder molecules to interact with NS3 residues. It can be seen that Ficoll interacts more prominently on the right side of the active site (near D81 and above), whereas PEG crowders interact much less in that area, presumably because of Ficoll’s preference for acidic residues. Based on this analysis, we speculate that Ficoll binding to NS3 guides substrates to more easily find the catalytically relevant binding mode whereas PEG binding to NS3 may actually have the opposite effect of discouraging catalytically competent substrate binding.

## Conclusions

Understanding enzyme function in the presence of crowders is important for understanding enzyme function *in vivo*. Proteins are the main type of crowder molecules encountered in biology but experiments often use artificial crowders to focus on generic crowder properties and avoid the complexity of biological environments. The present study was motivated by the observation that the enzyme kinetics of NS3/4A responded very differently to two different types of artificial crowders, PEG and Ficoll. Although K_M_ values in the absence and presence of crowders were found to be similar, catalytic rates decreased with PEG and significantly increased with Ficoll compared to the enzyme in buffer solution [15]. This suggested that these commonly used crowders may affect enzyme function via crowder-specific interactions rather than the more generic volume exclusion effect typically assumed to be dominant for these types of crowders [1].

The extensive simulations of NS3/4A in the presence of PEG and Ficoll as well as peptide substrates that are presented here offer molecular-level insights into how PEG and Ficoll may interact differently with NS3 and the substrates, and how they may lead to different enzyme behavior. As expected, neither PEG and Ficoll interact strongly with NS3. However, interactions do occur with enough frequency for NS3 to be in contact with several crowder molecules at any given time. Moreover, NS3-crowder interactions are relatively long-lived, up to several nanoseconds. An interesting finding is that PEG and Ficoll have different preferences for different amino acid types. Ficoll clearly prefers interactions with acidic residues, whereas PEG is more likely to interact with amino acids that have hydrophobic aliphatic side chains. As a result, crowders decorate different parts of the NS3 surface according to their amino acid preferences.

The crowders also interact with the substrate peptides, especially Ficoll because the substrates are highly acidic. In fact, based on the analysis presented here, substrates rarely diffuse on their own but are in contact with a crowder at any given time. This suggests that substrate diffusion is essentially slaved to crowder diffusion. The crowders considered here were relatively small, and therefore the effective substrate diffusion was reduced more moderately in the presence of crowders, but one expects this effect to be significantly more pronounced when much larger crowders such as PEG 6000 or Ficoll 400 are used as common in experiments. In that case, reduced substrate diffusion could become a rate-limiting step for enzyme reactions. On the other hand, we found that the presence of crowders enhanced substrate binding to NS3, perhaps by delivering substrates to NS3 and then hindering substrates from leaving once bound to NS3. Moreover, crowders appeared to modulate the substrate binding modes, essentially by binding to certain NS3 surface regions thereby focusing substrate binding on the remaining patches that are unoccupied by crowders. With PEG crowders this led to more diffuse binding, while Ficoll may focus substrate binding towards a catalytically competent pose. In addition, Ficoll crowders also appeared to facilitate the formation of catalysis-relevant conformations of active site residue when a substrate molecule was closely interacting. Taken together, this may explain the experimental observation of increased NS3/4A activity in the presence of Ficoll compared to buffer and when PEG is present.

While the study highlights specific effects of PEG and Ficoll due to different interactions with proteins and peptides at the molecular level, it does not fully interpret the experimental data. Substrate binding near the active site was observed, but it is unclear whether any of the bound substrates fully reached a catalytically competent binding pose. Probably much more extensive simulations with enhanced sampling techniques could overcome this challenge. However, it is also not clear from experiment how exactly cleavable substrates would bind to the active enzyme. Another limitation is that the crowder models used here may compare to PEG 600 or 2000, but not the much larger PEG 6000 and Ficoll 400 crowders used in the experiments. Simulations with much larger crowders would be computationally prohibitive. Another complication is that the higher-order structure of Ficoll is not well-characterized. Finally, the simulations presented here only address classical aspects of biomolecular interactions and dynamics but do not directly describe the peptide cleavage reaction, which requires quantum-mechanical treatments [69]. Studies of enzyme catalysis via QM/MM calculations where crowder molecules are considered explicitly as part of the studied system would be interesting future work.

While the work here focuses on the specifics of the NS3/4A protease and compares the effects of two types of artificial crowders, the more general conclusions are that even artificial crowders may significantly impact functional studies under crowding conditions as a result of molecule-specific interactions with proteins. We hope that the insights gained here will motivate further studies of enzymes under crowded conditions and guide the interpretation of results obtained with different crowding agents.

## Supporting information

S1 Text Supplementary Methods. (PDF)

S1 Fig Chemical structure of the polysucrose molecule used as a model for Ficoll. The sucrose molecules are connected with glycerol linkers. Carbon atoms are annotated according to the numbers used in the CHARMM force field.
(TIF)

S2 Fig Building blocks used to parameterize Ficoll molecules. Glucose and fructose are shown, together forming sucrose molecules. All structures are shown along with atom names used in the CHARMM topology.
(TIF)

S3 Fig Structures of isomaltulose and melezitose and CHARMM force field patches. The patches are used to form these molecules from glucose and fructose monomers. The 2C6 and 2C3 atoms used to parameterize carbon atoms in the model of Ficoll are marked with orange circles.
(TIF)

S4 Fig Time evolution of coordinate root mean square deviations of NS3/4A. RMSD values are shown in water (W), in the presence of PEG (P) and Ficoll (F) and with substrates (S, PS, FS). RMSD values are based on Cα coordinates after optimal superposition with respect to the experimental structure (PDB ID: 4JMY) for NS3 and the central 13 residues of NS4A for which structure information is available in the PDB. Different colors distinguish individual trajectories.
(TIF)

S5 Fig Time-averaged Cα root mean square deviations as a function of simulation time. Results are shown for NS3/4A in water (red), in the presence of PEG (green) or Ficoll (blue). Solid and dashed lines are from simulations in the absence and presence of substrates, respectively. Averages were calculated over 20 ns trajectory segments. Error bars indicate the standard errors of the mean from variations between replicate simulations.
(TIF)

S6 Fig Radius of gyration distributions of NS3 and NS4A. Results are shown for NS3 (A,B) and NS4A (C,D) without (A,C) and with (B,D) substrates in water (red) or in the presence of PEG (green) or Ficoll (blue) crowders. Individual histograms were averaged over replicate trajectories. Error bars indicate standard errors for each bin.
(TIF)

S7 Fig Root mean square fluctuations of NS3. Results are shown without (A) and with (B) substrates in water (red) or in the presence of PEG (green) or Ficoll (blue) crowders. RMSF was calculated from Cα atoms with respect to the average structures of NS3 for each trajectory after superposition onto a reference structure. Solid lines reflect trajectory averages, shaded areas indicate standard errors of the mean.
(TIF)

S8 Fig Root mean square fluctuations of NS3 in non-crowded and crowded environments projected onto the protease structure. RMSF values were calculated for the C_⍺_ atoms of each amino acid, with respect to the average structure for each trajectory, after superposition onto the crystallographic structure (PDB ID: 4JMY). For clarity, in all cases the higher end of the RMSF scale (dark colors) in the legends correspond to RMSF values between 2.0 and the maximum value of 5.23 Å.
(TIF)

S9 Fig Root mean square fluctuations of NS4A. Results are shown without (A) and with (B) substrates in water (red) or in the presence of PEG (green) or Ficoll (blue) crowders. RMSF was calculated from Cα atoms with respect to the average structures of NS4A for each trajectory after superposition of the ordered central residues (21–31). Solid lines reflect trajectory averages, shaded areas indicate standard errors of the mean.
(TIF)

S10 Fig Secondary structure evolution of the NS4A cofactor in the simulations with water and with either PEG or Ficoll crowders. Helical conformations are marked in pink or blue, β-strands in yellow, and turns in cyan. Each graph shows data from one MD trajectory.
(TIF)

S11 Fig Secondary structure evolution of the NS4A cofactor in the simulations with substrates and with either PEG or Ficoll crowders. Helical conformations are marked in pink or blue, β-strands in yellow, and turns in cyan. Each graph shows data from one MD trajectory.
(TIF)

S12 Fig Ramachandran map of backbone torsion angles φ and ψ for N-terminal residues (residues 2–19) of NS4A. Results are shown from sampling in water (W), in the presence of PEG (P), in the presence of Ficoll (F), with substrates (S), with substrates in the presence of PEG (PS), and with substrates in the presence of Ficoll (FS). Colors indicate probabilities (-log(p)) according to the color bar.
(TIF)

S13 Fig Ramachandran map of backbone torsion angles φ and ψ for C-terminal residues (residues 31–53) of NS4A. Results are shown from sampling in water (W), in the presence of PEG (P), in the presence of Ficoll (F), with substrates (S), with substrates in the presence of PEG (PS), and with substrates in the presence of Ficoll (FS). Colors indicate probabilities (-log(p)) according to the color bar.
(TIF)

S14 Fig Mean square displacement of centers of mass vs. time used for determining translation diffusion coefficients for NS3/4A. Results are shown without substrates (A), with substrates (B), and for substrates (C) in simulations with water (red), in the presence of PEG (green), and in the presence of Ficoll (blue).
(TIF)

S15 Fig Rotational correlation functions used for determining rotational diffusion coefficients for NS3/4A. Results are shown without substrates (A), with substrates (B), and for substrates (C) in simulations with water (red), in the presence of PEG (green), and in the presence of Ficoll (blue).
(TIF)

S16 Fig MSD/t vs. time on a log-log scale based on trajectory averaged mean square displacement curves. The underlying MSD curves are shown in **S14 Fig**. Results are shown for NS3/4A without substrates (A), with substrates (B), and for substrates (C) in simulations with water (red), in the presence of PEG (green), and in the presence of Ficoll (blue). Shaded areas indicate standard errors of the mean.
(TIF)

S17 Fig Ramachandran map of backbone torsion angles φ and ψ for substrates. Results from sampling substrates with NS3/4A in water (S), in the presence of PEG (PS), and in the presence of Ficoll (FS). Colors indicate probabilities (-log(p)) according to the color bar.
(TIF)

S18 Fig Side chain χ_1_ torsion angle sampling of active site residues (H57, D81, S139) as a function of closest PEG crowder distance. The distance was calculated to any of active site residues in simulations without (top row) and with (bottom row) substrates. In the crystal structure (4JMY), the values of the χ_1_ torsion are 76.6° for H57, -161.4° for D81, and -82.6° for S139. Colors indicate probabilities (-log(p)) according to the color bar.
(TIF)

S19 Fig Side chain χ_1_ torsion angle sampling of active site residues (H57, D81, S139) as a function of Ficoll crowder distances. See **S18 Fig** for additional details.
(TIF)

S20 Fig Side chain χ_1_ torsion angle sampling of active site residues (H57, D81, S139) as a function of substrate distances. Results are shown for simulations with only substrate (top row) and with PEG (middle row) or Ficoll (bottom row) crowders as in **S18 Fig**.
(TIF)

S21 Fig Sampling of side chain χ_1_ torsion angles for active site residues (H57, D81, S139) in water and when crowders or substrates are in close contact. Contacts were defined as a minimum distance of 5 Å. Results for simulations with water (red) or only crowders (PEG: green, Ficoll: blue) are shown in the top row. Results for simulations with substrate but based on close crowder contacts are shown in the middle row. Results based on substrates being in close contact are shown in the bottom row. In the bottom row, the results from the simulations with only substrates are shown in purple. The dilute water distributions are shown as a dashed line for reference. Shaded areas indicate standard errors.
(TIF)

S22 Fig Side chain center distances between active site residues (H57, D81, S139) as a function of PEG crowder distances. See **S18 Fig** for further details. In the crystal structure (PDB ID: 4JMY), the distances are 4.45 Å for H57-D81, 4.69 Å for H57-S139, and 7.37 Å for D81-S139.
(TIF)

S23 Fig Side chain center distances between active site residues (H57, D81, S139) as a function of Ficoll crowder distances. See **S18** and **S22 Figs** for further details.
(TIF)

S24 Fig Side chain center distances between active site residues (H57, D81, S139) as a function of substrate distances. Results are shown for simulations with only substrates (top row) and with PEG (middle row) or Ficoll (bottom row) crowders as in **S22 Fig**.
(TIF)

S25 Fig Sampling of side chain center distances between active site residues (H57, D81, S139) in water and when crowders or substrates are in close contact. See **S21 Fig** for further details.
(TIF)

S26 Fig Catalysis-relevant distances between active site residues (H57:Nδ-H … Oδ1/2-D81 and H57-Nε … HOγ-S139) as a function of PEG crowder distances. Results are shown for simulations without (top row) and with (bottom row) substrates. Colors indicate probabilities (-log(p)) according to the color bar.
(TIF)

S27 Fig Catalysis-relevant distances between active site residues (H57:Nδ-H … Oδ1/2-D81 and H57-Nε … HOγ-S139) as a function of Ficoll crowder distances. See details in **S26 Fig**.
(TIF)

S28 Fig Catalysis-relevant distances between active site residues (H57:Nδ-H … Oδ1/2-D81 and H57-Nε … HOγ-S139) as a function of substrate distances. Results are shown for simulations with only substrate (top row) and with PEG (middle row) or Ficoll (bottom row) crowder as in **S26 Fig**.
(TIF)

S29 Fig Sampling of catalytically relevant active site distances in water and when crowders or substrates are in close contact. See **S21 Fig** for further details. The right-most column zooms in on the shorter-distance region of the data shown in the middle column.
(TIF)

S30 Fig Orientational correlation function for active site side chains (H57, D81, S139) in NS3. Fluctuations were calculated based on the Cα-Nε vector (H57, A/B), the Cα-Cγ vector (D81, C/D), and the Cα-Oγ vector (S139, E/F) after superposition of the overall NS3 structure to a common reference in water (red) or in the presence of Ficoll (blue) or PEG (green) crowder without (A/C/E) and with (B/D/F) substrates. The correlation function was calculated as the second-order Legendre polynomial from the inner dot product of the orientational vector at different time points. Solid lines show simulation averages, standard errors are indicated by the shaded area. Results from double exponential fits to the correlation functions are given in **S8 Table**. Only trajectories with the reduced friction were used in this analysis (**S1 Table**).
(TIF)

S1 Table Simulations of NS3/4A under different conditions analyzed in this study. (PDF)

S2 Table Average Cα coordinate root mean square deviations of NS3/4A. (PDF)

S3 Table Average radius of gyration for NS3, NS4A, substrate, and crowders from heavy atoms. (PDF)

S4 Table Average secondary structure content for NS4A, and substrate calculated based on VMD Timeline results (the helix % includes both ⍺ and 3_10_ helix structures). (PDF)

S5 Table Single and double-exponential fits to rotational correlation functions for enzyme and substrates. (PDF)

S6 Table NS3-crowder contact life-times from single- and double-exponential fits to contact survival decays. (PDF)

S7 Table Substrate-crowder contact life-times from double-exponential fits to contact survival decays. (PDF)

S8 Table Double-exponential fits to the combined orientational correlation functions of the H57, D81, and S139 active site side chains. (PDF)

S9 Table Cluster analysis for substrate binding near the active site in simulations without crowders. (PDF)

S10 Table Cluster analysis for substrate binding near the active site in simulations with PEG crowders. (PDF)

S11 Table Cluster analysis for substrate binding near the active site in simulations with Ficoll crowders. (PDF)

## References

[1] Zhou H-X, Rivas G, Minton AP. Macromolecular crowding and confinement: biochemical, biophysical, and potential physiological consequences. Annu Rev Biophys. 2008;37: 375–397. doi: 10.1146/annurev.biophys.37.032807.125817 18573087 PMC2826134

[2] Zimmerman SB, Trach SO. Estimation of macromolecule concentrations and excluded volume effects for the cytoplasm of Escherichia coli. J Mol Biol. 1991;222: 599–620. doi: 10.1016/0022-2836(91)90499-v 1748995

[3] Ellis RJ, Minton AP. Join the crowd. Nature. 2003;425: 27–28. 12955122 10.1038/425027a

[4] Yu I, Mori T, Ando T, Harada R, Jung J, Sugita Y, et al. Biomolecular interactions modulate macromolecular structure and dynamics in atomistic model of a bacterial cytoplasm. Elife. 2016;5: 1–22. doi: 10.7554/eLife.19274 27801646 PMC5089862

[5] Rivas G, Minton AP. Macromolecular crowding in vitro, in vivo, and in between. Trends Biochem Sci. 2016;41: 970–981. doi: 10.1016/j.tibs.2016.08.013 27669651 PMC5804487

[6] Kuznetsova IM, Turoverov KK, Uversky VN. What macromolecular crowding can do to a protein. Int J Mol Sci. 2014;15: 23090–140. doi: 10.3390/ijms151223090 25514413 PMC4284756

[7] Minton AP. The Influence of Macromolecular Crowding and Macromolecular Confinement on Biochemical Reactions in Physiological Media. J Biol Chem. 2001;276: 10577–10580. doi: 10.1074/jbc.R100005200 11279227

[8] Schnell S, Turner TE. Reaction kinetics in intracellular environments with macromolecular crowding: simulations and rate laws. Prog Biophys Mol Biol. 2004;85: 235–260. doi: 10.1016/j.pbiomolbio.2004.01.012 15142746

[9] Aumiller WM, Davis BW, Hatzakis E, Keating CD. Interactions of macromolecular crowding agents and cosolutes with small-molecule substrates: Effect on horseradish peroxidase activity with two different substrates. J Phys Chem B. 2014;118: 10624–10632. doi: 10.1021/jp506594f 25157999 PMC4161143

[10] Akabayov SR, Akabayov B, Richardson CC, Wagner G. Molecular crowding enhanced ATPase activity of the RNA helicase eIF4A correlates with compaction of its quaternary structure and association with eIF4G. J Am Chem Soc. 2013;135: 10040–10047. doi: 10.1021/ja404404h 23767688 PMC3830938

[11] Rastogi H, Chowdhury PK. Understanding enzyme behavior in a crowded scenario through modulation in activity, conformation and dynamics. Biochim Biophys Acta—Proteins Proteomics. 2021;1869: 140699. doi: 10.1016/j.bbapap.2021.140699 34298166

[12] Qu Y, Bolen D. Efficacy of macromolecular crowding in forcing proteins to fold. Biophys Chem. 2002;101–102: 155–165. doi: 10.1016/s0301-4622(02)00148-5 12487997

[13] Maximova K, Wojtczak J, Trylska J. Enzyme kinetics in crowded solutions from isothermal titration calorimetry. Anal Biochem. 2019;567: 96–105. doi: 10.1016/j.ab.2018.11.006 30439369

[14] Maximova K, Wojtczak J, Trylska J. Enzymatic activity of human immunodeficiency virus type 1 protease in crowded solutions. Eur Biophys J. 2019;48: 685–689. doi: 10.1007/s00249-019-01392-1 31463540 PMC6742607

[15] Popielec A, Ostrowska N, Wojciechowska M, Feig M, Trylska J. Crowded environment affects the activity and inhibition of the NS3/4A protease. Biochimie. 2020;176: 169–180. doi: 10.1016/j.biochi.2020.07.009 32717410

[16] Długosz M, Trylska J. Diffusion in crowded biological environments: applications of Brownian dynamics. BMC Biophys. 2011;4: 3. doi: 10.1186/2046-1682-4-3 21595998 PMC3093676

[17] Ostrowska N, Feig M, Trylska J. Modeling Crowded Environment in Molecular Simulations. Front Mol Biosci. 2019;6. doi: 10.3389/fmolb.2019.00086 31572730 PMC6749006

[18] Frembgen-Kesner T, Elcock AH. Computer simulations of the bacterial cytoplasm. Biophys Rev. 2013;5: 109–119. doi: 10.1007/s12551-013-0110-6 23914257 PMC3728174

[19] Feig M, Yu I, Wang P, Nawrocki G, Sugita Y. Crowding in Cellular Environments at an Atomistic Level from Computer Simulations. J Phys Chem B. 2017;121(34): 8009–8025. doi: 10.1021/acs.jpcb.7b03570 28666087 PMC5582368

[20] Feig M, Sugita Y. Reaching new levels of realism in modeling biological macromolecules in cellular environments. J Mol Graph Model. 2013;45: 144–156. doi: 10.1016/j.jmgm.2013.08.017 24036504 PMC3815448

[21] Elcock AH. Atomic-level observation of macromolecular crowding effects: escape of a protein from the GroEL cage. Proc Natl Acad Sci USA. 2003;100: 2340–2344. doi: 10.1073/pnas.0535055100 12601146 PMC151342

[22] Kondrat S, Zimmermann O, Wiechert W, Lieres E Von. The effect of composition on diffusion of macromolecules in a crowded environment. Phys Biol. 2015;12: 046003. doi: 10.1088/1478-3975/12/4/046003 26020120

[23] Ridgway D, Broderick G, Lopez-Campistrous A, Ru’aini M, Winter P, Hamilton M, et al. Coarse-Grained Molecular Simulation of Diffusion and Reaction Kinetics in a Crowded Virtual Cytoplasm. Biophys J. 2008;94: 3748–3759. doi: 10.1529/biophysj.107.116053 18234819 PMC2367169

[24] Sarkar M, Li C, Pielak GJ. Soft interactions and crowding. Biophys Rev. 2013;5(2): 187–194. doi: 10.1007/s12551-013-0104-4 28510157 PMC5418433

[25] Harada R, Tochio N, Kigawa T, Sugita Y, Feig M. Reduced native state stability in crowded cellular environment due to protein-protein interactions. J Am Chem Soc. 2013;135: 3696–3701. doi: 10.1021/ja3126992 23402619 PMC3601481

[26] Gnutt D, Ebbinghaus S. The macromolecular crowding effect–from in vitro into the cell. 2016;397: 37–44. doi: 10.1515/hsz-2015-0161 26351910

[27] Speer SL, Stewart CJ, Sapir L, Harries D, Pielak GJ. Macromolecular Crowding Is More than Hard-Core Repulsions. Annu Rev Biophys. 2022;51: 267–300. doi: 10.1146/annurev-biophys-091321-071829 35239418

[28] Stadmiller SS, Aguilar JS, Parnham S, Pielak GJ. Protein-Peptide Binding Energetics under Crowded Conditions. J Phys Chem B. 2020;124: 9297–9309. doi: 10.1021/acs.jpcb.0c05578 32936642

[29] Kasahara K, Re S, Nawrocki G, Oshima H, Mishima-Tsumagari C, Miyata-Yabuki Y, et al. Reduced efficacy of a Src kinase inhibitor in crowded protein solution. Nat Commun. 2021;12: 1–8. doi: 10.1038/s41467-021-24349-5 34215742 PMC8253829

[30] Derham BK, Harding JJ. The effect of the presence of globular proteins and elongated polymers on enzyme activity. Biochim Biophys Acta—Proteins Proteomics. 2006;1764: 1000–1006. doi: 10.1016/j.bbapap.2006.01.005 16720113

[31] Acosta LC, Perez Goncalves GM, Pielak GJ, Gorensek-Benitez AH. Large cosolutes, small cosolutes, and dihydrofolate reductase activity. Protein Sci. 2017;26: 2417–2425. doi: 10.1002/pro.3316 28971539 PMC5699487

[32] Lin C. HCV NS3-4A Serine Protease. In: Tan S-L, editor. Hepatitis C Viruses: Genomes and Molecular Biology. Norfolk (UK): Horizon Bioscience; 2006. pp. 163–206.

[33] Hijikata M, Mizushima H, Akagi T, Mori S, Kakiuchi N, Kato N, et al. Two distinct proteinase activities required for the processing of a putative nonstructural precursor protein of hepatitis C virus. J Virol. 1993;67: 4665–4675. doi: 10.1128/JVI.67.8.4665-4675.1993 8392606 PMC237852

[34] Brass V, Berke JM, Montserret R, Blum HE, Penin F, Moradpour D. Structural determinants for membrane association and dynamic organization of the hepatitis C virus NS3-4A complex. Proc Natl Acad Sci USA. 2008;105(38): 14545–14550. doi: 10.1073/pnas.0807298105 18799730 PMC2567209

[35] Ostrowska N, Feig M, Trylska J. Crowding affects structural dynamics and contributes to membrane association of the NS3/4A complex. Biophys J. 2021;120: 3795–3806. doi: 10.1016/j.bpj.2021.07.008 34270995 PMC8456185

[36] LaPlante SR, Nar H, Lemke CT, Jakalian A, Aubry N, Kawai SH. Ligand Bioactive Conformation Plays a Critical Role in the Design of Drugs That Target the Hepatitis C Virus NS3 Protease. J Med Chem. 2014;57(5): 1777–1789. doi: 10.1021/jm401338c 24144444

[37] Abian O, Vega S, Neira JL, Velazquez-Campoy A. Conformational Stability of Hepatitis C Virus NS3 Protease. Biophys J. 2010;99(11): 3811–3820. doi: 10.1016/j.bpj.2010.10.037 21112306 PMC2998608

[38] Pettersen EF, Goddard TD, Huang CC, Couch GS, Greenblatt DM, Meng EC, et al. UCSF Chimera visualization system for exploratory research and analysis. J Comput Chem. 2004;25(13): 1605–1612. 15264254 10.1002/jcc.20084

[39] Berkholz DS, Shapovalov M V, Dunbrack RL Jr, Karplus AP. Conformation Dependence of Backbone Geometry in Proteins. Structure. 2009;17(10): 1278–1279. 19836332 10.1016/j.str.2009.08.012 PMC2810841

[40] Fiser A, Do RK, Sali A. Modeling of loops in protein structures. Prot Sci. 2000;9: 1753–1773. doi: 10.1110/ps.9.9.1753 11045621 PMC2144714

[41] Shen M, Sali A. Statistical potential for assessment and prediction of protein structures. Prot Sci. 2006;15: 2507–2524. doi: 10.1110/ps.062416606 17075131 PMC2242414

[42] Stote RH, Karplus M. Zinc binding in proteins and solution: A simple but accurate nonbonded representation. Proteins Struct Funct Bioinforma. 1995;23: 12–31. doi: 10.1002/prot.340230104 8539245

[43] Calimet N, Simonson T. CysxHisy-Zn2+ interactions: Possibilities and limitations of a simple pairwise force field. J Mol Graph Model. 2006;24: 404–411. doi: 10.1016/j.jmgm.2005.10.006 16298534

[44] Brandt EG, Hellgren M, Brinck T, Bergman T, Edholm O. Molecular dynamics study of zinc binding to cysteines in a peptide mimic of the alcohol dehydrogenase structural zinc site. Phys Chem Chem Phys. 2009;11: 975–983. doi: 10.1039/b815482a 19177216

[45] Tjörnhammar R, Edholm O. Molecular dynamics simulations of Zn2+ coordination in protein binding sites. J Chem Phys. 2010;132: 1–9. doi: 10.1063/1.3428381 20515113

[46] Phillips JC, Braun R, Wang W, Gumbart J, Tajkhorshid E, Villa E, et al. Scalable molecular dynamics with NAMD. J Comput Chem. 2005;26: 1781–1802. doi: 10.1002/jcc.20289 16222654 PMC2486339

[47] Bakan A, Meireles LM, Bahar I. ProDy: Protein Dynamics Inferred from Theory and Experiments. Bioinformatics. 2011;27(11): 1575–1577. doi: 10.1093/bioinformatics/btr168 21471012 PMC3102222

[48] Huang J, Rauscher S, Nawrocki G, Ran T, Feig M, Groot BL De, et al. CHARMM36m: An improved force field for folded and intrinsically disordered proteins. Nat Methods. 2016;14(1): 71–73. doi: 10.1038/nmeth.4067 27819658 PMC5199616

[49] Lee H, Venable RM, MacKerell AD Jr, Pastor RW. Molecular Dynamics Studies of Polyethylene Oxide and Polyethylene Glycol: Hydrodynamic Radius and Shape Anisotropy. Biophys J. 2008;95(4): 1590–1599. doi: 10.1529/biophysj.108.133025 18456821 PMC2483782

[50] Leonard AN, Pastor RW, Klauda JB. Parameterization of the CHARMM All-Atom Force Field for Ether Lipids and Model Linear Ethers. J Phys Chem B. 2018;122(26): 6744–6754. doi: 10.1021/acs.jpcb.8b02743 29870257 PMC6295153

[51] Sponseller D, Blaisten-Barojas E. Solutions and Condensed Phases of PEG2000 from All-Atom Molecular Dynamics. J Phys Chem B. 2021;125: 12892–12901. doi: 10.1021/acs.jpcb.1c06397 34783248

[52] Guvench O, Mallajosyula SS, Raman EP, Hatcher E, Vanommeslaeghe K, Foster TJ, et al. CHARMM additive all-atom force field for carbohydrate derivatives and their utility in polysaccharide and carbohydrate-protein modeling. J Chem Theory Comput. 2011;7: 3162–3180. 22125473 10.1021/ct200328p PMC3224046

[53] Raman EP, Guvench O, MacKerell AD Jr. CHARMM Additive All-Atom Force Field for Glycosidic Linkages in Carbohydrates Involving Furanoses. J Phys Chem B. 2010;114: 12981–12994. doi: 10.1021/jp105758h 20845956 PMC2958709

[54] Feig M, Karanicolas J, Brooks CL III. MMTSB Tool Set: enhanced sampling and multiscale modeling methods for applications in structural biology. J Mol Graph Model. 2004;22(5): 377–395. doi: 10.1016/j.jmgm.2003.12.005 15099834

[55] Humphrey W, Dalke A, Schulten K. VMD: visual molecular dynamics. J Mol Graph. 1996;14: 33–38. Available: http://www.ncbi.nlm.nih.gov/pubmed/8744570 doi: 10.1016/0263-7855(96)00018-5 8744570

[56] Ryckaert JP, Ciccotti G, Berendsen HJC. Numerical integration of the cartesian equations of motion of a system with constraints: molecular dynamics of n-alkanes. J Comput Phys. 1977;23(3): 327–341.

[57] Eastman P, Swails J, Chodera JD, McGibbon RT, Zhao Y, Beauchamp KA, et al. OpenMM 7: Rapid development of high performance algorithms for molecular dynamics. PLOS Comput Biol. 2017;13(7): e1005659. doi: 10.1371/journal.pcbi.1005659 28746339 PMC5549999

[58] Feller SE, Zhang Y, Pastor RW, Brooks BR. Constant pressure molecular dynamics simulation: The Langevin piston method. J Chem Phys. 1995;103: 4613.

[59] Basconi JE, Shirts MR. Effects of temperature control algorithms on transport properties and kinetics in molecular dynamics simulations. J Chem Theory Comput. 2013;9: 2887–2899. doi: 10.1021/ct400109a 26583973

[60] Nawrocki G, Wang PH, Yu I, Sugita Y, Feig M. Slow-Down in Diffusion in Crowded Protein Solutions Correlates with Transient Cluster Formation. J Phys Chem B. 2017;121: 11072–11084. doi: 10.1021/acs.jpcb.7b08785 29151345 PMC5951686

[61] Darden T, York D, Pedersen L. Particle mesh Ewald: An Nlog(N) method for Ewald sums in large systems. J Chem Phys. 1993;98: 10089.

[62] Yeh IC, Hummer G. System-Size Dependence of Diffusion Coefficients and Viscosities from Molecular Dynamics Simulations with Periodic Boundary Conditions. J Phys Chem B. 2004;108: 15873–15879.

[63] Ortega A, Amoros D, de la Torre JG. Prediction of hydrodynamic and other solution properties of rigid proteins from atomic- and residue-level models. Biophys J. 2011;101: 892–898. doi: 10.1016/j.bpj.2011.06.046 21843480 PMC3175065

[64] Wong V, Case DA. Evaluating Rotational Diffusion From Protein MD Simulations. J Phys Chem B. 2008;112(19): 6013–6024. doi: 10.1021/jp0761564 18052365

[65] Brooks BR, Brooks CL, Mackerell AD, Nilsson L, Petrella RJ, Roux B, et al. CHARMM: The biomolecular simulation program. J Comput Chem. 2009. doi: 10.1002/jcc.21287 19444816 PMC2810661

[66] Frishman D, Argos P. Knowledge-based secondary structure assignment. Prot Struct Funct Genet. 1995;23: 566–579. 10.1002/prot.340230412 8749853

[67] Williams T, Welley C, et al. Gnuplot 5.2: an interactive plotting program. Available: http://gnuplot.sourceforge.net

[68] Feig M, Sugita Y. Variable interactions between protein crowders and biomolecular solutes are important in understanding cellular crowding. J Phys Chem B. 2012;116: 599–605. doi: 10.1021/jp209302e 22117862 PMC3257409

[69] Rodríguez A, Oliva C, González M. A comparative QM/MM study of the reaction mechanism of the Hepatitis C virus NS3/NS4A protease with the three main natural substrates NS5A/5B, NS4B/5A and NS4A/4B. Phys Chem Chem Phys. 2010;12: 8001–8015. doi: 10.1039/c002116d 20520921

[70] De Francesco R, Pessi A, Steinkühler C. Mechanisms of hepatitis C virus NS3 proteinase inhibitors. J Viral Hepat. 1999;6: 23–30. doi: 10.1046/j.1365-2893.1999.00002.x 10760031

[71] Dultz G, Shimakami T, Schneider M, Murai K, Yamane D, Marion A, et al. Extended interaction networks with HCV protease NS3-4A substrates explain the lack of adaptive capability against protease inhibitors. J Biol Chem. 2020;295: 13862–13874. doi: 10.1074/jbc.RA120.013898 32747444 PMC7535904

[72] Matthew A, Zephyr J, Nageswara Rao D, Henes M, Kamran W, Kosovrasti K, et al. Avoiding Drug Resistance by Substrate Envelope-Guided Design: Toward Potent and Robust HCV NS3/4A Protease Inhibitors. MBio. 2020;11: e00172–20. doi: 10.1128/mBio.00172-20 32234812 PMC7157764

