# Supplementary material for: Varying molecular interactions explain aspects of crowder-dependent enzyme function of a viral protease
Source: PLoS Comput Biol. 2023 Apr 25;19(4):e1011054. doi: 10.1371/journal.pcbi.1011054 (PMC10162569; doi:10.1371/journal.pcbi.1011054)
Supplement: S1 Text — (PDF) [file pcbi.1011054.s001.pdf]

Supplemental Methods for:

Varying molecular interactions explain aspects of crowder-dependent enzyme function of a viral protease

Natalia Ostrowska[1], Michael Feig[2,*], Joanna Trylska[1,*]

[1]Centre of New Technologies, University of Warsaw, Warsaw, Poland

[2]Department of Biochemistry and Molecular Biology, Michigan State University, East Lansing, MI, USA

**Corresponding authors:*

Joanna Trylska
Centre of New Technologies
University of Warsaw
02-097 Warsaw, Poland
Email: joanna@cent.uw.edu.pl
ORCID 0000-0002-1464-5323

Michael Feig
Department of Biochemistry and Molecular Biology
Michigan State University
East Lansing, MI 48824, USA
Email: mfeiglab@gmail.com
ORCID 0000-0001-9380-6422

Building the model of Ficoll crowders

Ficoll crowders were designed to match the size of PEG crowders used in our previous simulations of NS3/4A [1,2] in order to compare the effects of crowding agents of similar size but with different atom composition. We decided to build polysucrose crowders with comparable number of atoms as PEG crowders to achieve molecular weight equivalence rather than molecular volume equivalence. To that extent, we built a 204-atom polymer composed of four sucrose molecules connected with three glycerol linkers (**S1 Fig**). Such a molecule matches the 206-atom 28-mer of PEG. This polysucrose crowder also forms a branched structure, a known property of Ficoll polymers. We note that commercially available Ficoll is much larger, with molecular weights of either 70 or 400 kDa.

Since Ficoll is a commercial product, its exact structure has remained unclear [3]. However, we know that Ficoll is synthesized via reaction of sucrose with epichlorohydrin that leads to the formation of a branched polymer glycerols linking sucroses [4]. While the probabilities of attaching glycerol to different hydroxyl groups of a sucrose molecule are unclear, we made the assumption that hydroxyl groups attached to primary carbons of sucrose (GLC:C6, FRU:C1 and FRU:C6) would be more likely to react with epichlorohydrin than hydroxyl groups of secondary carbons. Thus, out of six sucrose-glycerol linking sites in our model, in four cases the terminal groups of glycerol attach to one of the primary carbons, and in two cases - to the C2 carbon of glucose, located opposite the C6 primary carbon. The final structure of our Ficoll-like crowder model used in the simulations is shown in **S1 Fig**.

Parameterization of the Ficoll crowders

Ficoll crowders were parameterized using the CHARMM force field for carbohydrates [5,6] (**S2 Fig**). For sucrose monomers, we used the parameters for α-D-glucose and β-fructofuranose of the AGLC and BFRU residues, along with the SUCR patch, specifically designed to link AGLC and BFRU forming the sucrose molecules. For glycerol the standard MGL residue topology was used.

To create sucrose-glycerol linkages, individual topology patches (shown below) were written for each sucrose-glycerol ester bond, *i.e.*, oxygen and two surrounding carbon atoms. The patch names are related to the atom names of glycerol and saccharide carbons; *e.g.*, the patch GG23 describes the ester bond between the C2 carbon of glucose and C3 carbon of glycerol.

CHARMM topology:

```
PRES GG23          -0.27 ! apply to GLC2:C2 -- MGL3:C3
dele atom 1HO2
dele atom 2O3
dele atom 2HO3
GROU
ATOM 1C2  CC3161    0.09 !   like MELZ:2C3
ATOM 1O2  OC301    -0.36
ATOM 2C3  CC322     0.00 !   like IMAL:2C6
BOND 1O2  2C3

PRES GF11          -0.36 ! apply to MGL:C1 -- FRU:C1
dele atom 1HO1
dele atom 1O1
dele atom 2HO1
GROU
ATOM 1C1  CC322     0.00  !   like IMAL:2C6
ATOM 2O1  OC301    -0.36
ATOM 2C1  CC321     0.00   !   like IMAL:2C6
BOND 1C1  2O1

PRES GF31          -0.36  ! apply to MGL:C3--FRU:C1
dele atom 1HO3
dele atom 1O3
dele atom 2HO1
GROU
ATOM 1C3  CC322     0.00  !   like IMAL:2C6
ATOM 2O1  OC301    -0.36
ATOM 2C1  CC321     0.00  !   like IMAL:2C6
BOND 1C3  2O1

PRES FG61           -0.36 ! apply to FRU:C6 -- MGL:C1
dele atom 1HO6
dele atom 2O3
dele atom 2HO3
GROU
ATOM 1C6  CC321     0.00  !   like IMAL:2C6
ATOM 1O6  OC301    -0.36
ATOM 2C1  CC322     0.00  !   like IMAL:2C6
BOND 1O6  2C1
```

The sucrose-glycerol linkages were parameterized by analogy to the molecules already parameterized in the CHARMM force field. Considering the structural similarity between the glycerol and C1O1H-C2O2H-C3O3H part of glucose (**S2 Fig**), we assigned the force field parameters based on the similarity to glycosidic bonds found in certain di- and trisaccharides.

All the ester bond oxygens were assigned the partial charge of -0.36 e and the C-O-C angle of 109.7^{o}. These are the standard values for glycosidic bonds linking glucose and fructose molecules.

Carbon atoms taking part in the ester bond formation were divided into two groups:

1. secondary carbons, bonding with two carbon atoms. This group includes the GLC:C2 atom, present in the GG23 patch. The C2 carbon was parameterized based on the 2C3 carbon in melezitose patch (MELZ), which is one of just a few cases in the CHARMM force field where the secondary carbon is involved in the glycosidic bond formation (**S3 Fig**).

2. primary carbons, bonding with one carbon atom. This group includes both the primary carbon of glucose and fructose (GLC:C6, FRU:C1, FRU:C6) and terminal carbons of glycerol (GLY:C1, GLY:C3). Each of those carbons were assigned partial charges of 0.0 and the type CC321, analogous to the 2C6 carbon of isomaltulose (IMAL).

The patches described here include only the atoms for which the charges are different from free monomers. After adding the appropriate number of hydrogen atoms, whose partial charge always equal 0.09 e, the total charges for each patch sum up to zero.

The missing angle and dihedral parameters, as well as one set of bond parameters, were assigned individually based on the similarity to already parameterized di- and trisaccharides containing fructose and glucose.

CHARMM parameters:

```
BONDS
CC3151  OC301    360.00  1.415   ! CC3162 OC302

ANGLES
CC3161  OC301   CC312    50.00   109.20  !
CC322   OC301   CC321    95.00   109.70
CC3161  OC301   CC322    95.00   109.70  ! IMAL 1C1-1O1-2C6
CC3051  CC3151  OC301    45.00   110.50  ! FRU OC303-C3-C2
HCA1    CC3151  OC301    60.00   109.50  ! FRU OC303-C3-HCA1
OC301   CC3151  CC3151   45.00   110.50  ! OC303 CC3151 CC3151
CC3151  OC301   CC322    95.00   109.70  ! IMAL 1C1-1O1-2C6

DIHEDRALS
CC3161  CC3161  OC301   CC312    0.13  1  180.0 ! CC3161 CC3161 OC301
CC3162
CC3161  CC3161  OC301   CC312    0.25  2  180.0
CC3161  CC3161  OC301   CC312    0.06  3  180.0

HCA1    CC312   OC301   CC3161   0.284 3    0.0 ! HCA2 CC321 OC301 CC3152
HCA1    CC3161  OC301   CC312    0.284 3    0.0 ! HCA1 CC3152 OC301 CC321

OC301   CC312   CC322   OC301    1.7749 1 180.00 ! OC301 CC312 CC322 OC311
OC301   CC312   CC322   OC301    1.5713 2   0.00
OC301   CC312   CC322   OC301    1.8214 3   0.00

CC3051  CC321   OC301   CC322    0.64  1  180.0 ! CC3051 CC321 OC301 CC3051
CC3051  CC321   OC301   CC322    0.03  2  180.0
CC3051  CC321   OC301   CC322    0.61  3    0.0
CC322   OC301   CC321   HCA2     0.284 3    0.0 ! CC331 OC301 CC321 HCA2

CC321   OC301   CC322   HCA2     0.20  3    0.0 ! CC322 CC312 CC322 HCA2

CC322   CC312   CC322   OC301    0.35  1    0.0 ! CC322 CC312 CC322 OC311
CC322   CC312   CC322   OC301    0.69  2    0.0
CC322   CC312   CC322   OC301    2.79  3  180.0
OC3C51  CC3051  CC3151  OC301    0.32  1  180.0 ! OC3C51 CC3051 CC3151 OC303
OC3C51  CC3051  CC3151  OC301    0.65  2  180.0 !
OC3C51  CC3051  CC3151  OC301    2.62  3    0.0 !

CC322   OC301   CC3151  CC3051   0.07  1  180.0 ! CC3162 OC303 CC3151 CC3051
CC322   OC301   CC3151  CC3051   0.04  2    0.0 !
CC322   OC301   CC3151  CC3051   0.14  3    0.0 !
CC321   CC3051  CC3151  OC301    0.94  1    0.0 ! CC321 CC3051 CC3151 OC303
CC321   CC3051  CC3151  OC301    1.59  2  180.0 !
CC321   CC3051  CC3151  OC301    0.84  3    0.0 !
CC3153  CC3151  CC3151  OC301    0.01  1  180.0 ! CC3153 CC3151 CC3151 OC303
CC3153  CC3151  CC3151  OC301    0.72  2    0.0 !               "
```

```
CC3153  CC3151  CC3151  OC301    0.73  3    0.0 !

CC322   OC301   CC3151  HCA1     0.284 3    0.0 ! CC3162 OC303 CC3151 HCA1
OC302   CC3051  CC3151  OC301    0.12  1  180.0 ! OC302 CC3051 CC3151 OC303
OC302   CC3051  CC3151  OC301    1.87  2  180.0 !
OC302   CC3051  CC3151  OC301    1.64  3  180.0 !
OC301   CC3151  CC3151  HCA1     0.14  3    0.0 ! OC311 CC3151 CC3151 HCA1
OC301   CC3151  CC3151  OC311    2.87  1  180.0 ! OC311 CC3151 CC3151 OC311
OC301   CC3151  CC3151  OC311    0.03  2    0.0 !
OC301   CC3151  CC3151  OC311    0.23  3    0.0 !

CC3151  OC301   CC322   HCA2     0.284 3    0.0 ! CC3062 OC301 CC321 HCA2
CC3161  OC301   CC322   HCA2     0.284 3    0.0 ! CC3062 OC301 CC321 HCA2

CC3161  CC3161  OC301   CC322    0.13  1  180.0 ! CC3161 CC3161 OC301 CC3162
CC3161  CC3161  OC301   CC322    0.25  2  180.0 !
CC3161  CC3161  OC301   CC322    0.06  3  180.0

CC3162  CC3161  OC301   CC322    0.13  1  180.0 ! CC3161 CC3161 OC301 CC3162
CC3162  CC3161  OC301   CC322    0.25  2  180.0 !
CC3162  CC3161  OC301   CC322    0.06  3  180.0

HCA1    CC3161  OC301   CC322    0.284 3    0.0 ! HCA1 CC3161 OC301 CC3152
CC322   OC301   CC3151  CC3151   0.07  1  180.0 ! CC3162 OC303 CC3151 CC3151
CC322   OC301   CC3151  CC3151   0.04  2    0.0
CC322   OC301   CC3151  CC3151   0.14  3    0.0

OC301   CC3161  CC3162  OC302    0.59  1  180.0 ! OC301 CC3161 CC3162 OC301
OC301   CC3161  CC3162  OC302    1.16  2    0.0

CC322   CC312   OC301   CC3161   0.40  1  180.0 ! CC321 CC3051 OC302 CC3162
CC322   CC312   OC301   CC3161   0.48  2    0.0 !
CC322   CC312   OC301   CC3161   0.19  3    0.0 !

CC312   CC322   OC301   CC321    0.64  1  180.0 ! CC3051 CC321 OC301 CC3051
CC312   CC322   OC301   CC321    0.03  2  180.0
CC312   CC322   OC301   CC321    0.61  3    0.0

CC312   CC322   OC301   CC3151   0.64  1  180.0 ! CC3051 CC321 OC301 CC3051
CC312   CC322   OC301   CC3151   0.03  2  180.0
CC312   CC322   OC301   CC3151   0.61  3    0.0

CC312   CC322   OC301   CC3161   0.64  1  180.0 ! CC3051 CC321 OC301 CC3051
CC312   CC322   OC301   CC3161   0.03  2  180.0
CC312   CC322   OC301   CC3161   0.61  3    0.0

CC3153  CC321   OC301   CC322    0.64  1  180.0 ! CC3153 CC321 OC301 CC3162
CC3153  CC321   OC301   CC322    0.03  2  180.0
CC3153  CC321   OC301   CC322    0.61  3    0.0
```

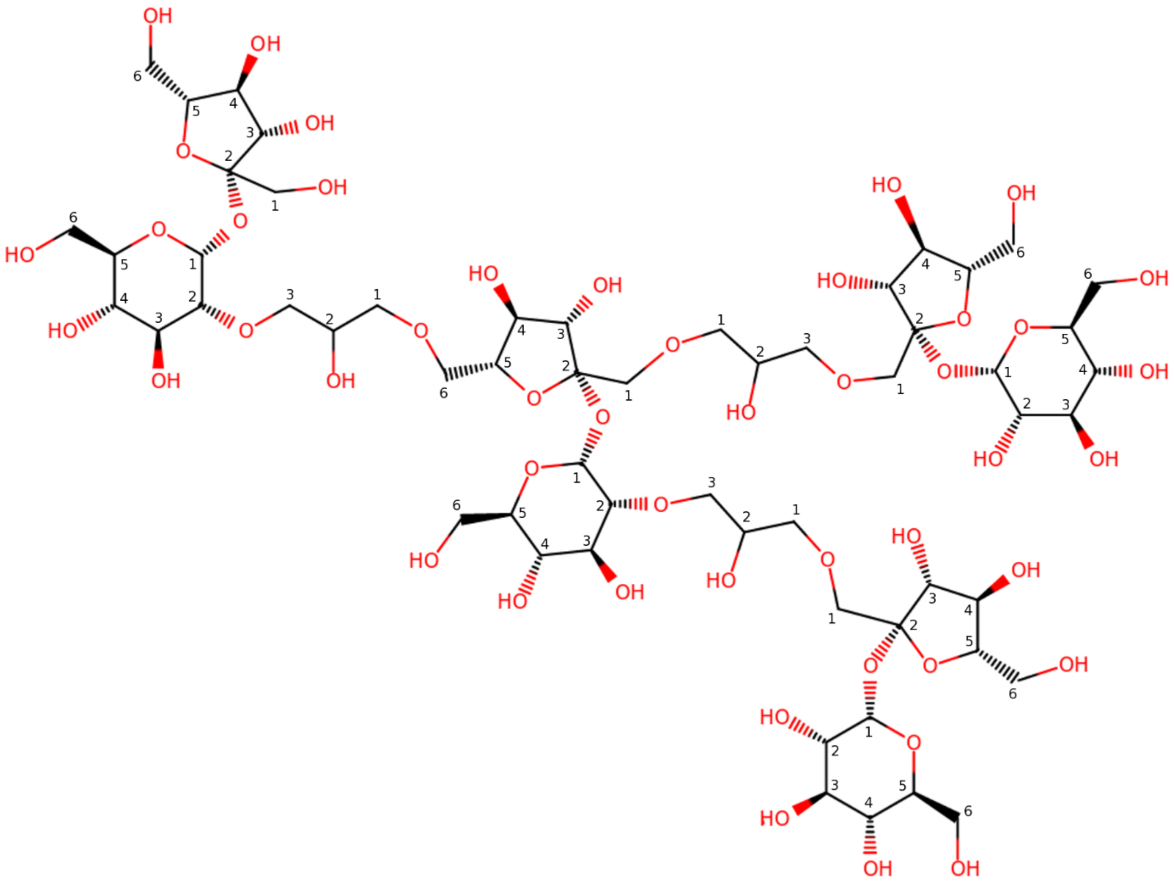

S1 Fig. Chemical structure of the polysucrose molecule used as a model for Ficoll. The sucrose molecules are connected with glycerol linkers. Carbon atoms are annotated according to the numbers used in the CHARMM force field.

glucose, AGLC

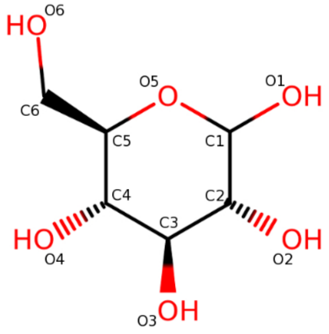

fructose, BFRU

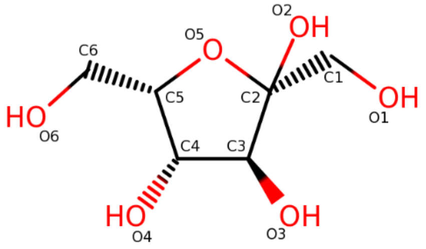

sucrose

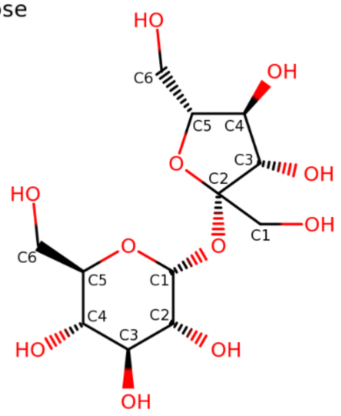


```
PRES SUCR          0.00 ! apply to AGLC,BFRU
!dele atom 1HO1
!dele atom 2O2
!dele atom 2HO2
GROU
ATOM 1C1  CC3162    0.29 !
ATOM 1O1  OC302    -0.36 !
ATOM 2C2  CC3051    0.38 !
ATOM 1H1  HCA1      0.09 !
ATOM 1C5  CC3163    0.11 !
ATOM 1H5  HCA1      0.09 !
ATOM 1O5  OC3C61   -0.40 !
ATOM 2O5  OC3C51   -0.40 !
ATOM 2C5  CC3153    0.11 !
ATOM 2H5  HCA1      0.09 !
BOND 1O1  2C2
```

S2 Fig. Building blocks used to parameterize Ficoll molecules. Glucose and fructose are shown, together forming sucrose molecules. All structures are shown along with atom names used in the CHARMM topology.

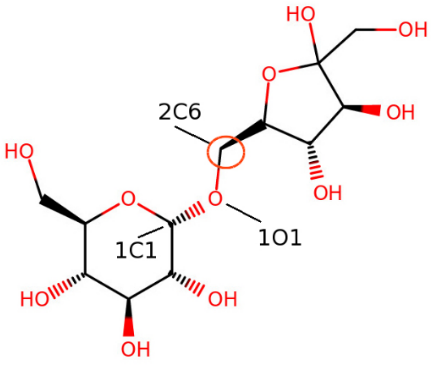


```
PRES IMAL        -0.07 ! pram apply to AGLC,BFRU
dele atom 1HO1
dele atom 2O6
dele atom 2HO6
ATOM 1C1  CC3162    0.29
ATOM 1O1  OC301    -0.36
ATOM 2C6  CC321     0.00
BOND 1O1  2C6
```

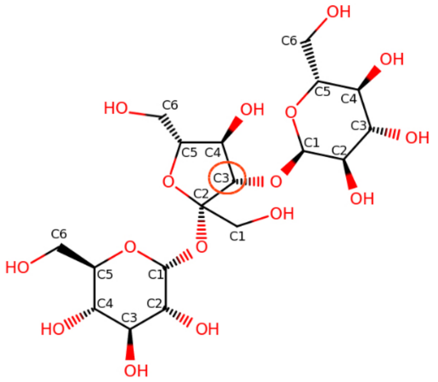


```
PRES MELZ         0.33 !  apply to AGLC,BFRU,AGLC
dele atom 1HO1
dele atom 2O2
dele atom 2HO2
dele atom 3HO1
dele atom 2O3
dele atom 2HO3
ATOM 1C1  CC3162    0.29 !
ATOM 1O1  OC302    -0.36 !
ATOM 2C2  CC3051    0.38 !
ATOM 3C1  CC3162    0.29 !
ATOM 3O1  OC303    -0.36 !
ATOM 2C3  CC3151    0.09 !
BOND 1O1  2C2  3O1  2C3
```

S3 Fig. Structures of isomaltulose and melezitose and CHARMM force field patches. The patches are used to form these molecules from glucose and fructose monomers. The 2C6 and 2C3 atoms used to parameterize carbon atoms in the model of Ficoll are marked with orange circles.

References

[1] A. Popielec, N. Ostrowska, M. Wojciechowska, M. Feig, J. Trylska, Crowded environment affects the activity and inhibition of the NS3/4A protease. *Biochimie*, 176:169-180, 2020

[2] N. Ostrowska, M. Feig, J. Trylska, Crowding affects structural dynamics and contributes to membrane association of the NS3/4A complex. *Biophysical Journal*, 120(17):3795-3806, 2021

[3] H. Holter, K. MaxMøller, A substance for aqueous density gradients, *Experimental Cell Research*, 15(3):631-632, 1958

[4] L. Holmberg, B. Lindberg, B. Lindqvist, The reaction between epichlorohydrin and polysaccharides: Part 2, synthesis of some model substances, with cyclic substituents, *Carbohydrate Research*, 268(1):47-56

[5] O. Guvench, S. S. Mallajosyula, E. P. Raman, E. Hatcher, K. Vanommeslaeghe, T. J. Foster, F. W. Jamison II, A. D. MacKerell Jr. CHARMM additive all-atom force field for carbohydrate derivatives and their utility in polysaccharide and carbohydrate-protein modeling. *Journal of Chemical Theory and Computation*, 7:3162–3180, 2011.

[6] E. P. Raman, O. Guvench, A. D. MacKerell Jr. CHARMM additive all-atom force field for glycosidic linkages in carbohydrates involving furanoses. *Journal of Physical Chemistry B*, 114:12981–12994, 2010.
