# Supplementary figures and images for: Varying molecular interactions explain aspects of crowder-dependent enzyme function of a viral protease
Source: PLoS Comput Biol. 2023 Apr 25;19(4):e1011054. doi: 10.1371/journal.pcbi.1011054 (PMC10162569; doi:10.1371/journal.pcbi.1011054)

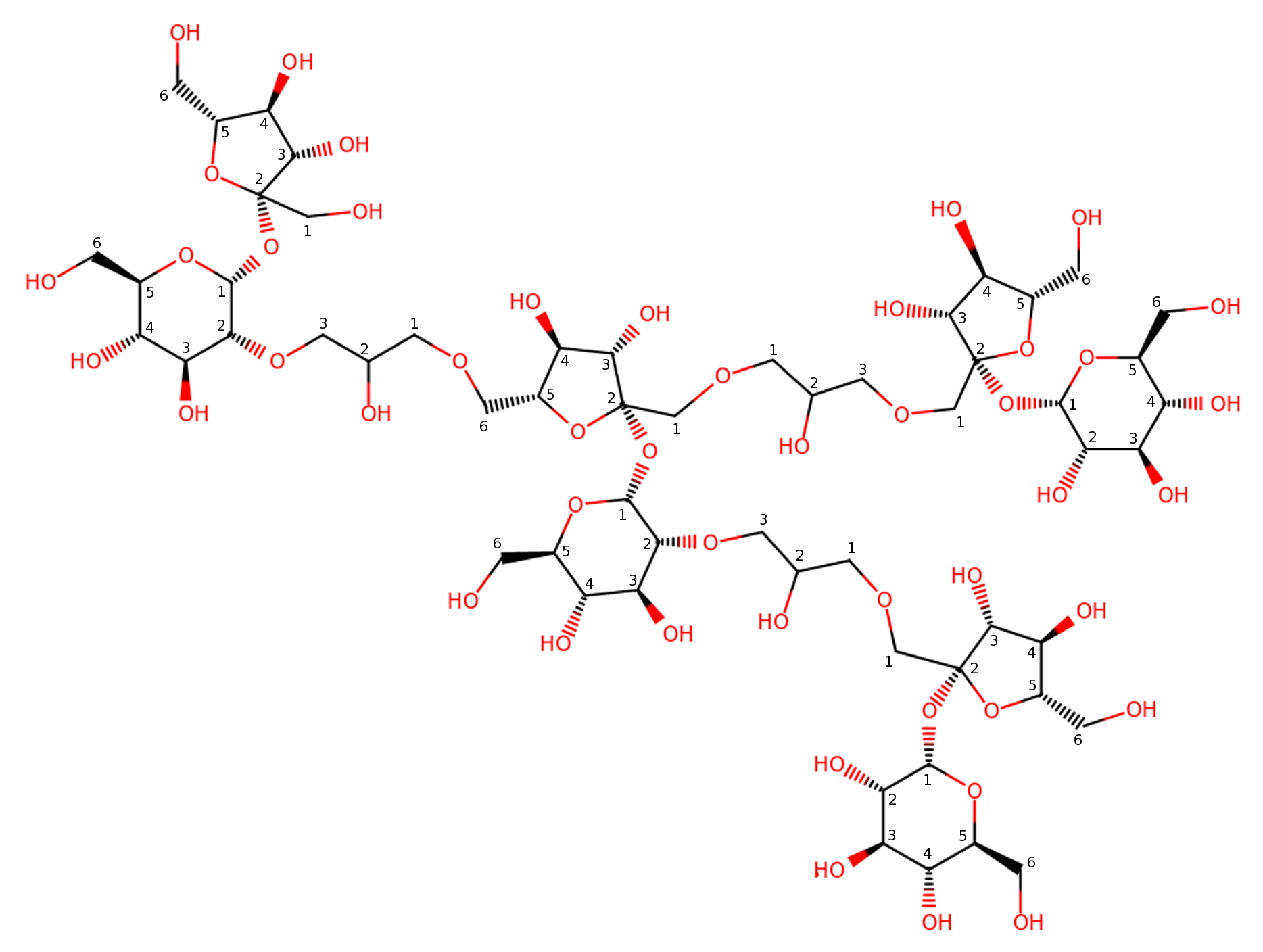

Supplement: S1 Fig — The sucrose molecules are connected with glycerol linkers. Carbon atoms are annotated according to the numbers used in the CHARMM force field. (TIF) [file pcbi.1011054.s002.tif]

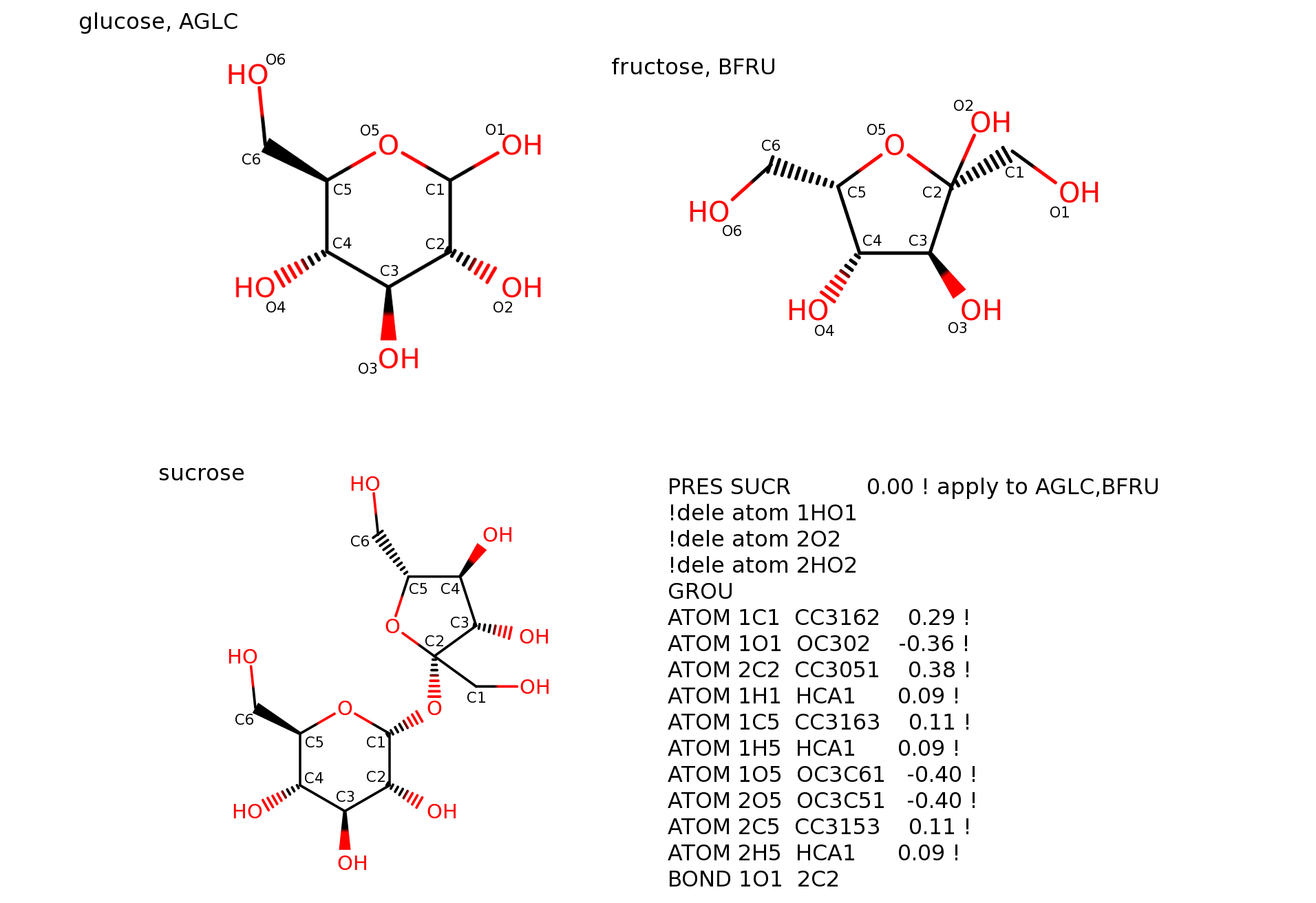

Supplement: S2 Fig — Glucose and fructose are shown, together forming sucrose molecules. All structures are shown along with atom names used in the CHARMM topology. (TIF) [file pcbi.1011054.s003.tif]

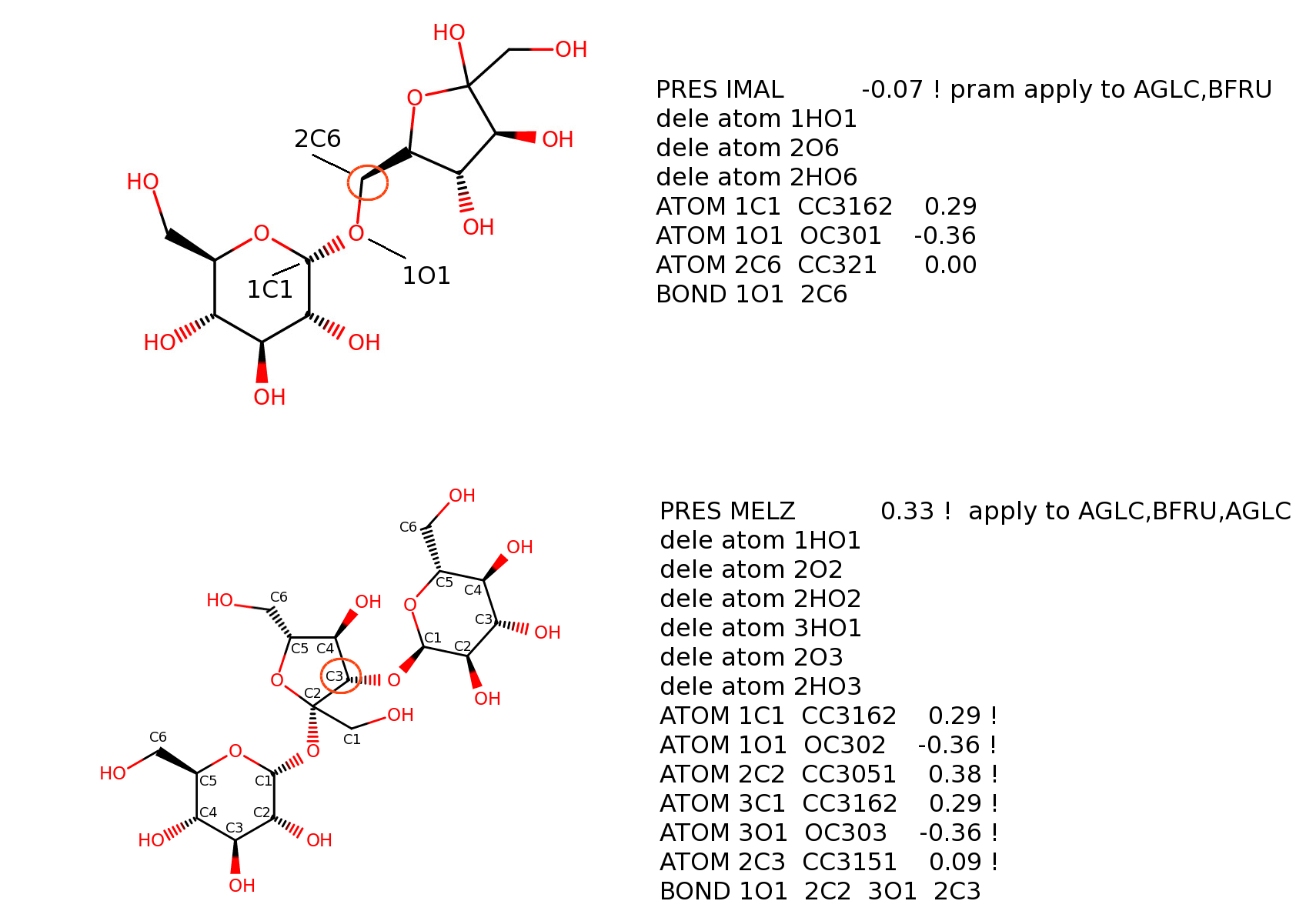

Supplement: S3 Fig — The patches are used to form these molecules from glucose and fructose monomers. The 2C6 and 2C3 atoms used to parameterize carbon atoms in the model of Ficoll are marked with orange circles. (TIF) [file pcbi.1011054.s004.tif]

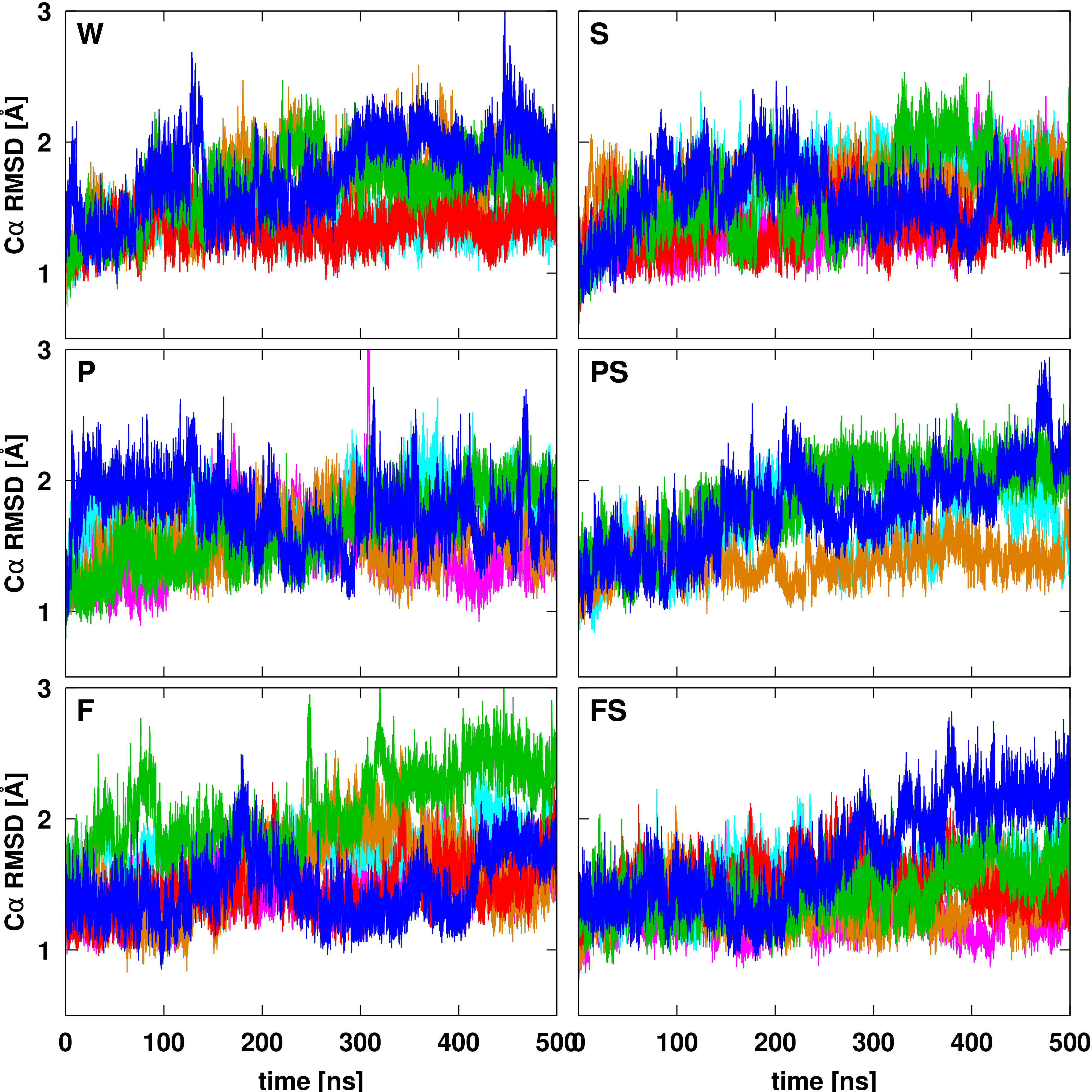

Supplement: S4 Fig — RMSD values are shown in water (W), in the presence of PEG (P) and Ficoll (F) and with substrates (S, PS, FS). RMSD values are based on Cα coordinates after optimal superposition with respect to the experimental structure (PDB ID: 4JMY) for NS3 and the central 13 residues of NS4A for which structure information is available in the PDB. Different colors distinguish individual trajectories. (TIF) [file pcbi.1011054.s005.tif]

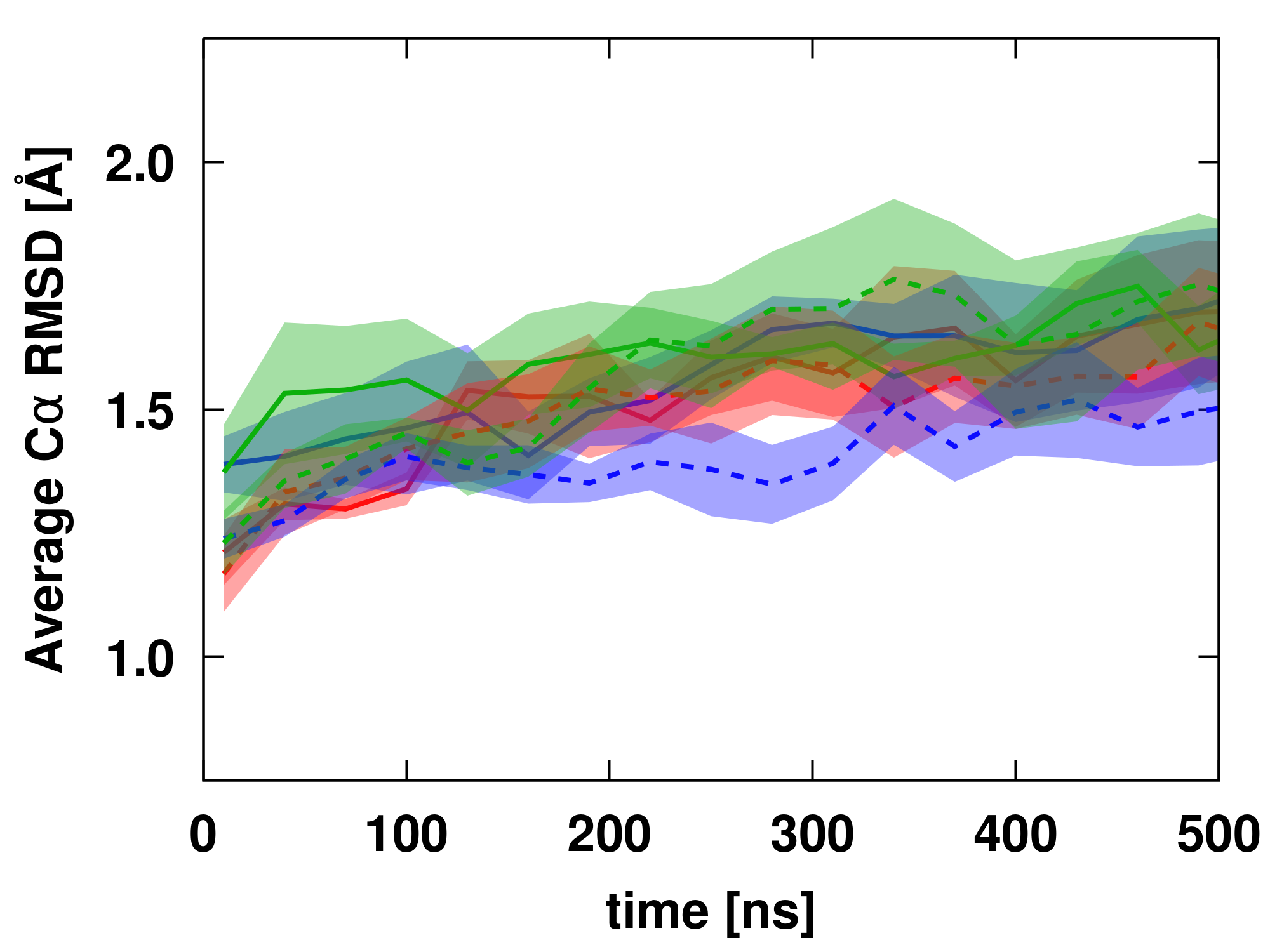

Supplement: S5 Fig — Results are shown for NS3/4A in water (red), in the presence of PEG (green) or Ficoll (blue). Solid and dashed lines are from simulations in the absence and presence of substrates, respectively. Averages were calculated over 20 ns trajectory segments. Error bars indicate the standard errors of the mean from variations between replicate simulations. (TIF) [file pcbi.1011054.s006.tif]

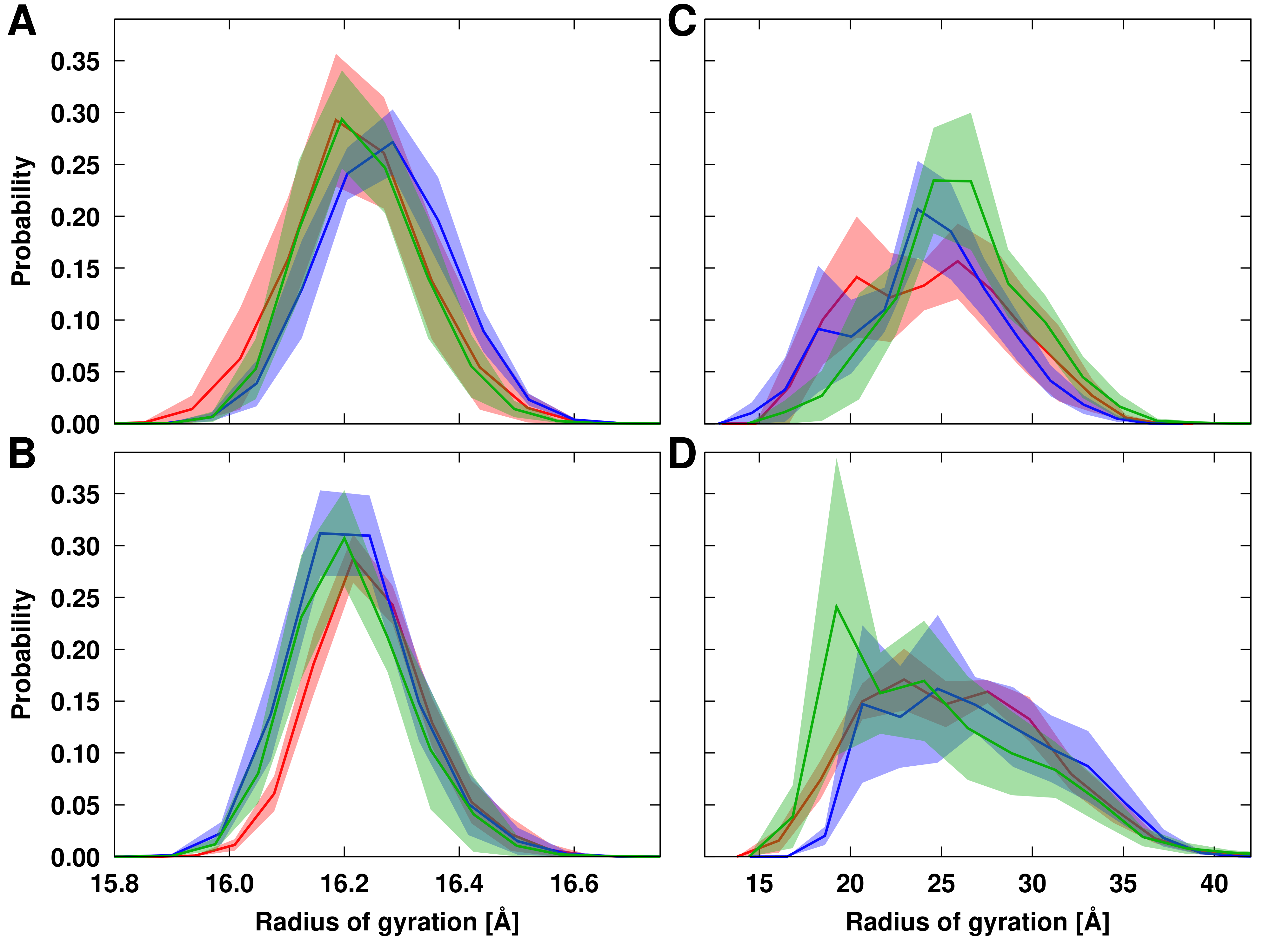

Supplement: S6 Fig — Results are shown for NS3 (A,B) and NS4A (C,D) without (A,C) and with (B,D) substrates in water (red) or in the presence of PEG (green) or Ficoll (blue) crowders. Individual histograms were averaged over replicate trajectories. Error bars indicate standard errors for each bin. (TIF) [file pcbi.1011054.s007.tif]

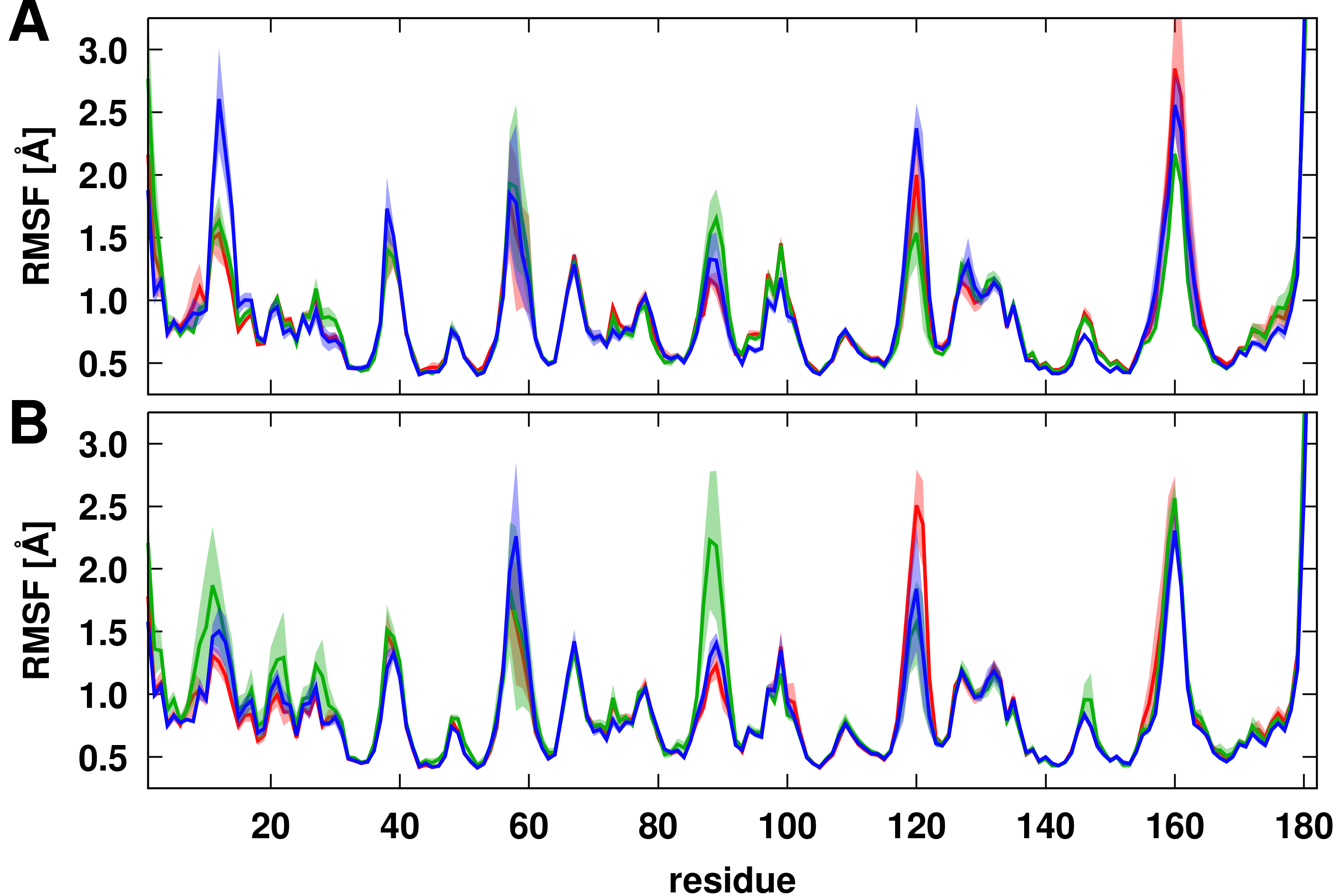

Supplement: S7 Fig — Results are shown without (A) and with (B) substrates in water (red) or in the presence of PEG (green) or Ficoll (blue) crowders. RMSF was calculated from Cα atoms with respect to the average structures of NS3 for each trajectory after superposition onto a reference structure. Solid lines reflect trajectory averages, shaded areas indicate standard errors of the mean. (TIF) [file pcbi.1011054.s008.tif]

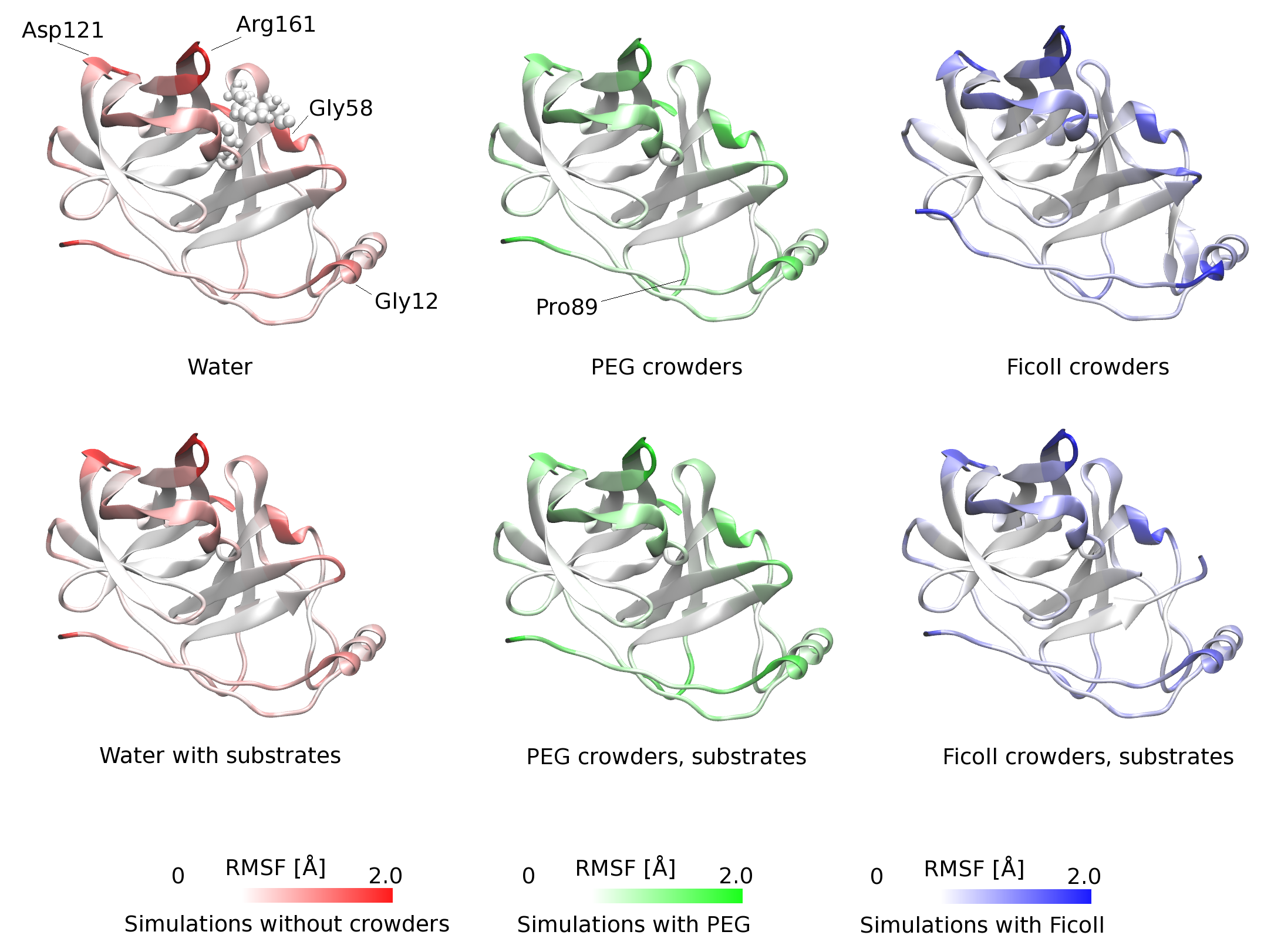

Supplement: S8 Fig — RMSF values were calculated for the C⍺ atoms of each amino acid, with respect to the average structure for each trajectory, after superposition onto the crystallographic structure (PDB ID: 4JMY). For clarity, in all cases the higher end of the RMSF scale (dark colors) in the legends correspond to RMSF values between 2.0 and the maximum value of 5.23 Å. (TIF) [file pcbi.1011054.s009.tif]

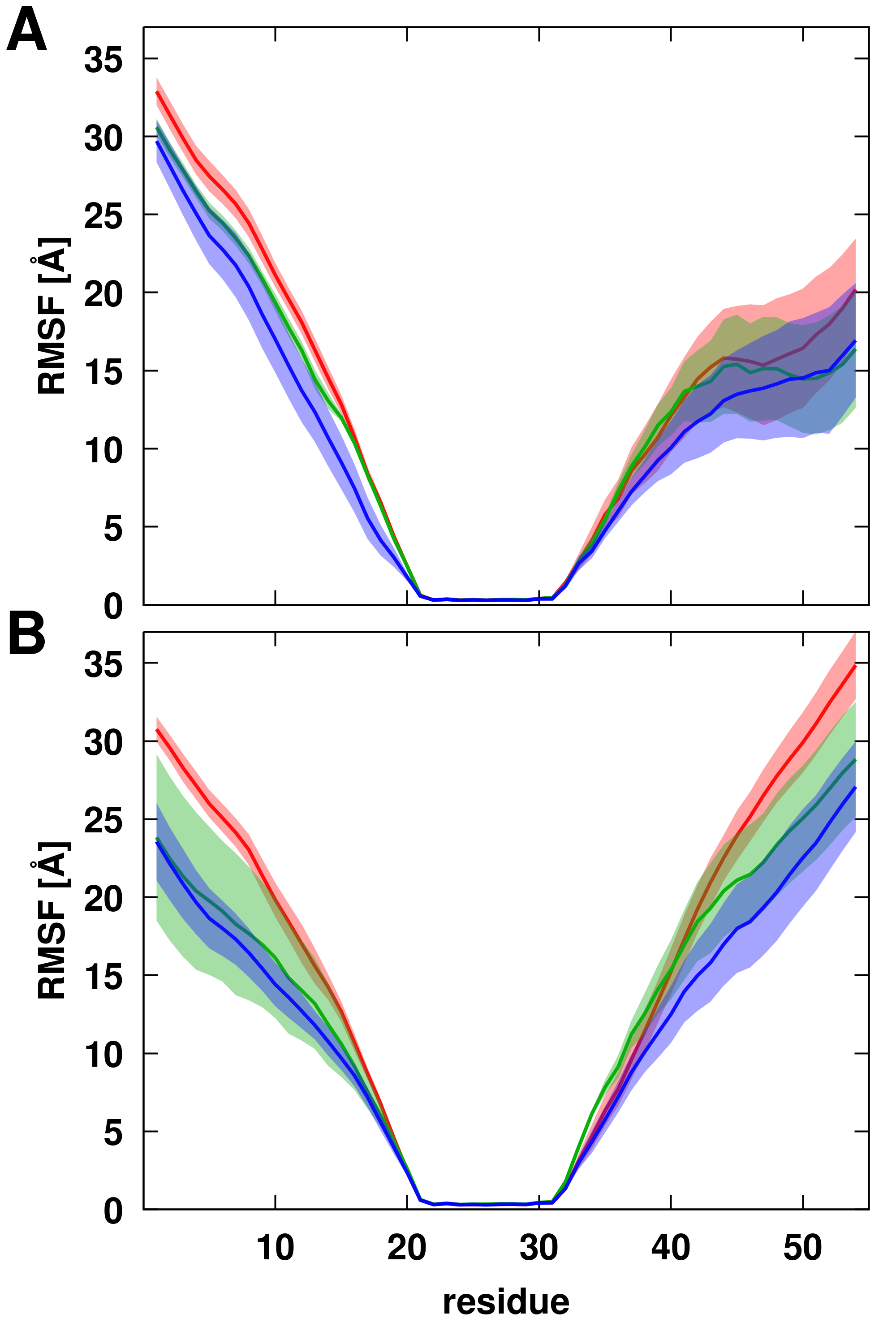

Supplement: S9 Fig — Results are shown without (A) and with (B) substrates in water (red) or in the presence of PEG (green) or Ficoll (blue) crowders. RMSF was calculated from Cα atoms with respect to the average structures of NS4A for each trajectory after superposition of the ordered central residues (21–31). Solid lines reflect trajectory averages, shaded areas indicate standard errors of the mean. (TIF) [file pcbi.1011054.s010.tif]

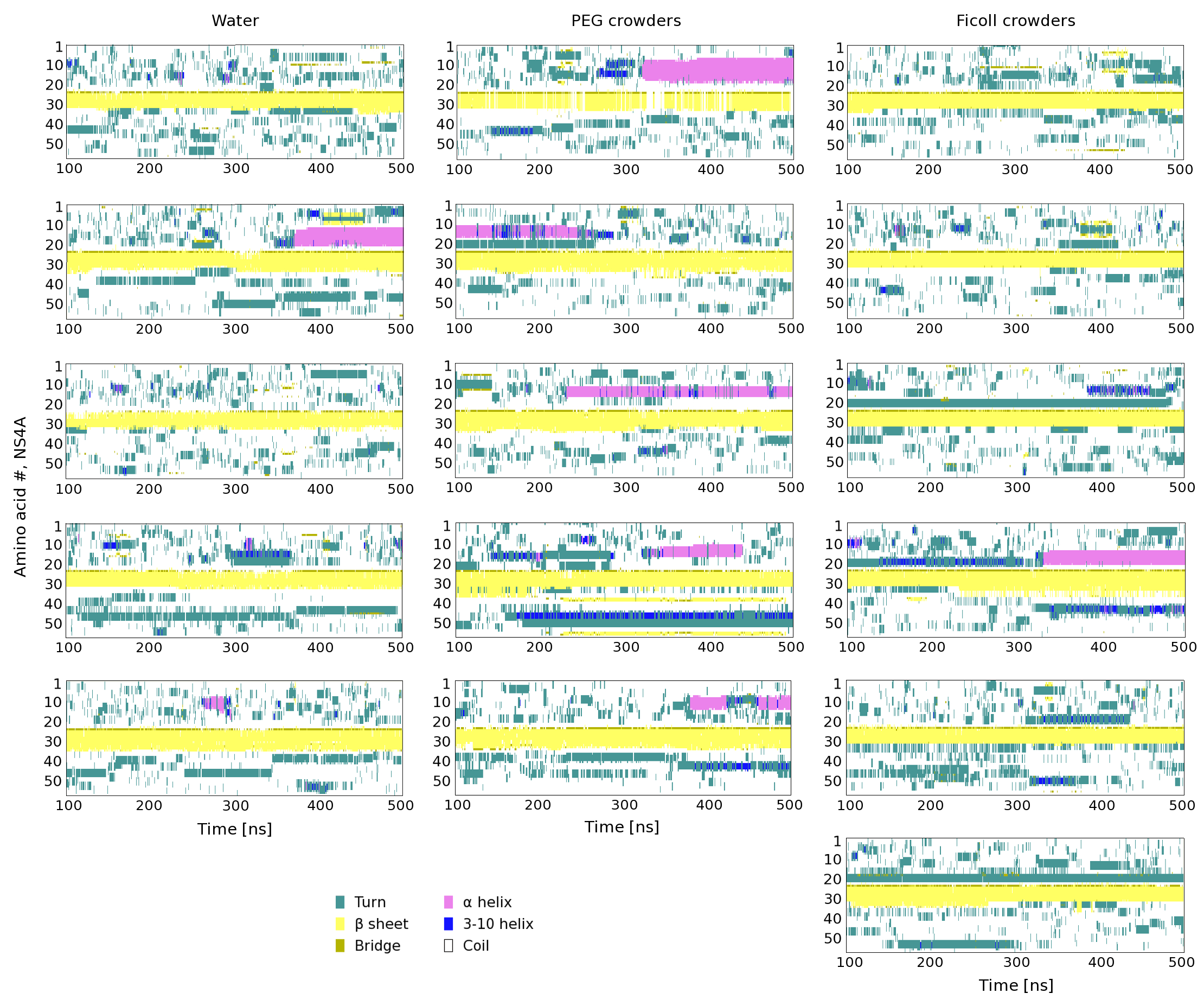

Supplement: S10 Fig — Helical conformations are marked in pink or blue, β-strands in yellow, and turns in cyan. Each graph shows data from one MD trajectory. (TIF) [file pcbi.1011054.s011.tif]

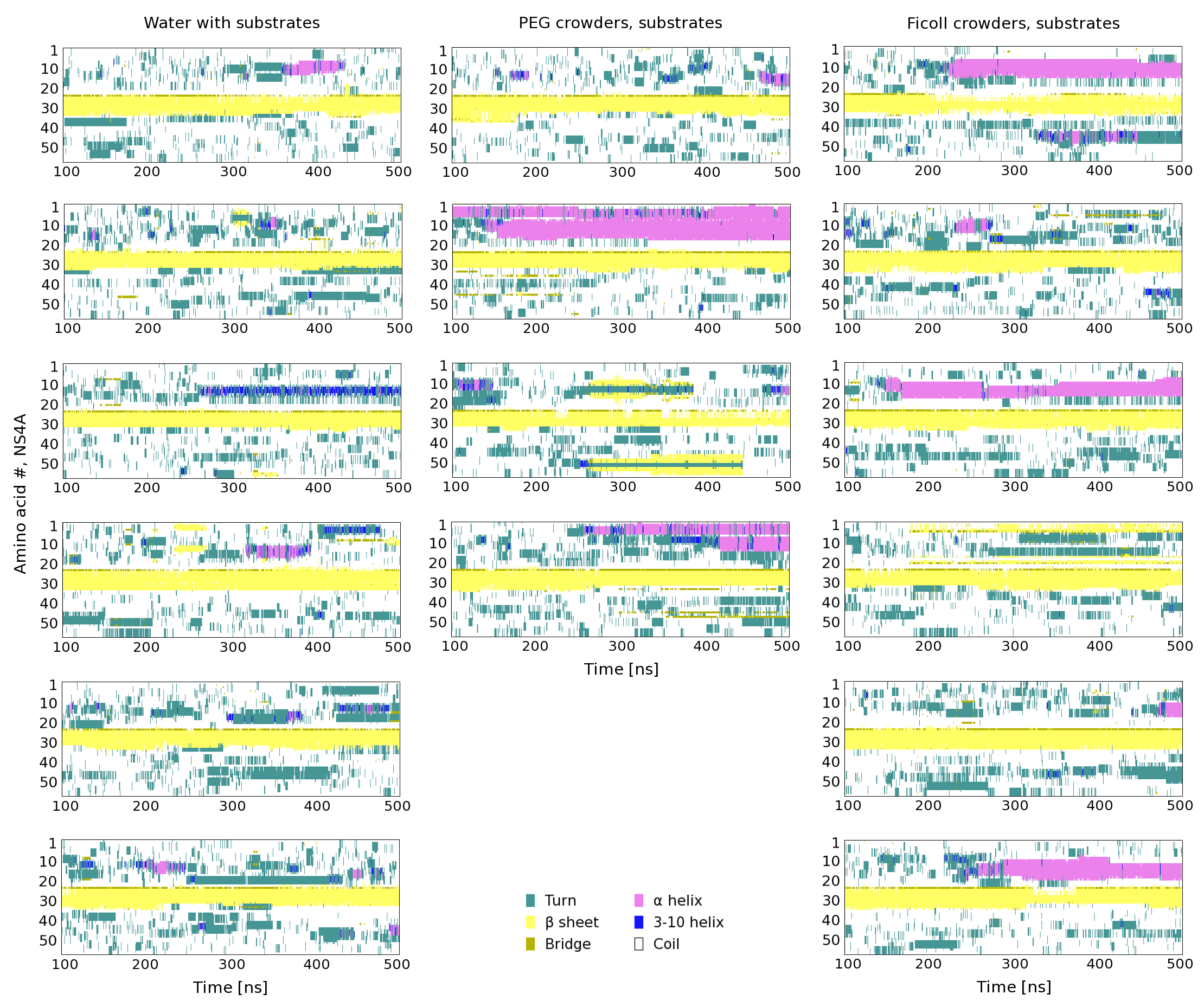

Supplement: S11 Fig — Helical conformations are marked in pink or blue, β-strands in yellow, and turns in cyan. Each graph shows data from one MD trajectory. (TIF) [file pcbi.1011054.s012.tif]

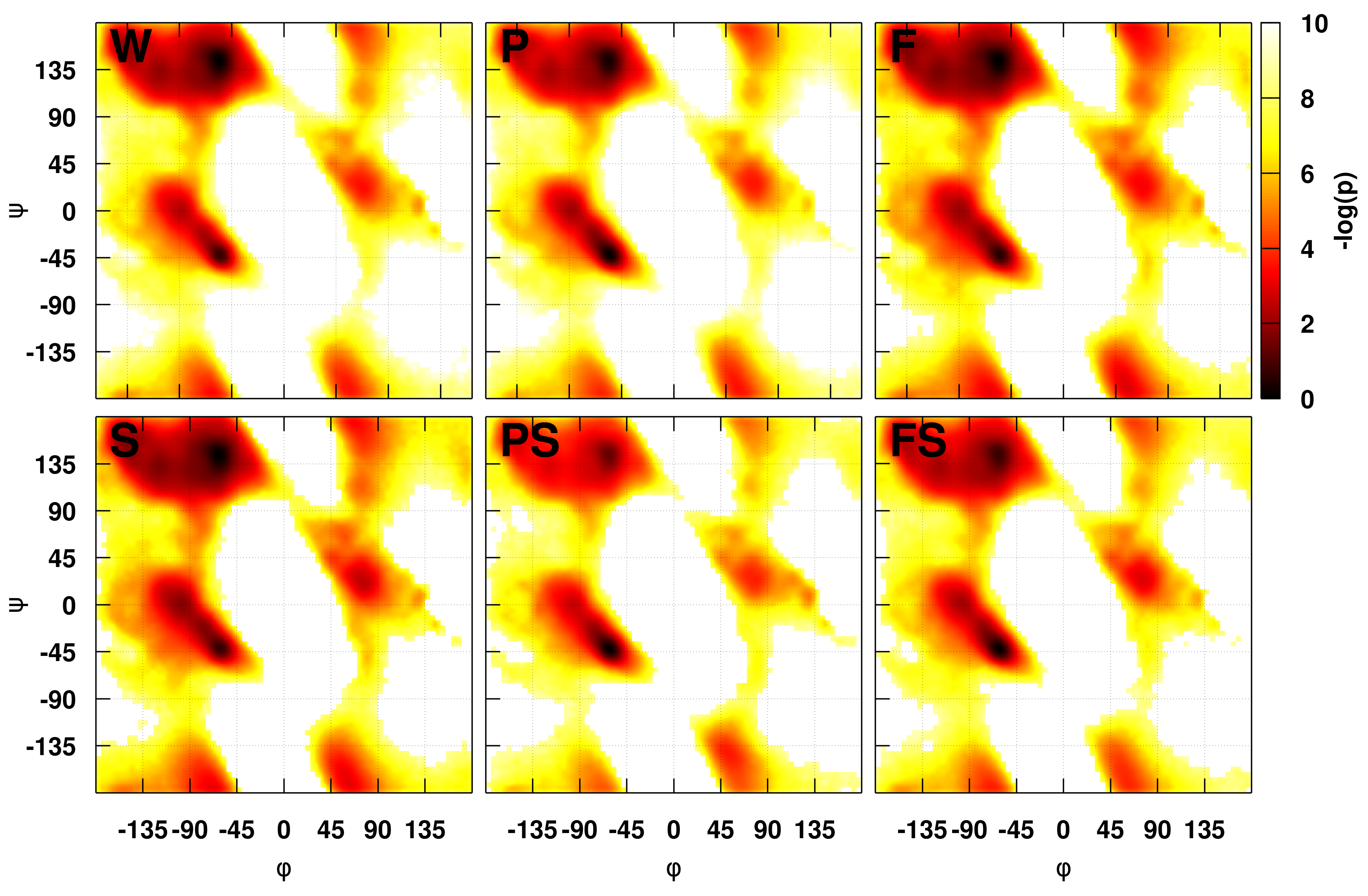

Supplement: S12 Fig — Results are shown from sampling in water (W), in the presence of PEG (P), in the presence of Ficoll (F), with substrates (S), with substrates in the presence of PEG (PS), and with substrates in the presence of Ficoll (FS). Colors indicate probabilities (-log(p)) according to the color bar. (TIF) [file pcbi.1011054.s013.tif]

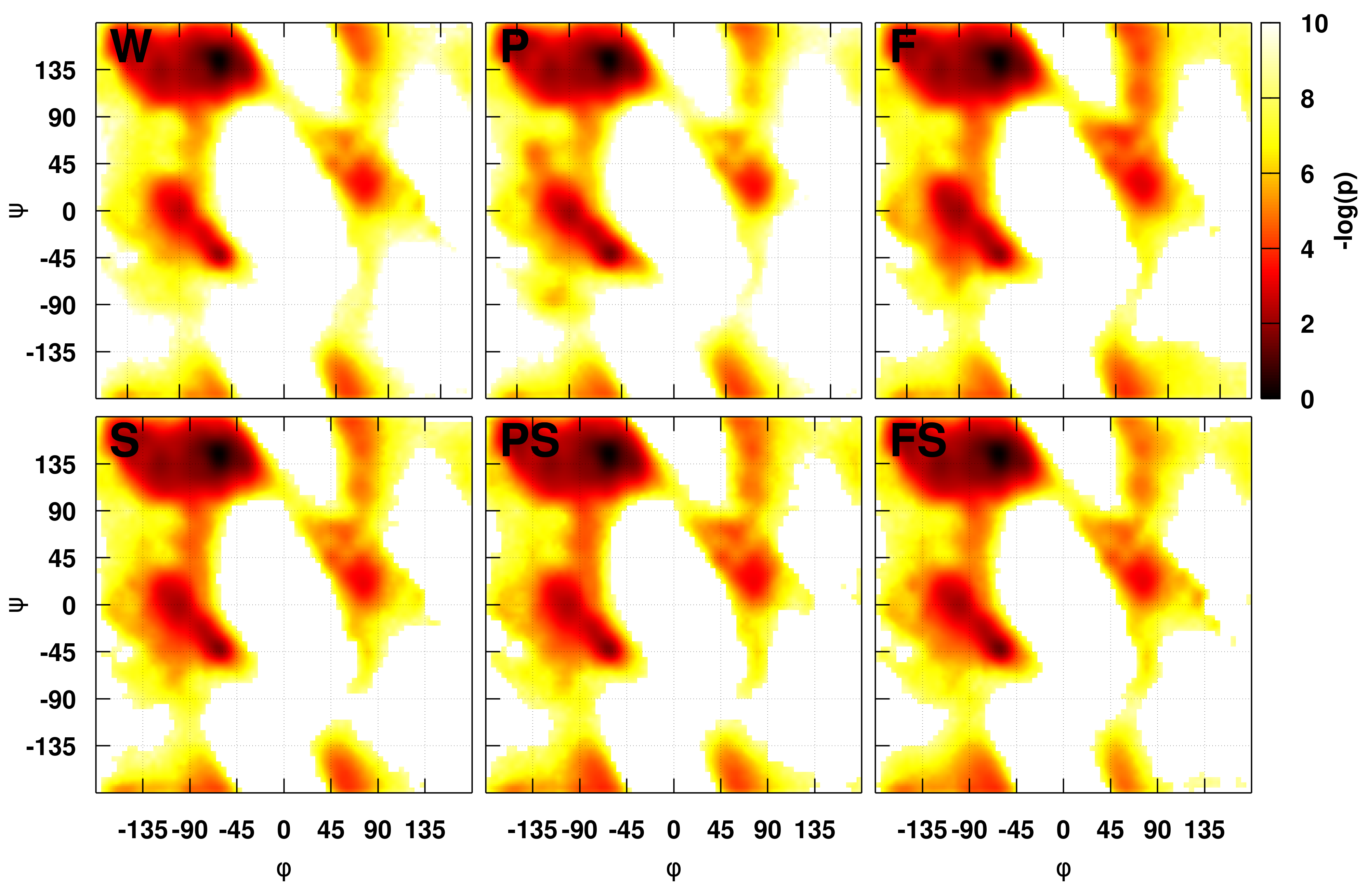

Supplement: S13 Fig — Results are shown from sampling in water (W), in the presence of PEG (P), in the presence of Ficoll (F), with substrates (S), with substrates in the presence of PEG (PS), and with substrates in the presence of Ficoll (FS). Colors indicate probabilities (-log(p)) according to the color bar. (TIF) [file pcbi.1011054.s014.tif]

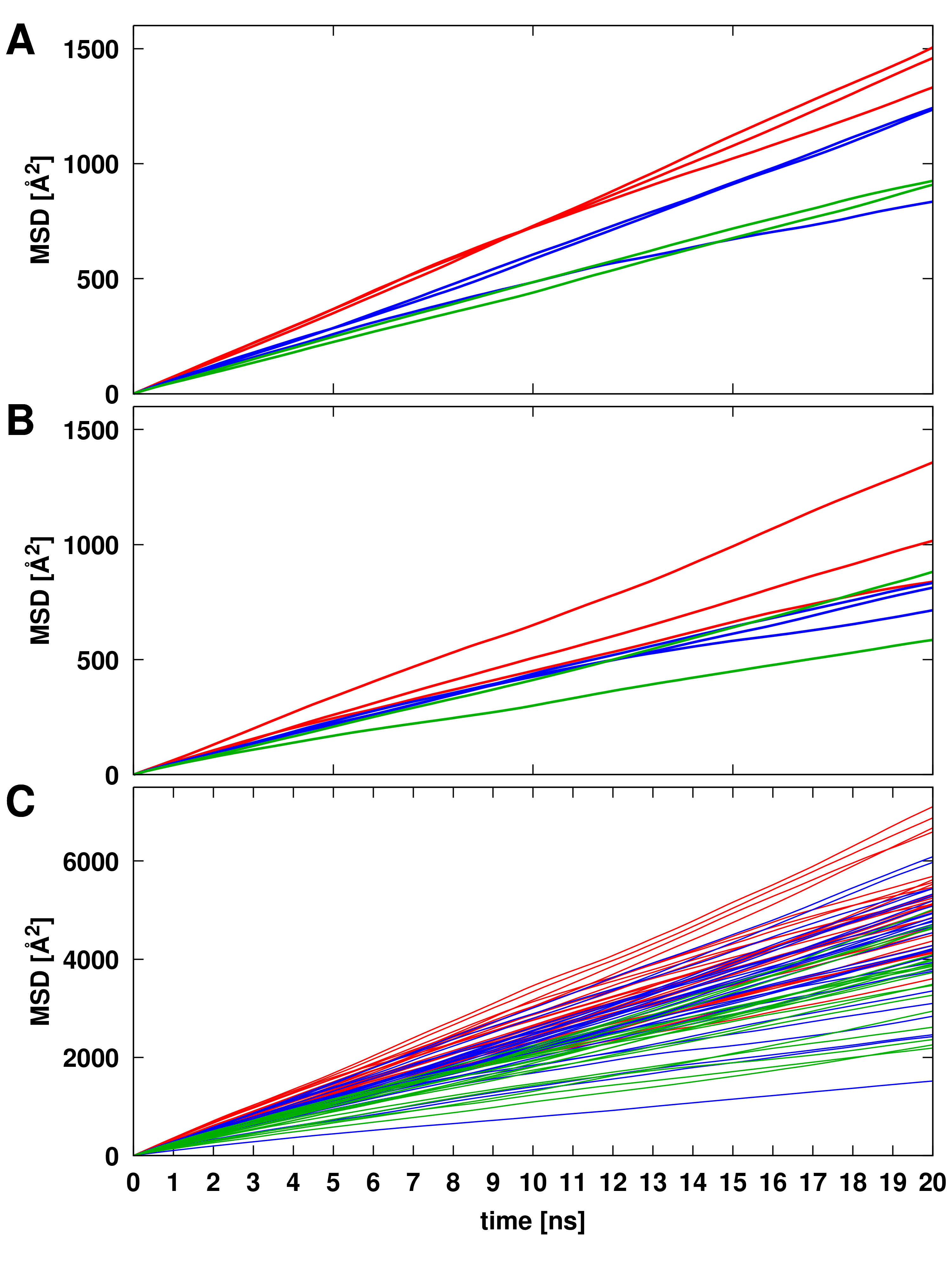

Supplement: S14 Fig — Results are shown without substrates (A), with substrates (B), and for substrates (C) in simulations with water (red), in the presence of PEG (green), and in the presence of Ficoll (blue). (TIF) [file pcbi.1011054.s015.tif]

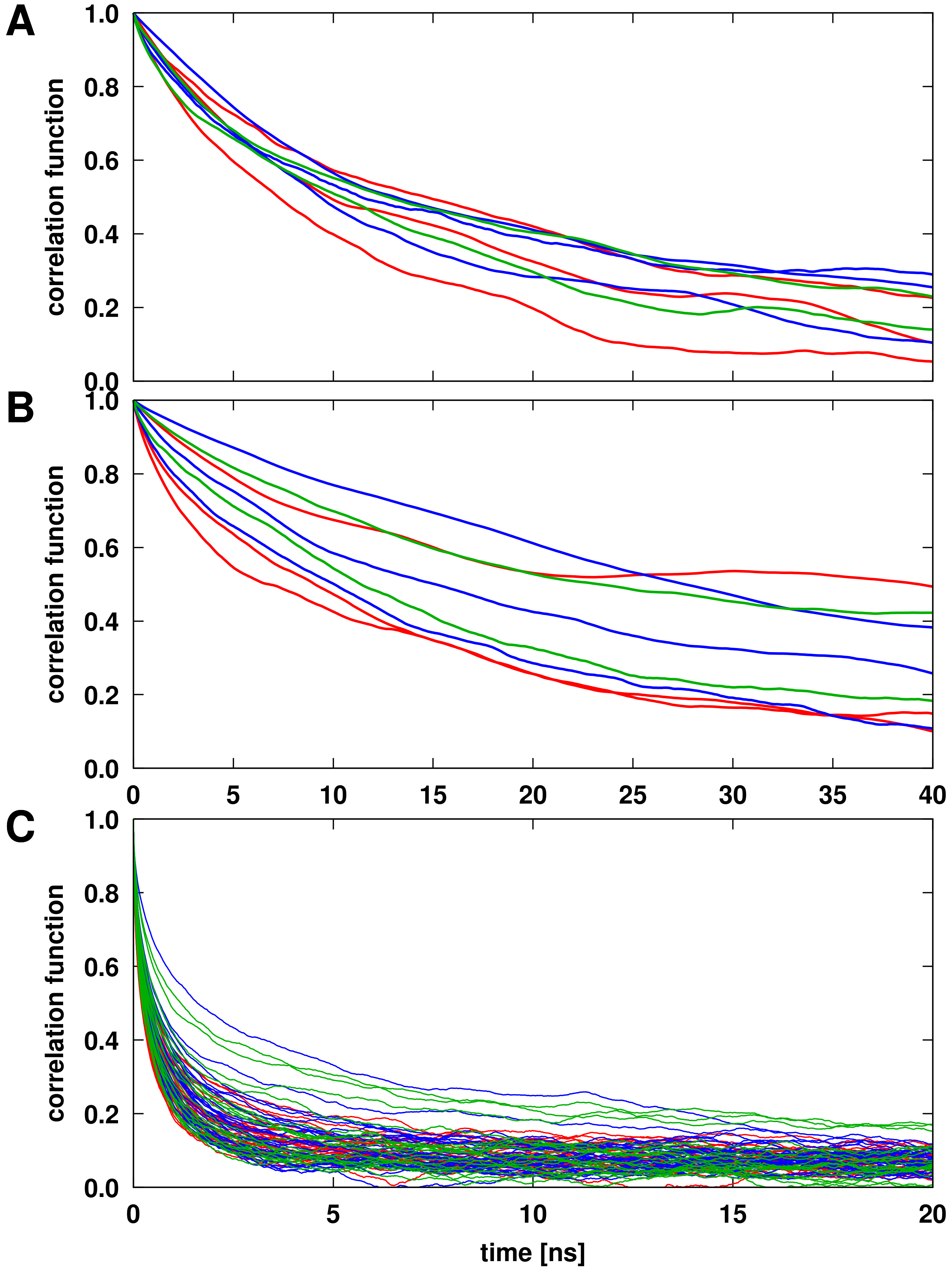

Supplement: S15 Fig — Results are shown without substrates (A), with substrates (B), and for substrates (C) in simulations with water (red), in the presence of PEG (green), and in the presence of Ficoll (blue). (TIF) [file pcbi.1011054.s016.tif]

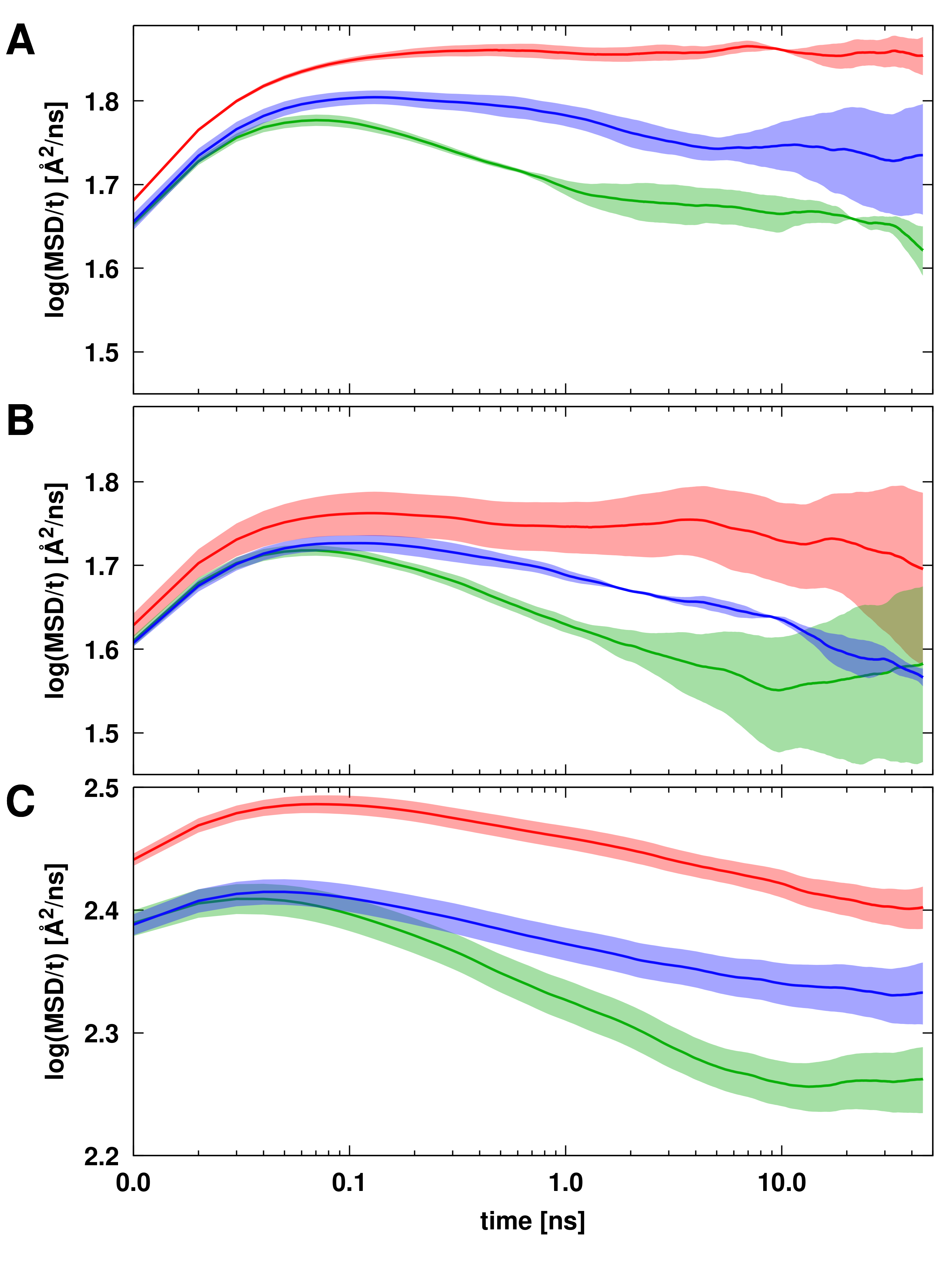

Supplement: S16 Fig — The underlying MSD curves are shown in S14 Fig. Results are shown for NS3/4A without substrates (A), with substrates (B), and for substrates (C) in simulations with water (red), in the presence of PEG (green), and in the presence of Ficoll (blue). Shaded areas indicate standard errors of the mean. (TIF) [file pcbi.1011054.s017.tif]

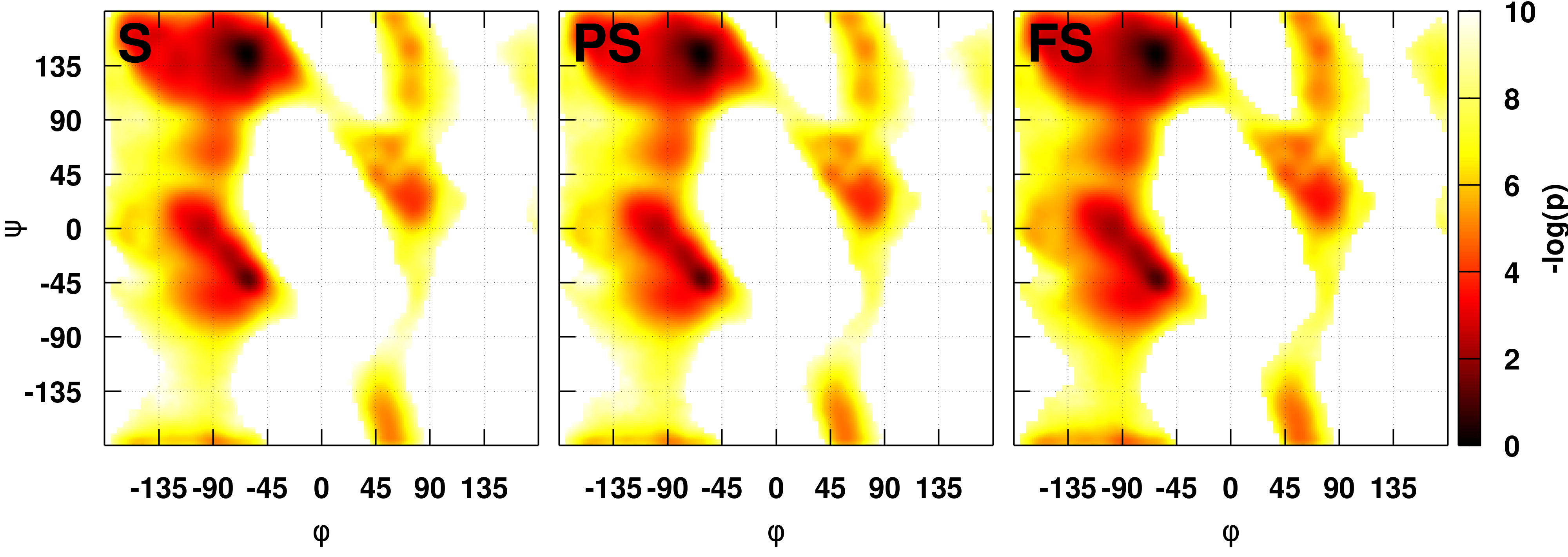

Supplement: S17 Fig — Results from sampling substrates with NS3/4A in water (S), in the presence of PEG (PS), and in the presence of Ficoll (FS). Colors indicate probabilities (-log(p)) according to the color bar. (TIF) [file pcbi.1011054.s018.tif]

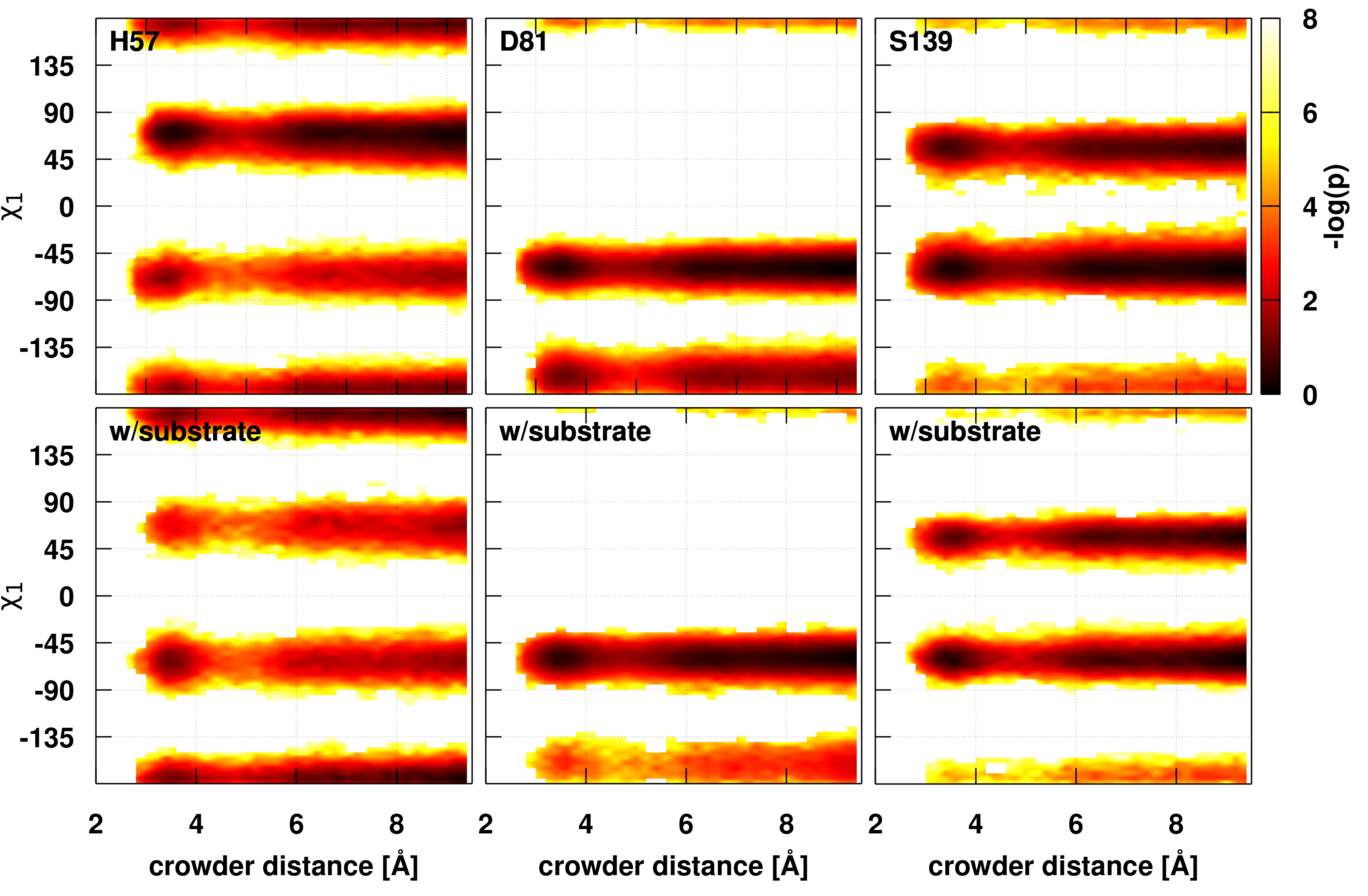

Supplement: S18 Fig — The distance was calculated to any of active site residues in simulations without (top row) and with (bottom row) substrates. In the crystal structure (4JMY), the values of the χ1 torsion are 76.6° for H57, -161.4° for D81, and -82.6° for S139. Colors indicate probabilities (-log(p)) according to the color bar. (TIF) [file pcbi.1011054.s019.tif]

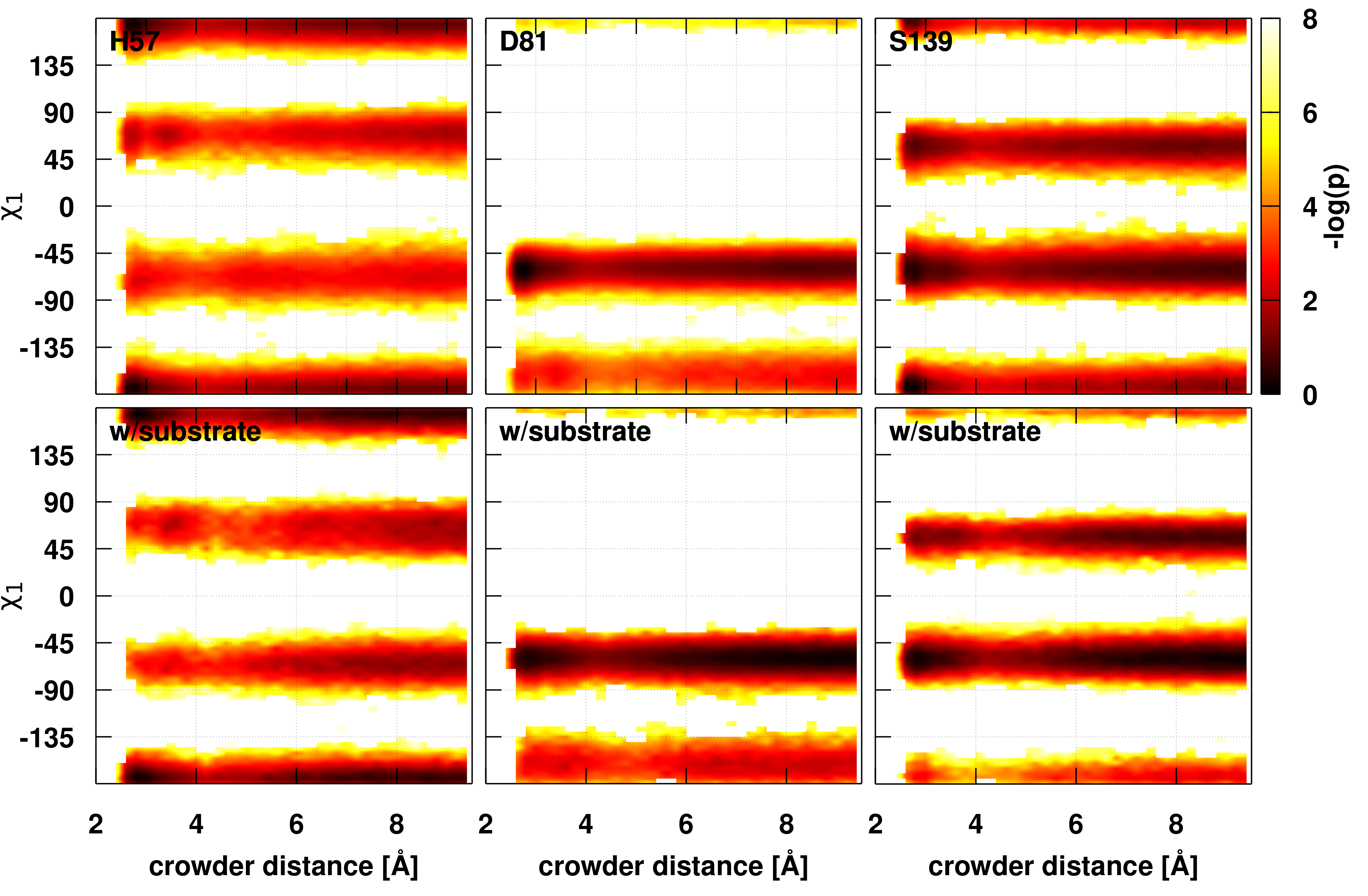

Supplement: S19 Fig — See S18 Fig for additional details. (TIF) [file pcbi.1011054.s020.tif]

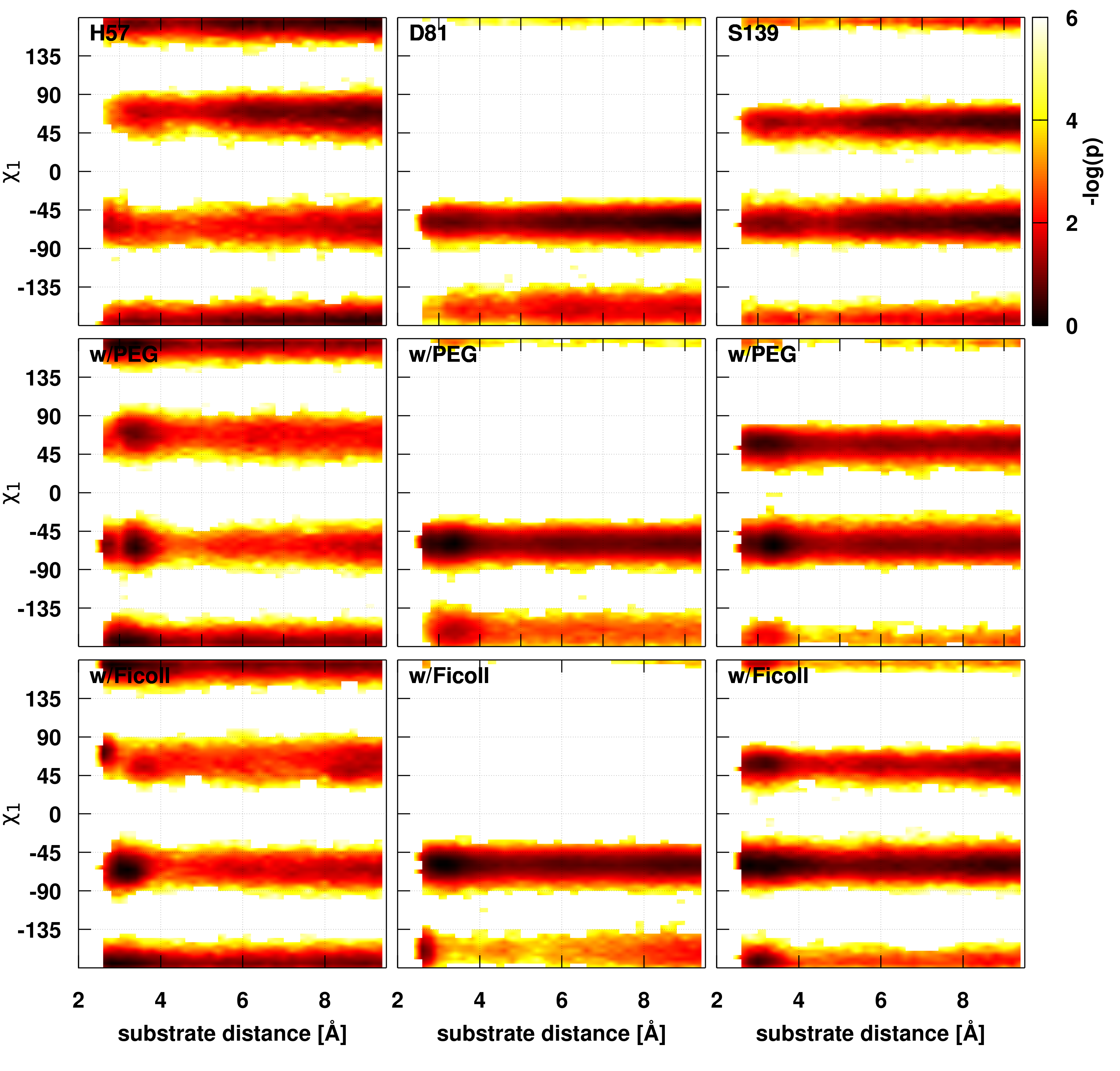

Supplement: S20 Fig — Results are shown for simulations with only substrate (top row) and with PEG (middle row) or Ficoll (bottom row) crowders as in S18 Fig. (TIF) [file pcbi.1011054.s021.tif]

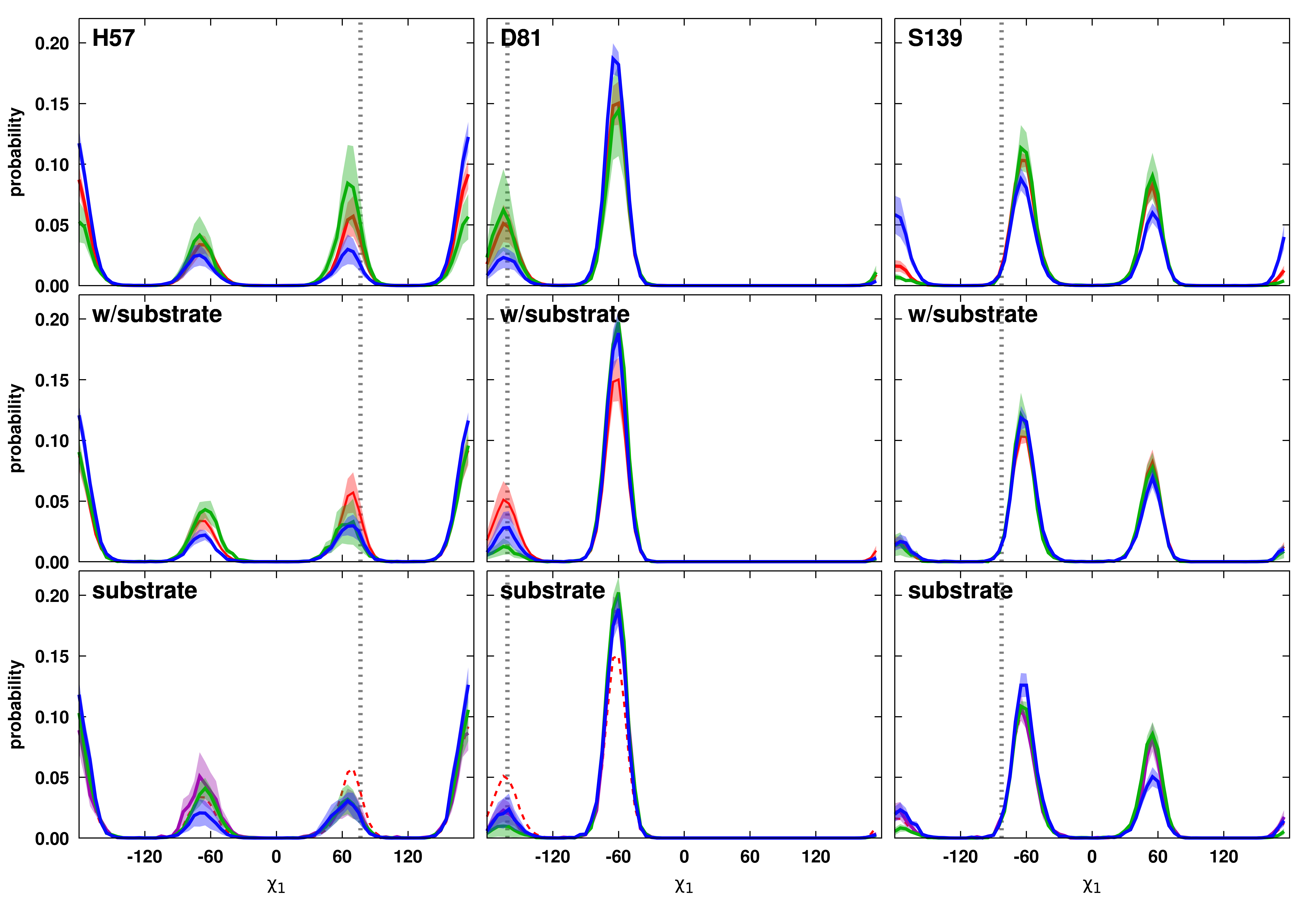

Supplement: S21 Fig — Contacts were defined as a minimum distance of 5 Å. Results for simulations with water (red) or only crowders (PEG: green, Ficoll: blue) are shown in the top row. Results for simulations with substrate but based on close crowder contacts are shown in the middle row. Results based on substrates being in close contact are shown in the bottom row. In the bottom row, the results from the simulations with only substrates are shown in purple. The dilute water distributions are shown as a dashed line for reference. Shaded areas indicate standard errors. (TIF) [file pcbi.1011054.s022.tif]

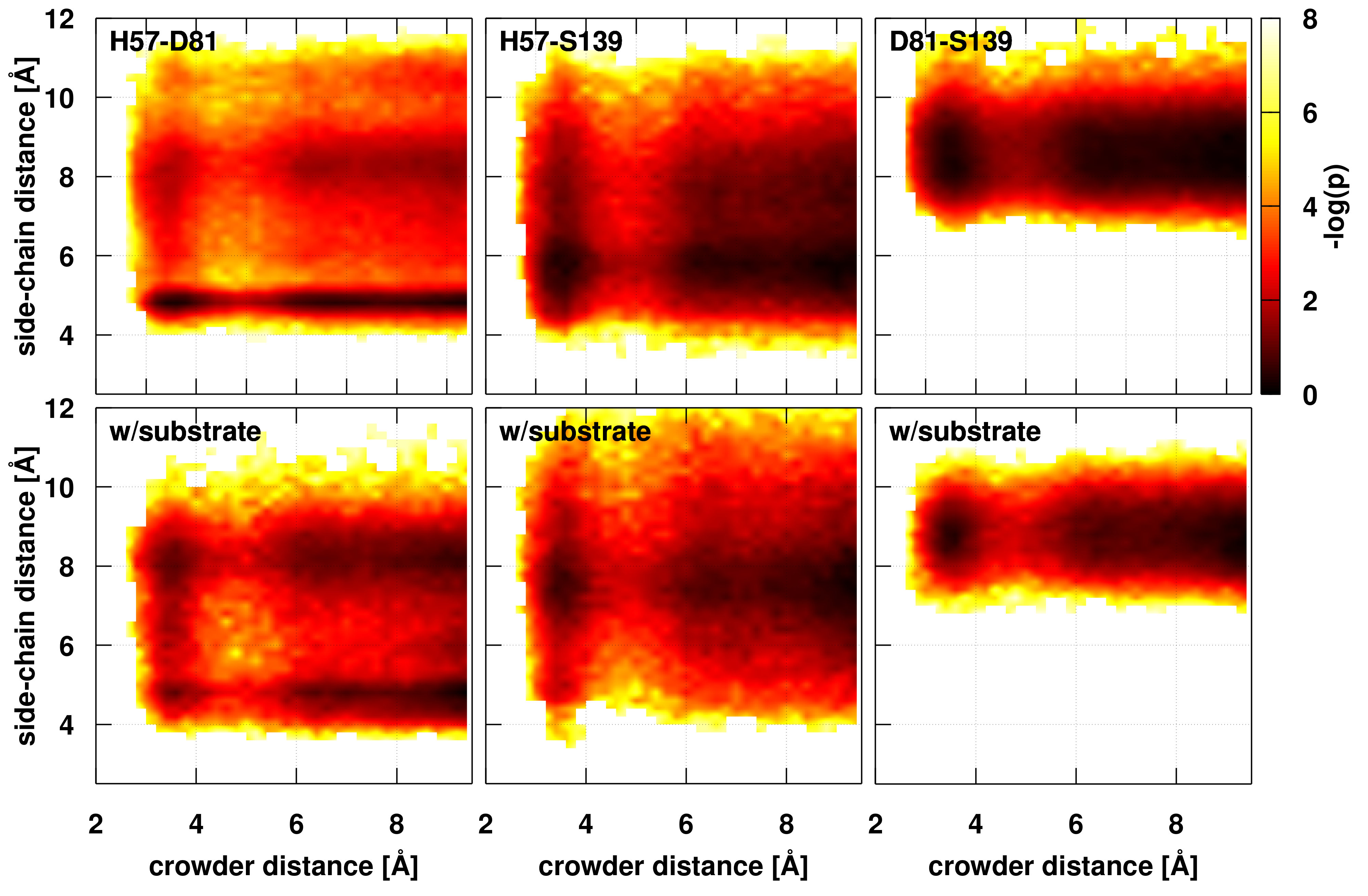

Supplement: S22 Fig — See S18 Fig for further details. In the crystal structure (PDB ID: 4JMY), the distances are 4.45 Å for H57-D81, 4.69 Å for H57-S139, and 7.37 Å for D81-S139. (TIF) [file pcbi.1011054.s023.tif]

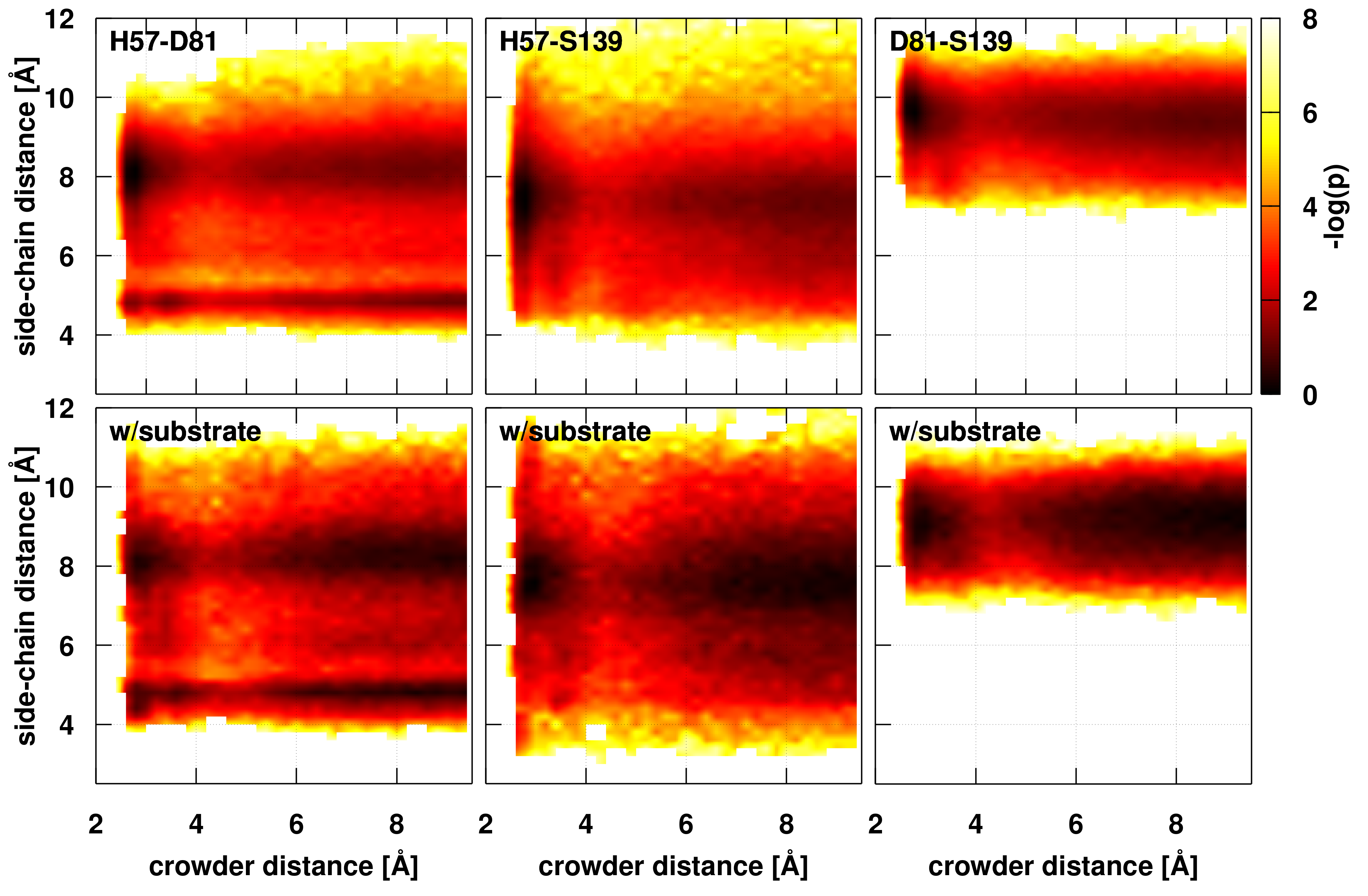

Supplement: S23 Fig — See S18 and S22 Figs for further details. (TIF) [file pcbi.1011054.s024.tif]

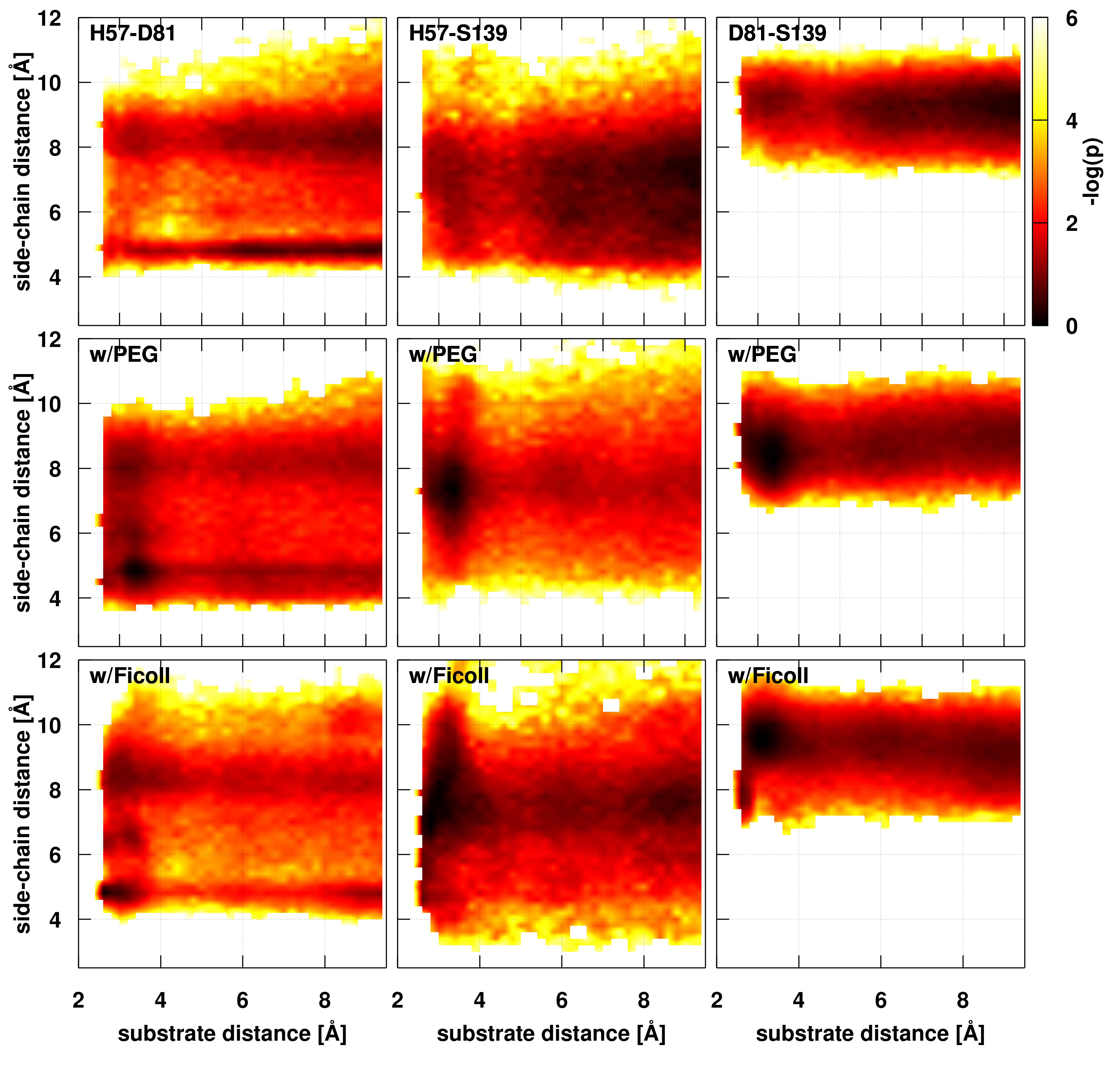

Supplement: S24 Fig — Results are shown for simulations with only substrates (top row) and with PEG (middle row) or Ficoll (bottom row) crowders as in S22 Fig. (TIF) [file pcbi.1011054.s025.tif]

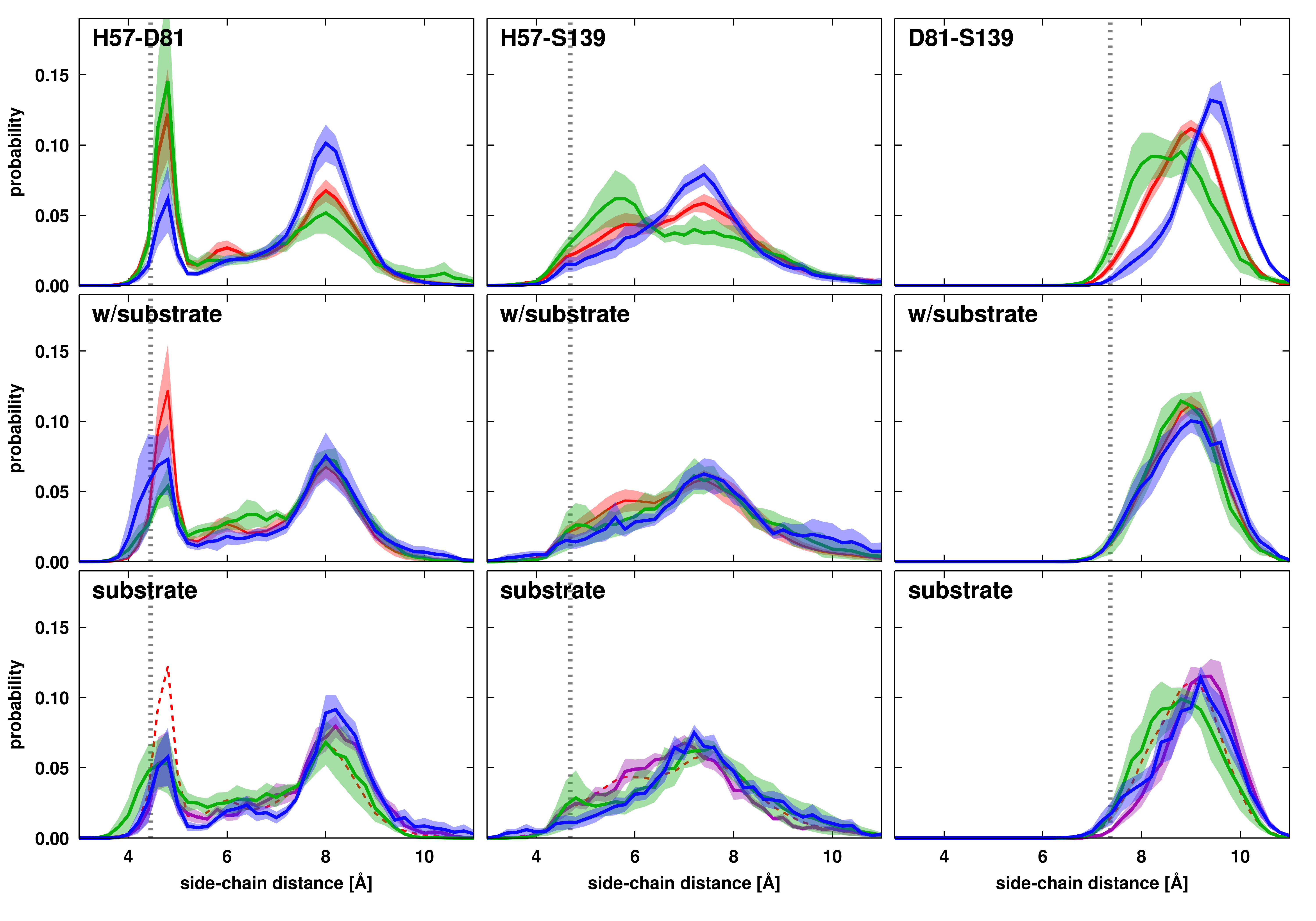

Supplement: S25 Fig — See S21 Fig for further details. (TIF) [file pcbi.1011054.s026.tif]

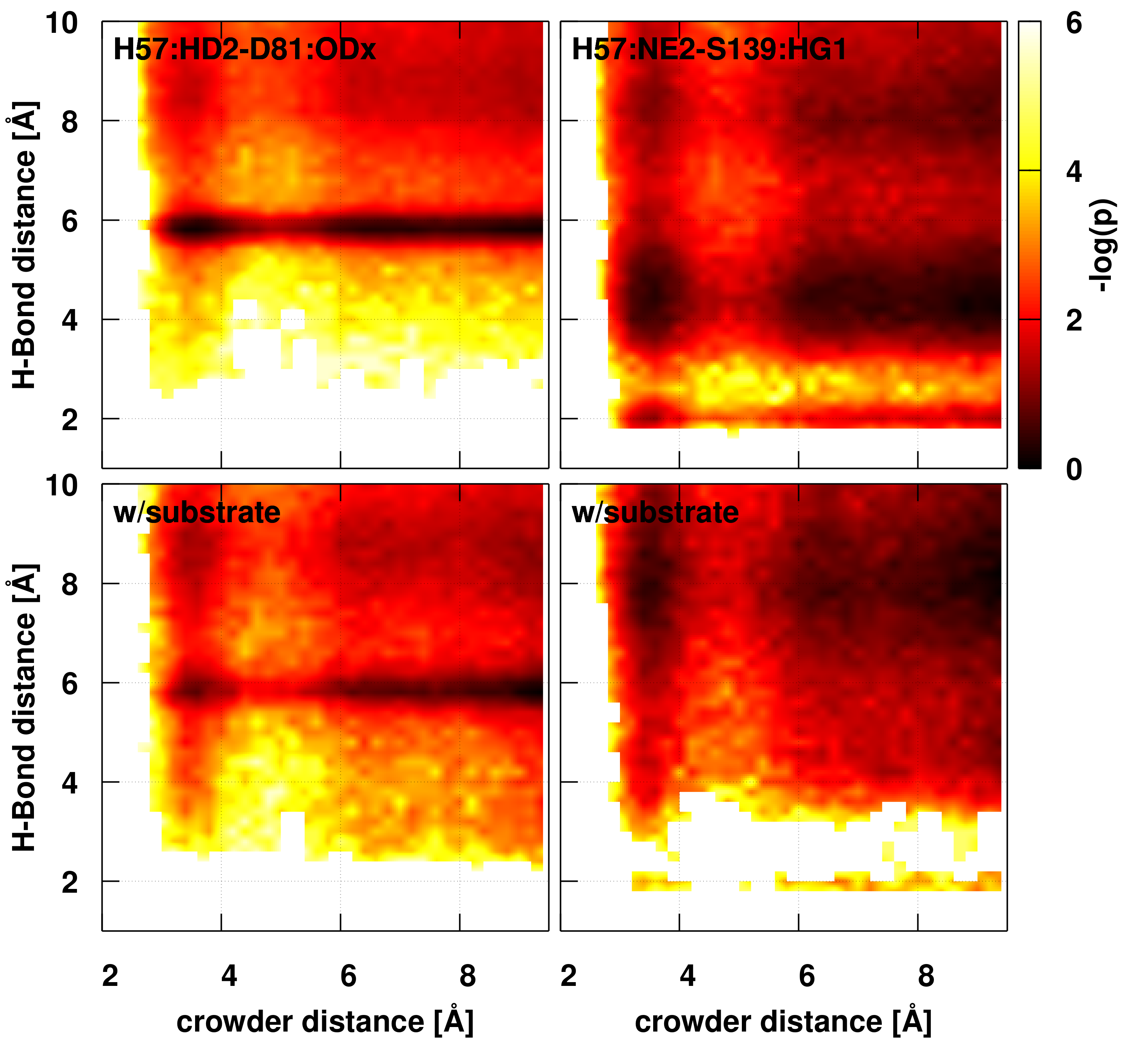

Supplement: S26 Fig — Results are shown for simulations without (top row) and with (bottom row) substrates. Colors indicate probabilities (-log(p)) according to the color bar. (TIF) [file pcbi.1011054.s027.tif]

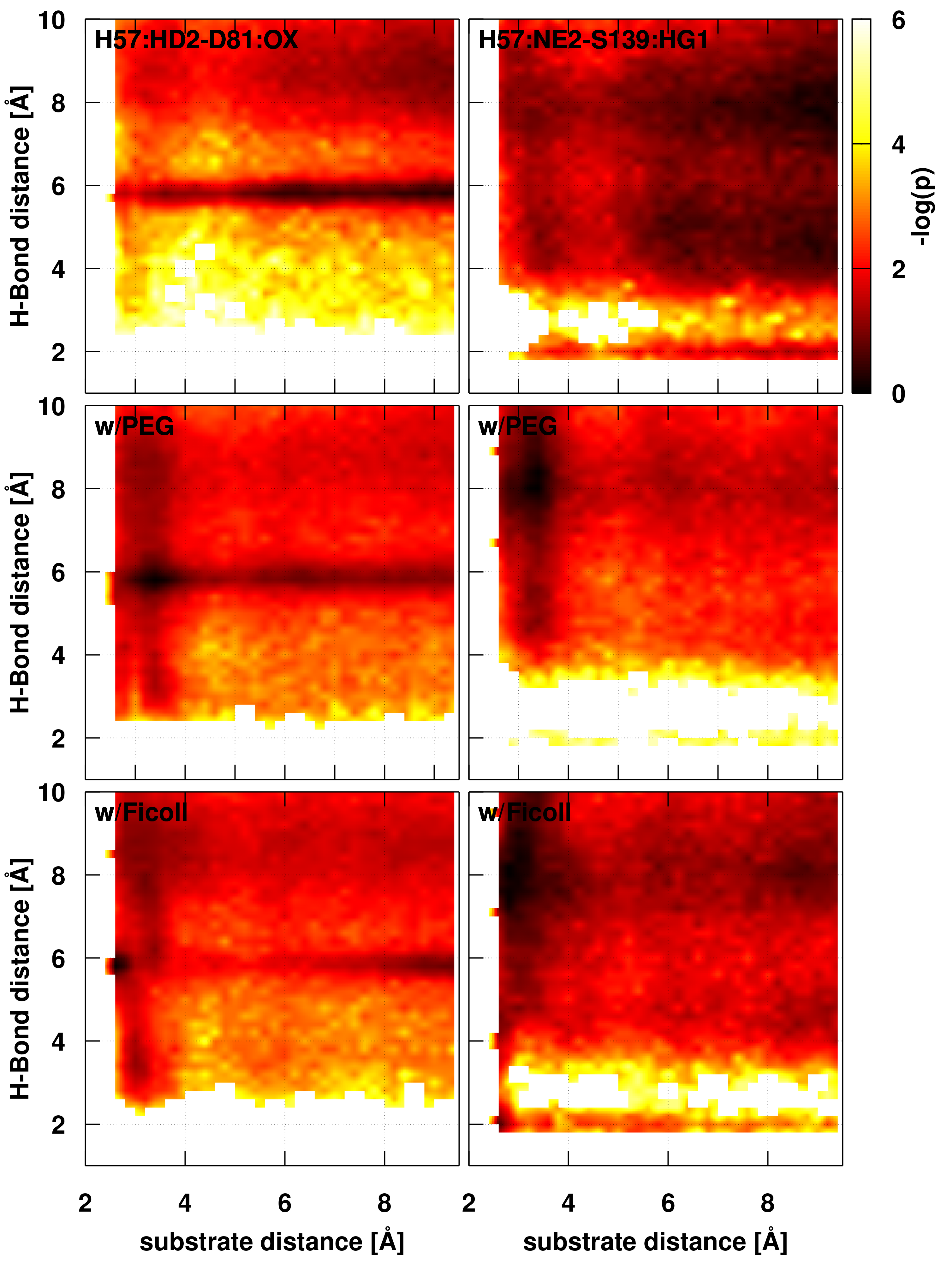

Supplement: S28 Fig — Results are shown for simulations with only substrate (top row) and with PEG (middle row) or Ficoll (bottom row) crowder as in S26 Fig. (TIF) [file pcbi.1011054.s029.tif]

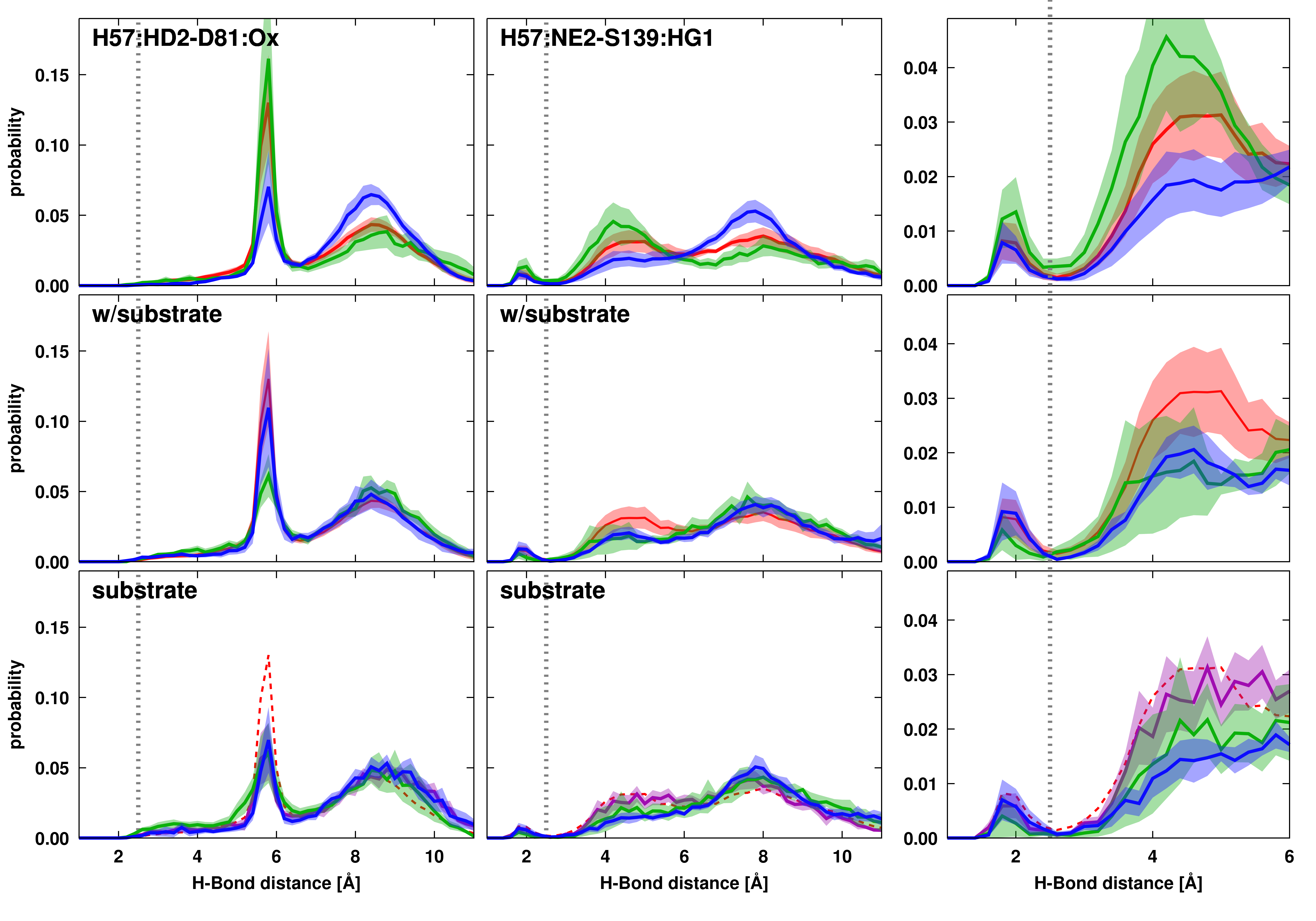

Supplement: S29 Fig — See S21 Fig for further details. The right-most column zooms in on the shorter-distance region of the data shown in the middle column. (TIF) [file pcbi.1011054.s030.tif]

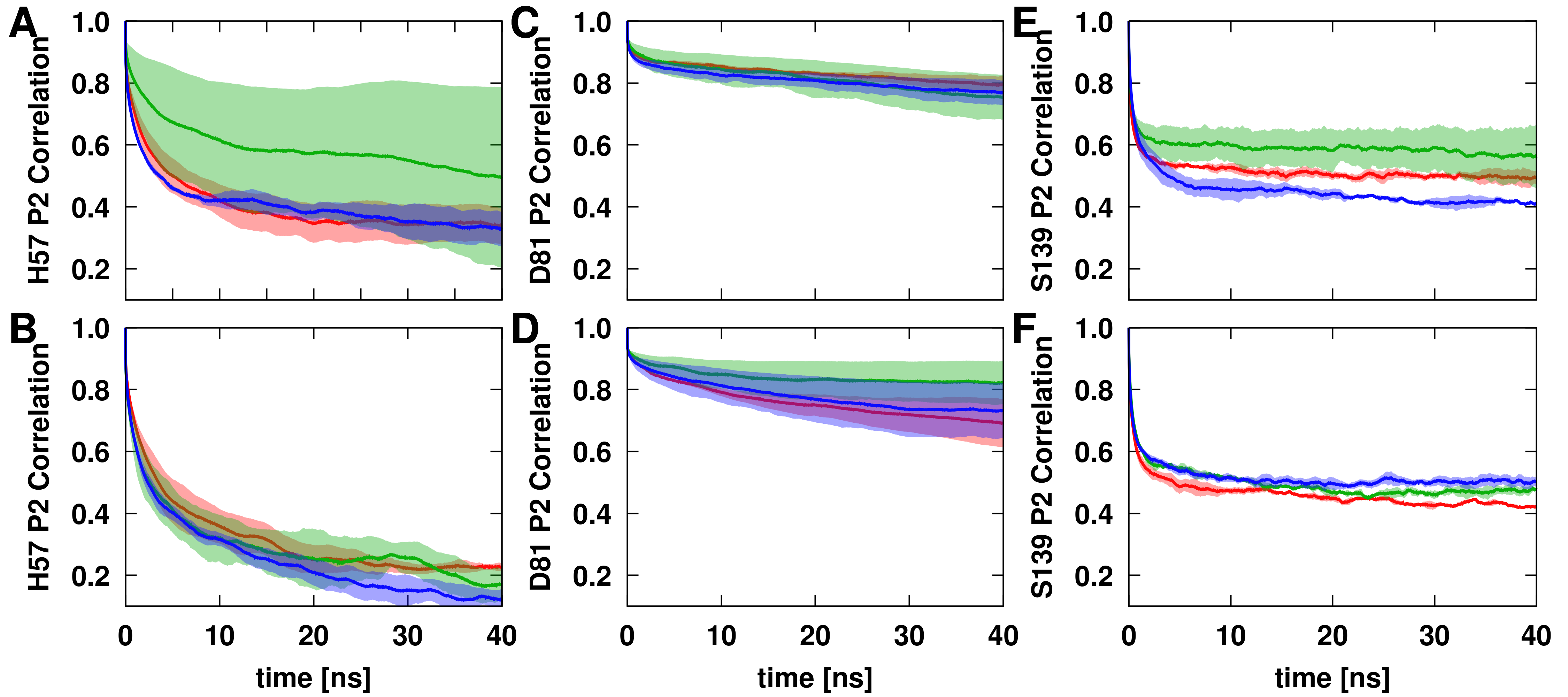

Supplement: S30 Fig — Fluctuations were calculated based on the Cα-Nε vector (H57, A/B), the Cα-Cγ vector (D81, C/D), and the Cα-Oγ vector (S139, E/F) after superposition of the overall NS3 structure to a common reference in water (red) or in the presence of Ficoll (blue) or PEG (green) crowder without (A/C/E) and with (B/D/F) substrates. The correlation function was calculated as the second-order Legendre polynomial from the inner dot product of the orientational vector at different time points. Solid lines show simulation averages, standard errors are indicated by the shaded area. Results from double exponential fits to the correlation functions are given in S8 Table. Only trajectories with the reduced friction were used in this analysis (S1 Table). (TIF) [file pcbi.1011054.s031.tif]
