# Supplementary material for: Varying molecular interactions explain aspects of crowder-dependent enzyme function of a viral protease
Source: PLoS Comput Biol. 2023 Apr 25;19(4):e1011054. doi: 10.1371/journal.pcbi.1011054 (PMC10162569; doi:10.1371/journal.pcbi.1011054)
Supplement: S1 Table — (PDF) [file pcbi.1011054.s032.pdf]

S1 Table Simulations of NS3/4A under different conditions analyzed in this study.

System	Time[2] [ns]	Frames[3]	Atoms	PEG	Ficoll	Substr.	H_2O	Na^+	Cl^-	Box[4] [$Å^3$]
**W1**	555	11108	128726	0	0	0	41718	20	22	108.5 x 108.5 x 108.5
**W2**	576	11522	128726	0	0	0	41718	20	22	108.5 x 108.5 x 108.5
**W1x[1]**	502	50200	128726	0	0	0	41718	20	22	108.7 x 108.7 x 108.7
**W2x[1]**	502	50200	128726	0	0	0	41718	20	22	108.7 x 108.7 x 108.7
**W3x[1]**	502	50200	128726	0	0	0	41718	20	22	108.7 x 108.7 x 108.7
**P1**	567	11348	258455	130	0	0	76031	25	27	142.9 x 132.4 x 132.2
**P2**	500	50000	258455	130	0	0	76031	25	27	144.2 x 133.5 x 133.4
**P3**	500	50000	258455	130	0	0	76031	25	27	144.2 x 133.5 x 133.4
**P1x[1]**	502	50200	258455	130	0	0	76031	25	27	144.2 x 133.6 x 133.4
**P2x[1]**	502	50200	258455	130	0	0	76031	25	27	144.2 x 133.6 x 133.4
**F1**	502	50200	260298	0	110	0	78084	37	39	134.7 x 135.7 x 140.4
**F2**	502	50200	256218	0	110	0	76724	37	39	136.7 x 135.7 x 136.1
**F2**	502	50200	260891	0	110	0	78281	38	40	137.1 x 137.3 x 136.6
**F1x[1]**	502	50200	260298	0	110	0	78084	37	39	134.7 x 135.7 x 140.5
**F2x[1]**	502	50200	256218	0	110	0	76724	37	39	136.7 x 135.7 x 136.1
**F3x[1]**	502	50200	260891	0	110	0	78281	38	40	137.2 x 137.4 x 136.6
**S1**	528	10556	147865	0	0	10	47647	61	23	103.3 x 122.5 x 113.2
**S2**	666	13321	108274	0	0	10	34454	55	17	104.2 x 96.2 x 104.6
**S3**	548	10951	103337	0	0	10	32809	54	16	99.3 x 97.8 x 102.9
**S1x[1]**	502	50200	147865	0	0	10	47647	61	23	104.5 x 123.5 x 114.3
**S2x[1]**	502	50200	108274	0	0	10	34454	55	17	105.1 x 97.2 x 105.5
**S3x[1]**	502	50200	103337	0	0	10	32809	54	16	100.2 x 98.8 x 103.9
**PS1**	562	11232	246934	130	0	10	71736	72	34	143.1 x 132.7 x 125.6
**PS2**	505	10103	241244	130	0	10	69840	71	33	145.8 x 134.1 x 119.2
**PS1x[1]**	502	50200	246934	130	0	10	71736	72	34	144.4 x 133.9 x 126.8
**PS2x[1]**	502	50200	241244	130	0	10	69840	71	33	147.1 x 135.3 x 120.3
**FS1**	518	10364	244759	0	110	10	72457	73	35	131.7 x 132.5 x 137.2
**FS2**	549	10975	232827	0	110	10	68481	71	33	133.0 x 130.7 x 131.0
**FS3**	537	10740	235800	0	110	10	69472	71	33	132.6 x 131.8 x 132.0
**FS1x[1]**	502	50200	244759	0	110	10	72457	73	35	132.0 x 132.7 x 137.5
**FS2x[1]**	502	50200	232827	0	110	10	68481	71	33	133.2 x 130.9 x 131.2
**FS3x[1]**	502	50200	235800	0	110	10	69472	71	33	132.9 x 132.0 x 132.2

[1]Simulations with a reduced Langevin friction coefficient of 0.01 ps^{-1}
[2]Total simulation time.
[3]Total number of snapshots saved during simulation.
[4]Simulation-averaged
