# Supplementary material for: Varying molecular interactions explain aspects of crowder-dependent enzyme function of a viral protease
Source: PLoS Comput Biol. 2023 Apr 25;19(4):e1011054. doi: 10.1371/journal.pcbi.1011054 (PMC10162569; doi:10.1371/journal.pcbi.1011054)
Supplement: S2 Table — (PDF) [file pcbi.1011054.s033.pdf]

S2 Table Average Cα coordinate root mean square deviations of NS3/4A

System	NS3/4A RMSD [Å]
**Water**	1.599 (0.086)
**PEG**	1.639 (0.050)
**Ficoll**	1.647 (0.101)
**Substrate**	1.578 (0.067)
**PEG/Substrate**	1.697 (0.113)
**Ficoll/Substrate**	1.480 (0.073)

RMSD values were calculated after optimal superposition with respect to the experimental structure (PDB ID: 4JMY). Only the part of NS4A resolved in the PDB structure was considered. Averages based on all trajectories for a given system with standard errors of the mean in parentheses.
