# Supplementary material for: Varying molecular interactions explain aspects of crowder-dependent enzyme function of a viral protease
Source: PLoS Comput Biol. 2023 Apr 25;19(4):e1011054. doi: 10.1371/journal.pcbi.1011054 (PMC10162569; doi:10.1371/journal.pcbi.1011054)
Supplement: S3 Table — (PDF) [file pcbi.1011054.s034.pdf]

S3 Table Average radius of gyration for NS3, NS4A, substrate, and crowders from heavy atoms

System	NS3 R_g [Å]	NS4A R_g [Å]	Substrate R_g [Å]	Ficoll R_g [Å]	PEG R_g [Å]
**Water**	16.22 (0.04)	24.49 (1.49)			
**PEG**	16.23 (0.03)	25.98 (0.94)			12.56 (0.03)
**Ficoll**	16.27 (0.02)	24.17 (1.15)		8.09 (0.001)	
**Substrate**	16.25 (0.02)	25.77 (0.53)	7.81 (0.06)		
**PEG/Substrate**	16.21 (0.03)	24.59 (1.82)	7.68 (0.07)		12.57 (0.01)
**Ficoll/Substrate**	16.21 (0.02)	26.78 (1.27)	7.64 (0.02)	8.08 (0.002)	

Averages based on all trajectories for a given system with standard errors of the mean in parentheses.
