# Supplementary material for: Varying molecular interactions explain aspects of crowder-dependent enzyme function of a viral protease
Source: PLoS Comput Biol. 2023 Apr 25;19(4):e1011054. doi: 10.1371/journal.pcbi.1011054 (PMC10162569; doi:10.1371/journal.pcbi.1011054)
Supplement: S4 Table — (PDF) [file pcbi.1011054.s035.pdf]

S4 Table Average secondary structure content for the NS4A and substrate calculated based on VMD Timeline results (the helix % includes both α and 3_{10} helix structures).

System	NS4A		Substrate	
	**helix [%]**	**β-sheet [%]**	**helix [%]**	**β-sheet [%]**
**Water**	1.57 (0.91)	2.24 (0.22)		
**Ficoll**	1.35 (0.95)	2.51 (0.10)		
**PEG**	5.64 (1.31)	2.03 (0.10)		
**Substrate**	1.75 (0.28)	2.66 (0.29)	3.45 (1.31)	0
**Ficoll/Substrate**	5.58 (1.99)	3.19 (1.00)	5.47 (1.09)	0
**PEG/Substrate**	8.30 (4.51)	2.62 (0.33)	3.04 (0.94)	0

Averages based on all trajectories for a given system with standard errors of the mean in parentheses.
