# Supplementary material for: Varying molecular interactions explain aspects of crowder-dependent enzyme function of a viral protease
Source: PLoS Comput Biol. 2023 Apr 25;19(4):e1011054. doi: 10.1371/journal.pcbi.1011054 (PMC10162569; doi:10.1371/journal.pcbi.1011054)
Supplement: S5 Table — (PDF) [file pcbi.1011054.s036.pdf]

S5 Table Single- and double-exponential fits to rotational correlation functions for enzyme and substrates.

	**NS3/4A**				**Substrate**			
	**τ [ns]**		**s**	**χ^2**	**τ [ns]**		**s**	**χ^2**
**Water**	19.13		0.89	2.71				
**PEG**	23.34		0.85	3.43				
**Ficoll**	25.23		0.85	6.95				
**Substrate**	28.20		0.80	8.63	23.81		0.19	13.97
**PEG/Substrate**	31.48		0.88	6.62	19.45		0.22	15.10
**Ficoll/Substrate**	29.21		0.90	1.68	20.01		0.23	13.90
	**τ_s [ns]**	**τ_f [ns]**	**s_r**		**τ_s [ns]**	**τ_f [ns]**	**s_r**	
**Water**	23.88	4.22	0.73	0.52				
**PEG**	39.08		0.72	0.50				
**Ficoll**	46.72	6.89	0.59	0.06				
**Substrate**	48.98	5.32	0.53	0.95	61.94	0.52	0.11	1.36
**PEG/Substrate**	4285.8	13.50	0.26	0.25	46.44	0.57	0.14	1.31
**Ficoll/Substrate**	34.42	4.78	0.79	0.12	52.23	0.60	0.12	1.80

Time decays and weight of slow-time scale component (see Methods) from single- (top) or double-exponential (bottom) fits to combined rotational correlation functions.
