# Supplementary material for: Varying molecular interactions explain aspects of crowder-dependent enzyme function of a viral protease
Source: PLoS Comput Biol. 2023 Apr 25;19(4):e1011054. doi: 10.1371/journal.pcbi.1011054 (PMC10162569; doi:10.1371/journal.pcbi.1011054)
Supplement: S6 Table — (PDF) [file pcbi.1011054.s037.pdf]

S6 Table NS3-crowder contact life-times from single- and double-exponential fits to contact survival decays

	**τ [ns]**		**a**	χ^2
**PEG**	4.97 (0.77)		0.53 (0.023)	2.671
**PEG w/substrate**	5.01 (0.25)		0.54 (0.008)	2.333
**Ficoll**	3.27 (0.22)		0.60 (0.011)	1.580
**Ficoll w/substrate**	3.55 (0.23)		0.61 (0.008)	1.565
**Substrate**	19.00 (1.35)		0.60 (0.007)	4.127
**Substrate w/PEG**	53.63 (2.34)		0.66 (0.074)	4.011
**Substrate w/Ficoll**	18.87 (5.28)		0.53 (0.030)	3.679
	**τ_1 [ns]**	**τ_2 [ns]**	**a**	χ^2
**PEG**	0.28 (0.09)	6.71 (1.61)	0.58 (0.045)	0.851
**PEG w/substrate**	0.27 (0.03)	6.37 (0.41)	0.56 (0.002)	0.727
**Ficoll**	0.23 (0.02)	4.17 (0.23)	0.52 (0.025)	0.565
**Ficoll w/substrate**	0.24 (0.04)	4.43 (0.41)	0.50 (0.013)	0.557
**Substrate**	1.93 (1.78)	47.0 (28.4)	0.42 (0.008)	1.884
**Substrate w/PEG**	2.88 (0.93)	94.3 (5.8)	0.29 (0.050)	1.406
**Substrate w/Ficoll**	1.48 (1.44)	48.9 (35.1)	0.43 (0.052)	1.345

Averages over replicate trajectories with reduced friction coefficients. Standard errors are given in parentheses.
