# Supplementary material for: Varying molecular interactions explain aspects of crowder-dependent enzyme function of a viral protease
Source: PLoS Comput Biol. 2023 Apr 25;19(4):e1011054. doi: 10.1371/journal.pcbi.1011054 (PMC10162569; doi:10.1371/journal.pcbi.1011054)
Supplement: S7 Table — (PDF) [file pcbi.1011054.s038.pdf]

S7 Table Substrate-crowder contact life-times from double-exponential fits to contact survival decays

	τ_1 [ns]	τ_2 [ns]	a	χ^2
**PEG**	0.073 (0.002)	1.50 (0.05)	0.71 (0.000)	0.255
**Ficoll**	0.106 (0.001)	1.74 (0.03)	0.60 (0.001)	0.254

Averages over replicate trajectories with standard errors given in parentheses.
