# Supplementary material for: Varying molecular interactions explain aspects of crowder-dependent enzyme function of a viral protease
Source: PLoS Comput Biol. 2023 Apr 25;19(4):e1011054. doi: 10.1371/journal.pcbi.1011054 (PMC10162569; doi:10.1371/journal.pcbi.1011054)
Supplement: S8 Table — (PDF) [file pcbi.1011054.s039.pdf]

S8 Table Double-exponential fits to the combined orientational correlation functions of the H57, D81, and S139 active site side chains

	**H57**			**D81**			**S139**		
	**τ_1 [ns]**	**τ_2 [ns]**	**a**	**τ_1 [ns]**	**τ_2 [ns]**	**a**	**τ_1 [ns]**	**τ_2 [ns]**	**a**
**Water**	129.5	1.07	0.44	403.7	0.257	0.88	455.7	0.319	0.54
**PEG**	141.8	1.53	0.67	247.1	0.331	0.88	509.4	0.269	0.61
**Ficoll**	108.4	0.99	0.47	303.8	0.363	0.85	214.4	0.717	0.49
**Substrate**	55.2	1.86	0.41	211.2	1.205	0.83	209.7	0.447	0.50
**PEG/Substrate**	46.6	1.22	0.41	783.3	0.911	0.86	296.7	0.549	0.53
**Ficoll/Substrate**	28.7	1.06	0.45	263.4	0.945	0.84	521.9	0.497	0.53
