# Supplementary material for: Varying molecular interactions explain aspects of crowder-dependent enzyme function of a viral protease
Source: PLoS Comput Biol. 2023 Apr 25;19(4):e1011054. doi: 10.1371/journal.pcbi.1011054 (PMC10162569; doi:10.1371/journal.pcbi.1011054)
Supplement: S9 Table — (PDF) [file pcbi.1011054.s040.pdf]

S9 Table Cluster analysis for substrate binding near the active site in simulations without crowders

#	N[1]	cum%[2]	End2end[3] [Å]	cosθ[4]	substrates[5]
1	90	8.0	19.97 (0.38)	0.86 (0.008)	S10(58;58) S8(18;16) S3(5;4)
2	75	14.6	16.83 (0.48)	0.09 (0.031)	S8(24;9) S2 (23;12) S7(17;11) S4(7;4)
3	68	20.6	21.55 (0.27)	0.64 (0.025)	S6(39;37) S1(12;11) S1(12;12)
4	66	26.5	20.32 (0.34)	0.60 (0.022)	S6(24;12) S3(17;11) S2(11;5) S1(8;8)
5	57	31.5	21.23 (0.34)	0.27 (0.046)	S7(29;11) S1(21;18)
6	54	36.3	16.91 (0.56)	0.16 (0.060)	S8(22;10) S7(11;4) S1(8;7) S4(5;2)
7	44	40.2	16.00 (0.58)	0.63 (0.042)	S8(16;5) S3(9;5) S9(7;3)
8	42	43.9	15.55 (0.85)	-0.48 (0.045)	S8(24;13)
9	39	47.3	19.38 (0.58)	-0.38 (0.057)	S7(15;3) S6(8;5) S5(8;8)
10	37	50.6	20.72 (0.48)	0.60 (0.031)	S7(23;22) S10(7;3)
11	37	53.9	15.50 (0.36)	0.71 (0.043)	S8(27;26) S2(8;8)
12	36	57.1	14.17 (0.33)	-0.68 (0.046)	S1(29;14)
13	35	60.2	15.92 (0.57)	-0.16 (0.072)	S6(13;8) S8(11;10)
14	34	63.2	17.74 (0.89)	-0.74 (0.030)	S8(13;3) S6(10;7)
15	34	66.2	17.09 (0.66)	-0.77 (0.035)	S1(26;21)
16	33	69.1	12.38 (0.44)	-0.69 (0.046)	S4(33;25)
17	31	71.9	17.65 (0.43)	-0.39 (0.054)	S2(24;14)
18	30	74.5	20.93 (0.50)	-0.69 (0.035)	S1(30;9)

Only the most populated clusters up to 75% cumulative contribution are listed.
[1]number of cluster elements
[2]cumulative percentage of total conformations
[3]average end-to-end distances calculated between Cα atoms of first and last substrate residue; standard errors are given in parentheses.
[4]average orientation of substrate relative to substrate fragment (chain E) in 4JMY from scalar product (cosθ) between normalized end-to-end vector for reference fragment and normalized vector between residues 2 and 9 for substrate; standard errors are given in parentheses.
[5]substrates participating in cluster; number of elements and longest lifetime in 100 ps intervals are given in parentheses (e.g. S8(24;5) means that substrate 8 contributed 24 elements to the cluster with the longest lifetime of 5*100 ps=500 ps); substrates participating in a cluster less than five times are omitted.
