# Supplementary material for: Varying molecular interactions explain aspects of crowder-dependent enzyme function of a viral protease
Source: PLoS Comput Biol. 2023 Apr 25;19(4):e1011054. doi: 10.1371/journal.pcbi.1011054 (PMC10162569; doi:10.1371/journal.pcbi.1011054)
Supplement: S10 Table — (PDF) [file pcbi.1011054.s041.pdf]

S10 Table Cluster analysis for substrate binding near the active site in simulations with PEG crowders

#	N[1]	cum%[2]	End2end[3] [Å]	cosθ[4]	substrates[5]
1	341	13.7	14.52 (0.12)	0.80 (0.005)	S9(341; 41)
2	187	21.1	15.44 (0.20)	0.07 (0.022)	S4(183; 62)
3	149	27.1	12.38 (0.10)	0.04 (0.015)	S9(147;53)
4	139	32.7	13.85 (0.21)	-0.05 (0.024)	S5(137; 108)
5	137	38.2	17.49 (0.28)	-0.20 (0.024)	S5(67; 46) S6(54; 47) S9(14;6)
6	134	43.5	17.98 (0.14)	0.61 (0.013)	S9(129; 70)
7	117	48.2	15.65 (0.25)	0.40 (0.014)	S9(116; 61)
8	116	52.8	17.69 (0.25)	-0.86 (0.010)	S7(94; 25) S4(15; 6) S5(5;3)
9	105	57.0	19.05 (0.31)	0.02 (0.028)	S9(77;38) S2(20;20) S5(5;3)
10	101	61.1	15.70 (0.30)	0.04 (0.037)	S9(85;67) S5(6;2) S2(5;2)
11	85	64.5	18.61 (0.23)	-0.27 (0.024)	S9(79;25)
12	78	67.6	18.03 (0.29)	0.38 (0.024)	S4(78;39)
13	70	70.4	17.25 (0.42)	-0.85 (0.017)	S4(69;47)
14	66	73.1	17.68 (0.37)	-0.64 (0.027)	S4(38;21) S7(15;2) S9(10;4)

Only the most populated clusters up to 75% cumulative contribution are listed.
[1]number of cluster elements
[2]cumulative percentage of total conformations
[3]average end-to-end distances calculated between Cα atoms of first and last substrate residue; standard errors are given in parentheses.
[4]average orientation of substrate relative to substrate fragment (chain E) in 4JMY from scalar product (cosθ) between normalized end-to-end vector for reference fragment and normalized vector between residues 2 and 9 for substrate; standard errors are given in parentheses.
[5]substrates participating in cluster; number of elements and longest lifetime in 100 ps intervals are given in parentheses (e.g. S8(24;5) means that substrate 8 contributed 24 elements to the cluster with the longest lifetime of 5*100 ps=500 ps); substrates participating in a cluster five or less times are omitted.
