# Supplementary material for: Varying molecular interactions explain aspects of crowder-dependent enzyme function of a viral protease
Source: PLoS Comput Biol. 2023 Apr 25;19(4):e1011054. doi: 10.1371/journal.pcbi.1011054 (PMC10162569; doi:10.1371/journal.pcbi.1011054)
Supplement: S11 Table — (PDF) [file pcbi.1011054.s042.pdf]

S11 Table Cluster analysis for substrate binding near the active site in simulations with Ficoll crowders

#	N[1]	cum%[2]	End2end[3] [Å]	cosθ[4]	substrates[5]
1	297	12.3	20.86 (0.16)	-0.93 (0.003)	S1(295;99)
2	269	23.5	21.16 (0.15)	0.76 (0.010)	S5(230;60) S4(29;29) S8(10;7)
3	257	34.1	20.29 (0.11)	-0.86 (0.004)	S1(237;90) S7(20;17)
4	231	43.7	18.26 (0.35)	0.86 (0.007)	S5(221;90)
5	178	51.1	15.52 (0.20)	-0.78 (0.008)	S1(175;91)
6	123	56.2	17.23 (0.37)	0.71 (0.011)	S1(122;35)
7	92	60.0	14.98 (0.36)	-0.19 (0.056)	S6(70;69) S3(7;2) S1(6;3) S7(5;5)
8	87	63.6	11.75 (0.31)	-0.16 (0.043)	S1(87;51)
9	86	67.2	19.98 (0.45)	-0.16 (0.037)	S7(54;53) S1(23;8) S4(6;3)
10	84	70.6	19.23 (0.14)	-0.16 (0.037)	S1(84;73)
11	83	74.1	12.53 (0.44)	-0.02 (0.033)	S1(83;40)

Only the most populated clusters up to 75% cumulative contribution are listed.
[1]number of cluster elements
[2]cumulative percentage of total conformations
[3]average end-to-end distances calculated between Cα atoms of first and last substrate residue; standard errors are given in parentheses.
[4]average orientation of substrate relative to substrate fragment (chain E) in 4JMY from scalar product (cosθ) between normalized end-to-end vector for reference fragment and normalized vector between residues 2 and 9 for substrate; standard errors are given in parentheses.
[5]substrates participating in cluster; number of elements and longest lifetime in 100 ps intervals are given in parentheses (e.g. S8(24;5) means that substrate 8 contributed 24 elements to the cluster with the longest lifetime of 5*100 ps=500 ps); substrates participating in a cluster five or less times are omitted.
